# The impact of human breast milk components on the infant metabolism

**DOI:** 10.1371/journal.pone.0197713

**Published:** 2018-06-01

**Authors:** Christian Hellmuth, Olaf Uhl, Hans Demmelmair, Maria Grunewald, Renata Auricchio, Gemma Castillejo, Ilma R. Korponay-Szabo, Isabel Polanco, María Roca, Sabine L. Vriezinga, Katharina J. Werkstetter, Berthold Koletzko, M. Luisa Mearin, Franca F. Kirchberg

**Affiliations:** 1 Ludwig-Maximilians-Universität München, Division of Metabolic and Nutritional Medicine, Dr. von Hauner Children’s Hospital, University of Munich Medical Center, Munich, Germany; 2 Department of Medical Translational Sciences and European Laboratory for the Investigation of Food-Induced Diseases, University Federico II, Naples, Italy; 3 Department of Pediatric Gastroenterology Unit, Hospital Universitari Sant Joan de Reus, URV, IIPV, Reus, Spain; 4 Celiac Disease Center, Heim Pál National Pediatric Institute, Budapest, Hungary and Department of Pediatrics, University of Debrecen, Debrecen, Hungary; 5 Department of Pediatric Gastroenterology and Nutrition, La Paz University Hospital, Madrid, Spain; 6 U. Enfermedad Celiaca e Inmunopatología Digestiva, Instituto de Investigación Sanitaria La Fe, Valencia, Spain; 7 Department of Pediatrics, Leiden University Medical Center, Leiden, The Netherlands; TNO, NETHERLANDS

## Abstract

**Background & aims:**

Breastfeeding is beneficial for mothers and infants. Underlying mechanisms and biochemical mediators thus need to be investigated to develop and support improved infant nutrition practices promoting the child health. We analysed the relation between maternal breast milk composition and infant metabolism.

**Methods:**

196 pairs of mothers and infants from a European research project (PreventCD) were studied. Maternal milk samples collected at *month 1* and *month 4* after birth were analysed for macronutrient classes, hormone, and fatty acid (FA) content. Phospholipids, acylcarnitines, and amino acids were measured in serum samples of 4-month old infants. Associations between milk components and infant metabolites were analysed with spearman correlation and linear mixed effect models (LME). P-values were corrected for multiple testing (*P*_LME_).

**Results:**

*Month 1* milk protein content was strongly associated with infant serum lyso-phosphatidylcholine (LPC) 14:0 (*P*_LME_ = 0.009). *Month 1* milk insulin was associated to infant acetylcarnitine (*P*_LME_ = 0.01). There were no associations between milk protein content and serum amino acids and milk total fat content and serum polar lipids. Middle- and odd-chain FA% in breast milk at both ages were significantly related to serum LPC and sphingomyelins (SM) species in infant serum (all *P*_LME_<0.05), while FA% 20:5n-3 and 22:6n-3 percentages were significantly associated to serum LPC 22:6 (*P*_LME_ = 1.91×10^−4^/7.93×10^−5^) in milk only at *month 4*. Other polyunsaturated fatty acids and hormones in milk showed only weak associations with infant serum metabolites.

**Conclusions:**

Infant serum LPC are influenced by breast milk FA composition and, intriguingly, milk protein content in early but not late lactation. LPC 14:0, previously found positively associated with obesity risk, was the serum metabolite which was the most strongly associated to milk protein content. Thus, LPC 14:0 might be a key metabolite not only reflecting milk protein intake in infants, but also relating high protein content in milk or infant formula to childhood obesity risk.

## Introduction

Compared to infant formula feeding, breastfeeding (BF) is beneficial for mothers but particularly for their infants, for example with respect to reduced risks for infections and, with less clinical evidence to date, to later obesity and diabetes type 2, and promoting cognitive development [1–4]. Promoting BF and an adequate BF duration is strongly recommended to support child health and development [5]. Several studies have described the effects of maternal dietary fat intake during lactation or milk fatty acid (FA) composition on offspring health, e.g. growth [6–8], neurodevelopment [9, 10], and the risks of atopic diseases [11, 12] and allergy [13, 14], but little is known on the direct effect on infant metabolism. The few association studies are limited to fatty acid percentages (FA%) of maternal milk in relation to FA% in the infant blood [12, 15–17]. Despite providing a potential mechanism linking milk and disease, those and other studies are subject to limitations since they only describe FA% in infant serum and give no information on the exact molecular structure of the affected lipid molecules. Having the exact information on chemical bonds and backbones would enable to describe in details the affected pathways. We have previously shown that dietary polyunsaturated FA (PUFA) provided to infants are dominately incorporated into phosphatidylcholine species containing an ether bond (PCae) [18]. These lipids exhibit different chemical and physiological properties compared to the major class of diacyl-linked phosphatidylcholines (PCaa) or triacylglycerols [19], highlighting the importance to determine the molecular structure of the lipids.

One of the possibilities is to determine a wide range of small molecules (<1.5 kDa) with mass spectrometry protocols. Its utilization for mechanistic studies in early life is increasing as it offers the opportunity to determine alterations in the metabolism by environmental exposures [20]. Kirchberg et al. demonstrated that the intake of infant formula (IF) with a high protein content resulted in increased amino acid (AA) and short chain acylcarnitine (AC) levels in 6 month old infants as well as a saturation of branched-chain AA (BCAA) degradation pathway and a decrease of FA oxidation [21]. In the same study, the lyso-phosphatidylcholine (LPC) 14:0 was the only identified metabolite related to both early weight gain (WG) and obesity risk at 6 years of age [22]. Other studies found aromatic amino acids (AAA) and BCAA to be associated to obesity and insulin resistance development in adults, although huge controversy exists regarding this topic, since studies in childhood could not reproduce this finding or just found single AA, like tyrosine, being associated to insulin resistance [23–26].

The aim of this study was to analyse associations of maternal milk components at *month 1* and *month 4* to infant lipid and amino acids metabolism at 4 months of age. We used pre-intervention samples from a European cohort for the study of coeliac disease (CD) prevention, the PreventCD study [27]. We have previously shown that infant metabolites were not altered due to later CD development or maternal human leukocyte antigen status [28]. Therefore, we could use this study and its unique bio-sample availability to depict how early and late maternal milk composition influence infant metabolites.

## Material and methods

### Study design

This is a secondary analysis on 196 mother/infant pairs from PreventCD. PreventCD is a prospective, double-blind, placebo-controlled, randomized, dietary-intervention study in children with high risk for development of CD [27, 29] conducted in Croatia, Germany, Hungary, Israel, Italy, the Netherlands, Poland, and Spain. Infants between 0–3 months of age were recruited if they had at least one first-degree family member with CD confirmed by small-bowel biopsy, and if they were HLA-DQ2 or HLA-DQ8 positive or carried the allele DQB1*02. Since later CD development and HLA-risk group assignment had no influence on the serum metabolites [28] and the intervention was applied after blood withdrawal, these variables were not investigated in the present study.

The study was registered at ISRCTN on February 26th, 2007 and the first / last child was included on May 26th, 2007 / September 25th, 2010, respectively. Prematurity or syndromes associated with an increased risk of CD, such as trisomy 21 or Turner’s syndrome, were exclusion criteria. Between 4 and 6 months of age, infants received either 200 mg of vital wheat gluten mixed with 1.8 g of lactose) or 2g of lactose per day. All infant serum samples used for the present study were withdrawn before this intervention started. Data collection and anthropometric measurements were performed as previously reported [27].

The study was approved by the medical ethics committee at each participating centre and complied with Good Clinical Practice guidelines. The study was conducted according to the Declaration of Helsinki. The parents of all children provided written informed consent for the study. The PreventCD Current Controlled Trials number is ISRCTN74582487. The authors confirm that all ongoing and related trials for this drug/intervention are registered. Ethical committee review boards (with respective date of ethical approval): Commissie Medische Ethiek LUMC, Leiden, the Netherlands (December 1^st^, 2006); Comitato Etico, Universita degli studi di Napoli ‘Fedrico II’, Naples, Italy (October 12^th^, 2006); Komisja Bioetyczna przy Warszawskim Uniwersytecie Medycznym, Warsaw, Poland (July 17^th^, 2006); Provecto de Investigacion, La Paz, Madrid, Spain (November 7^th^, 2006); Instituto de Invest Investigacion, La Fe, Valencia, Spain (December 19^th^, 2006); Rabin-Schneider IRB, Tel Aviv, Israel (October 4^th^, 2006); Etičkog povjerenstva. Zagreb, Croatia (February 17^th^, 2006); Institutional Research Ethics Committee, Heim Pal Childrens Hospital, Budapest, Hungary (March 6^th^, 2007); Comite d’Etica d’Investigacio Clinica, Reus, Spain (February 22^nd^, 2007); Ethikkomimision LMU, Munich, Germany (May 21^st^, 2007).

### Sample collection

Blood samples were collected from the infants at the 4-month visit. After collection, the blood was centrifuged and the frozen serum samples were stored at -20°C. Subsequently, the serum samples were aliquoted and sent to the central laboratory for antigliadin antibody (AGA) and transglutaminase type2 antibody (TG2A) determinations. The rest sera was transported and re-stored at -20°C at the sera bank of the project at the department of Immunohematology and Blood Transfusion, Leiden University Medical Center, Leiden. Samples chosen for this study were transferred on dry ice to LMU Munich and stored at -80°C until mass spectrometry-based analysis of metabolites.

Maternal milk samples, used in the presented analysis, were collected at visits 1 and 4 months after birth. Mothers expressed their milk manually or by pump once a month during the first six months after birth. No further specification to collect fore- or hind-milk sampling and time of day were given to the mothers. The milk samples were frozen at -20° C at home, transferred to the hospital on ice and stored at—80° C. Milk samples for the presented analysis were aliquoted (1–2 ml), transferred on dry ice to LMU München and stored at -80°C until analysis.

#### Selection of samples

In order to obtain complete sample sets, only those mother/infant pairs were included in this sample analyses who had both an infant serum sample at month 4 and at least 1 corresponding maternal milk sample at *month 1* or *month 4* available.

### Breast milk analysis

Maternal milk samples were analysed for macronutrients classes (fat, protein) as well as FA% and hormones (insulin, IGF-II, adiponectin, leptin).

For fat analysis, milk samples were diluted 1:3 with water and sonicated and heated to 40° C prior to analysis. Total fat was measured via mid-infrared spectroscopy with a MIRIS Human Milk Analyzer (MIRIS HMA, Miris AB, Uppsala, Sweden) [30]. Protein content was measured with an adapted Bradford method as described previously [31]. FA composition, using 20 μl of milk, was analysed by gas chromatography (GC) as previously described [32]. The lipid bound FA were converted with acidic catalysis into FA methyl esters and extracted into hexane prior to GC analysis. Molecular percentages of 35 FA with 8 to 24 carbon atoms were determined (FA%).

Breast milk total adiponectin concentration was measured with a commercially available ELISA kit (Biovendor RD191023100 High Sensitivity Adiponectin, Brno, Czech Republic). 50 μl skimmed milk were diluted 1:3 with the dilution buffer provided with the test kit, and the procedure followed the protocol of the manufacturer. Leptin concentrations in skimmed milk were determined with an ELISA (human leptin; R&D Systems, Minneapolis, MN; Catalogue Number DLP00). 100 μl of skim milk were diluted 4-fold and the further analysis was performed according to the manufacturer‘s instructions.

Insulin concentrations were measured with the Mercodia Insulin ELISA kit 10-1113-01 (Mercodia, Uppsala, Sweden) from 25 μl undiluted, skimmed milk. The assay procedure was performed according to the protocol of the manufacturer. IGF-II was determined with a radioimmunoassay from 30 μl full fat milk by Mediagnost (Reutlingen, Germany) using the R-30 IGF-II RIA kit according to the protocol of the manufacturer.

### Infant serum metabolites

The infant metabolites were quantified as described previously [28]. As these are targeted protocols, we used internal and external standards for calibration and quantification of the targeted metabolites. A labelled amino acid standards set (NSK-A), 15N2 L-Asparagine (NLM-3286), Indole-D5 L-Tryptophan (DLM-1092), D3-Carnitine C2 (DLM-754), D3-Carnitine C8 (DLM-755), D3-Carnitine C16 (DLM-1263), 13C6-D-Glucose (CLM-1396), (all Cambridge Isotope Laboratories), 1-tridecanoyl-2-hydroxy-sn-glycero-3-phosphocholine (855476P), and 1,2-dimyristoyl-sn-glycero-3-phosphocholine (850345P), (both Avanti Polar Lipids) were used as internal standards. For calibration, we used a amino acid standard mixture (A9906) from Sigma Aldrich, L-Asparagine anhydrous (11150) from Fluka, L-Glutamine (G3126) from Sigma Aldrich and a control plasma (10282) from Recipe.

For amino acid (AA) analysis, 10μl of serum was used for derivatization to AA butylester, which were separated by ion-pair liquid chromatography (LC). The LC system (Agilent, Waldbronn, Germany) was coupled to a tandem mass spectrometer (MS/MS, QTRAP4000, Sciex, Darmstadt, Germany) with an atmospheric pressure chemical ionization source operating in positive ionization mode. The MS was run in positive Scheduled Multiple Reaction Monitoring (SMRM) mode. For quantification of polar lipids, 10μl serum were diluted with methanol and supernatants were injected into the LC-MS/MS system which was used as flow-injection analysis. The MS was run in MRM mode in both positive and negative ionization mode. We determined free carnitine, acylcarnitines (AC), lysophosphatidylcholines (LPC), diacyl-phosphatidylcholines (PCaa), acyl-alkyl-phosphatidylcholines (PCae), and sphingomyelines (SM). The structures of the hydrocarbon chains of the polar lipids are indicated by the denotation Cx:y where x and y denote the length of the hydrocarbon chain and the number of double bonds, respectively. ‘a’ indicates that the chain is bound via an ester bond to the backbone, while ‘e’ indicates binding by an ether bound. ‘OH’ stands for an additional hydroxyl group and ‘DC’ for two carboxyl groups in the molecule.

During the quantification processes, the samples of the study centres were randomly distributed across the batches. The entire analytical process was post-processed by the software Analyst 1.5.1 and R 3.2.1. All metabolite concentrations are reported in μmol/L.

### Statistics

Using six aliquots of one quality control (QC) samples that were measured along with the serum samples in each batch, we selected the metabolites for analysis. If the coefficient of variation of the QC samples was <30%, then the metabolite was included in the analyses. The batch effect was removed in the statistical models (see below). Afterwards, outliers (defined a measurement whose absolute concentration is over one standard deviation (SD) away from its nearest neighbour) were excluded from the data analysis.

For the analysis of the effect of *month 1* milk on the infant serum at 4 month, we included only milk samples that were (i) frozen during transport, and (ii) collected between infant age 6 and 47 days.

For the cross-sectional analysis between *month 4* milk and the infant serum at 4 month, we included only milk samples that (i) were frozen during transport, (ii) have been collected between infant age 106 and 134 days and (iii) that were collected before or at most three days after the corresponding infant blood sample was collected.

Associations between milk components and infant metabolites were analysed with spearman correlation (ρ) and linear mixed effect models (LME) with the milk compound as independent variable, adjusted for infant sex, breastfeeding status at blood withdrawal (exclusively BF yes/no), and the infant’s age at blood withdrawal. Random intercepts were modelled for batch number and study centre. Models were run on available cases and sample sizes are reported for each association. P-values were corrected (*P*_LME_) for multiple testing using Bonferroni’s methods, this is by dividing the p-value with number of metabolites (n = 184). A *P*_LME_ < 0.05 is considered as statistically significant. If only the uncorrected p-value was <0.05, we speak of a trend. We additionally report the estimate of the LME (B_LME_). For visualization, circled Manhattan plots and heatmaps were used. The Manhattan plots were used for milk hormones and macronutrient classes associations to serum metabolites, which were arranged in a circle. Negative log_10_-transformed uncorrected p-values were plotted for associations between single metabolites and hormones/macronutrients classes with higher values presenting stronger associations in the outer part of the circle. The Bonferroni corrected significance threshold is indicated by the black outer circle. Heatmaps were utilized to visualize associations between milk FA% (y-axis) and single metabolites (x-axis). Spearman correlation coefficients were plotted with red indicating negative ρ and green positive ρ.

As milk components as well as serum metabolites may strongly correlate with each other, we performed sensitivity analyses by manually checking the effects in LME: For serum metabolites that were associated either to more than one milk macronutrient classes/hormone or to at least one milk macronutrient classes/hormone and milk FA%, we regressed the serum metabolite (outcome) on the respective macronutrient classes, hormone or fatty acid variables. We adjusted each model for infant sex, breastfeeding status at 4-month blood withdrawal (exclusively BF yes/no), and the infant’s age at blood withdrawal. Random intercepts were modelled for batch number and study centre. This model was additionally calculated with interaction effect between the determinants. Multicollinearity was checked using variance inflation factors (VIF). A VIF of 4 was considered as indicator for multicollinearity.

Additionally, a subgroup analysis was conducted on infants who were exclusively breastfed until the 4-month blood withdrawal. For this analysis, all statistical models mentioned above were performed, again.

## Results

**Fig 1:**
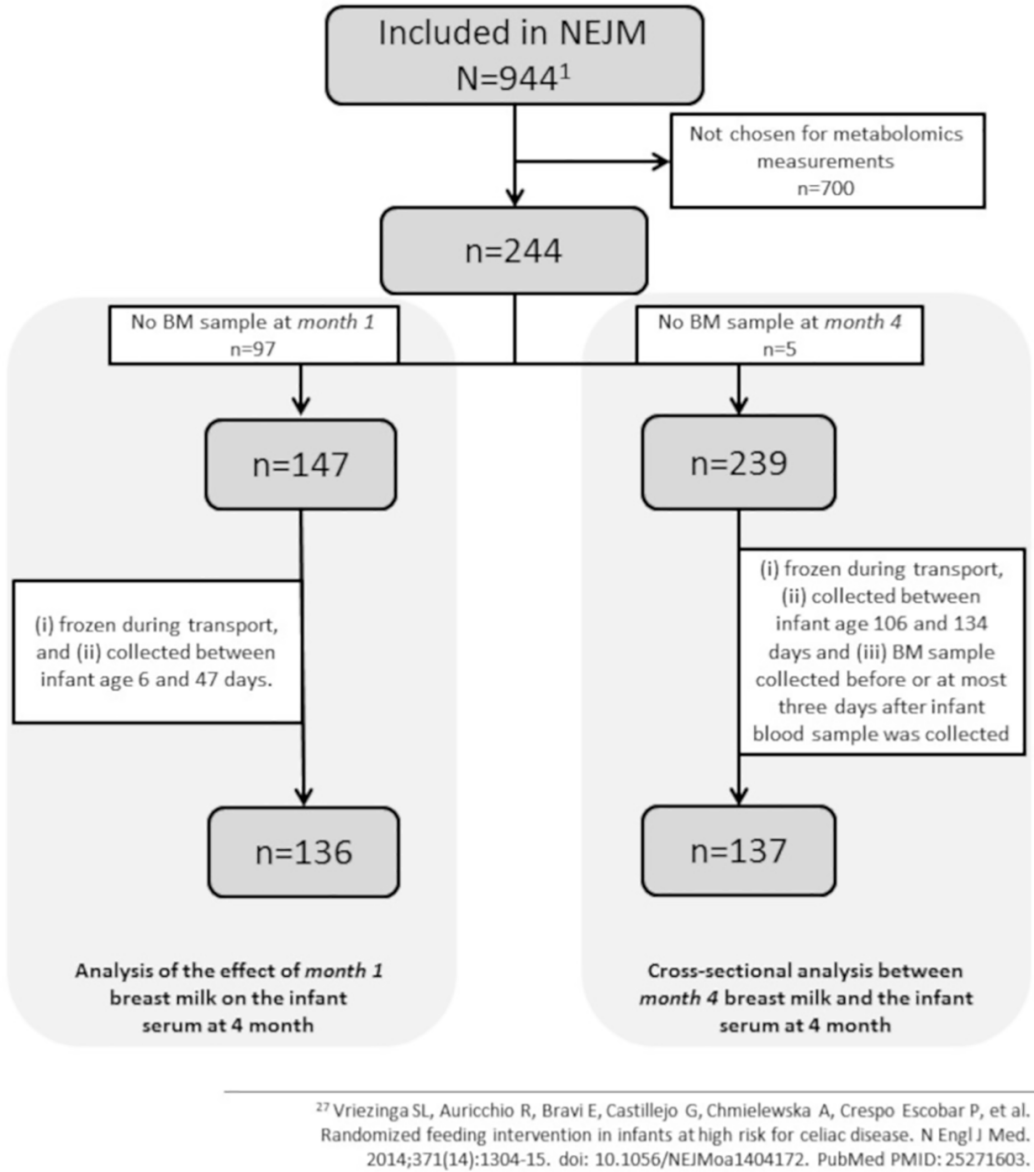
CONSORT flow diagram. Of 944 children in the PreventCD-cohort, 196 complete mother/infant pairs with complete sample sets were analysed. 136 pairs were studied for the associations between *month 1* breast milk composition and infant serum metabolites at age of 4 months and 137 were studied for the associations between *month 4* breast milk composition and infant serum metabolites at age of 4 months. 87 were studied at both time points.

Of 944 children in the PreventCD-cohort, 196 complete mother/infant pairs with complete sample sets were analysed (Fig 1). Their characteristics are described in Table 1. Hundred thirty-six pairs were studied for the associations between *month 1* breast milk composition and infant serum metabolites at age of 4 months. Hundred thirty-seven pairs were studied for the associations between *month 4* breast milk composition and infant serum metabolites at age of 4 months. Seventy-eight pairs were studied at both time points. Hundred eighty-four serum metabolites were quantified in infants aged 4 months. Analysis was conducted in all children (including exclusively and non-exclusively BF at 4 month) and in children who were exclusively breastfed at 4 months. We observed no difference between these analyses and will present the analysis on all children (S1 Fig).

**Table 1:** Characteristics of the infant/mother pairs studied with breast milk samples available at month 1 and month 4.

	*Month 1* BM (n = 136)		*Month 4* BM (n = 137)	
	N	Mean ± SD or n		Mean ± SD or n
*Sex (Female)*	136	69	137	61
*Age mother at birth (years)*	132	33.6 ± 3.57	132	33.4 ± 3.87
*Age infant at serum sampling (days)*	136	120.3 ± 7.45	137	123.1 ± 5.59
*Age infant at milk withdrawal (days)*	136	27.8 ± 8.50	137	118.3 ± 5.65
*Birthweight (kg)*	136	3.4 ± 0.47	136	3.3 ± 0.43
*Exclusively breastfed 4 month*	136	119	137	116
*Country of Study Centres*	136		137	
Netherlands		30		23
Italy		9		3
Spain		32		40
Hungary		30		37
Germany		35		34

### Breast milk macronutrient classes

**Fig 2:**
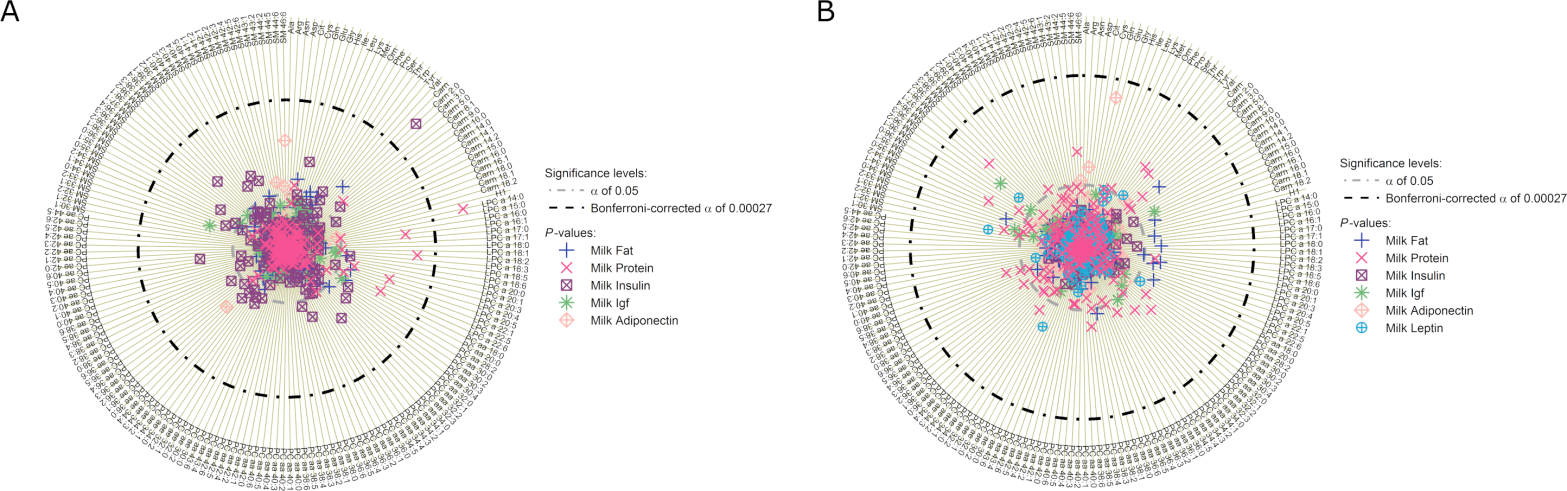
Associations between breast milk macronutrient classes and hormones to infant serum metabolites at 4 months of age. Breast milk components were measured at month 1 (a) or month 4 (b). Negative log-transformed P-values are plotted for each metabolite arranged by metabolite group and species. Higher values represented in the outer circles present a higher association between metabolite and predictor. P-values were calculated by linear regression models with the milk compound as independent variable, adjusted for infant sex, breastfeeding status at 4-month blood withdrawal (exclusively BF yes/no), and the infant’s age at blood withdrawal. Random intercepts were modelled for batch number and study centre. P-values were corrected (PLME) for multiple testing using Bonferroni’s methods, this is by dividing the p-value with number of metabolites (n = 184).

Results (Spearman’s ρ and results of LME) for maternal milk macronutrient classes association to infant serum are shown in Fig 2 and S1 Table. Milk protein showed only one significant association. *Month 1* milk protein content was strongly associated with LPC 14:0 in the infant blood serum at 4 months of age (ρ = 0.291 B_LME_ = 1.4, *P*_LME_ = 9.0 x10^-3^). Only when considering the unadjusted significance threshold, several other LPC in infant serum tended to be positively associated with *month 1* milk protein content: LPC 15:0, 16:1, 18:1, 18:2, 18:3, 20:3, 20:4, 22:5, and 22:6 (ρ = 0.077–0.252). An association between serum AA to *month 1* or *month 4* milk protein was not observed.

Total fat content in milk was not significantly associated to serum metabolite profile at either month after correction for multiple testing.

### Breast milk fatty acid composition

**Fig 3:**
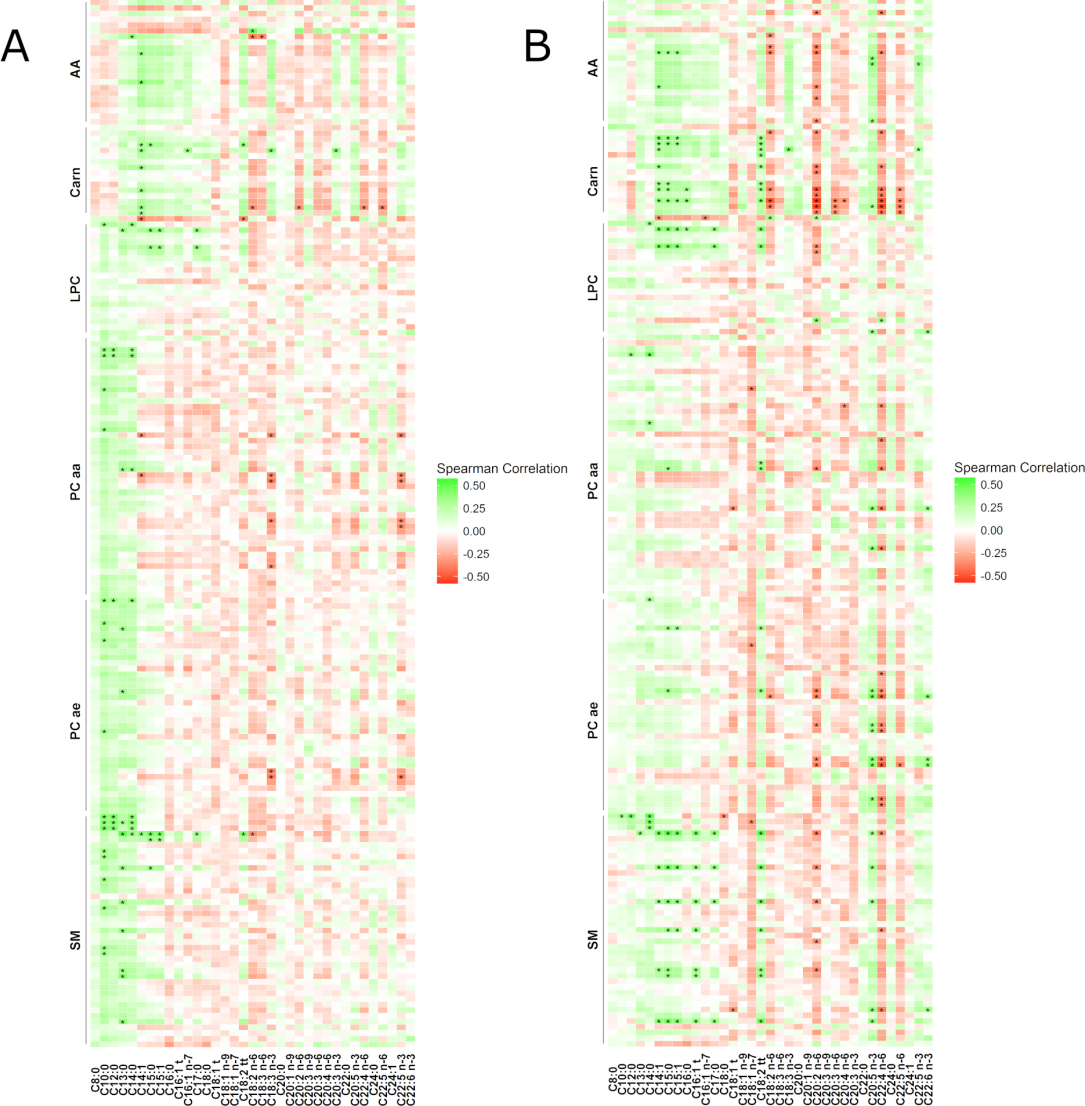
Correlations between breast milk fatty acids percentages to infant serum metabolites at 4 months of age. Breast milk components were measured at month 1 (a) or month 4 (b). Spearman correlation coefficients are plotted for each metabolite arranged by metabolite group. AA, amino acids; Carn, acylcarnitines; LPC, lysophosphatidylcholines; PC aa, diacyl-phosphatidylcholines; PC ae, acyl-alkyl-phosphatidylcholines SM, sphingomyelins.

Percentages of maternal milk FA were correlated and associated to the several lipid derivate metabolites in the serum of the infants aged 4 months (Fig 3, S2 Table). Particularly, *month 1* and *month 4* middle- (C10-C14) and odd-chain (C13, C15, C17) FA% in milk showed significant associations.

*Month 1* middle-chain FA% (chain-length of 10 to 14 carbon atoms) in milk were significantly correlated to LPC 14:0 in infant serum, the shortest LPC measured, as well as to SM and PC species, in particular to those with 30 to 34 carbon atoms in infant serum (ρ = 0.328–0.455). However, in the adjusted LME, only FA% 14:0 in *month 1* was found to be significantly associated to LPC 14:0 in infant serum (B_LME_ = 15.0, *P*_LME_ = 4.4 x10^-2^). *Month 1* odd-chain FA% in milk were correlated and associated to LPC 15:0 and 17:0 (ρ = 0.331–0.380, *P*_LME_ = 2.6x10^-6^–4.6x10^-4^) and SM species with an odd numbered carbon chain length (ρ = 0.322–0.499, *P*_LME_ = 9.6x10^-5^–4.6x10^-2^) in spearman correlation and LME associations studies. The FA% 18:2n-6, 20:2n-6, 22:4n-6 and 22:5n-6 in *month 1* milk were negatively correlated to AC 18:1 (ρ = -0.363-(-0.331)). No significant association for AC with *month 1* milk component were observed in the LME.

*Month 4* middle-chain FA% 12:0 and 14:0 in milk were correlated to LPC 14:0, and all middle-chain FA% were correlated and associated to PC and SM with 30–32 Carbon atoms (ρ = up to 0.488, *P*_LME_ = down to 3.75x10^-11^) in infant serum. *Month 4* odd-chain FA% in milk were related to LPC 15:0 and 17:0 and SM species with an odd-chain number of carbon atoms in spearman correlation and LME associations studies (ρ = up to 0.504, *P*_LME_ = down to 3.68x10^-8^). M*onth 4* FA% 18:0, 18:1n-7, 20:2n-6, and 22:4n-6 showed negative correlations to LPC, SM and PC (ρ = down to -0.475) but fewer significantly negative associations in the LME. *Month 4* FA% 20:5n-3, 22:5n-3 and 22:6n-3 in milk were correlated and associated to LPC 22:6 (B_LME_ = 906.0/876.5/353.0, *P*_LME_ = 2.21x10^-4^/2.20x10^-2^/9.55x10^-5^, respectively) and 20:5n-3, and 22:6n-3 were significantly associated to PCaa 38:6, 42.4, PCae 38:0, and SM 42.6.

The FA% 18:2n-6, 20:2n-6, 20:3n-6, 22:4n-6 and 22:5n-6 in *month 4* milk were negatively correlated to several AC (ρ = down to -0.544), while 14:1, 15:0. 15:1, 16:0 and 18:2tt showed positive correlations (ρ = up to 0.424). No significant association for AC with *month 4* milk FA% were observed in the LME.

### Breast milk hormones

The determined maternal milk hormones showed only weak association with infant serum metabolites (Fig 2, S3 Table). Only the associations between *month 1* milk insulin and acetylcarnitine (AC 2:0) were significant after correction for multiple testing (ρ = 0.242, B_LME_ = 0.056, *P*_LME_ = 0.009). In general, several serum phospholipids tended to be positively associated to *month 1* milk insulin, if correction for multiple testing was ignored. *Month 1* and *month 4* milk IGF-2, and adiponectin and *month 4* milk leptin showed no significant associations.

### Correlation of breast milk fatty acids

Respectively at *month 1 and month 4*, saturated middle-chain FA% in breast milk correlated strongly positively with each other and trans-FA% 16:7t and 18:2tt, but negatively with FA% 18:1n-7, 18:1n-9 and n-6 FA% (S1 Fig). Maternal milk odd-chain FA% were also strongly correlated. FA% 14:1 was correlated to all FA% with 15, 16, or 17 carbon atoms, but not to the saturated milk middle-chain FA% at both time points, respectively. Milk n-6 FA% showed positive correlations among each other, like the n-3 FA% 20:5 and 22:6.

### Sensitivity analysis

Only one infant serum metabolite was affected by several breast milk nutrients. We ran LME models with interaction effects to investigate these associations closer.

Serum LPC 14:0 was associated to *month 1* milk protein and *month 1* milk FA% 14:0. In the combined multivariable model (LPC 14:0 ~ *month 1* milk protein + *month 1* milk FA% 14:0+ confounders), only *month 1* milk protein (B_LME_ = 1.27, *P*_LME_ = 3.1 x10^-2^) remained statistically significant, but *month 1* milk FA% 14:0 was borderline significantly associated to LPC 14:0 (B_LME_ = 13.5, *P*_LME_ = 0.08). No multicollinearity was observed (VIF = 1.01). The effects of *month 1* milk protein and FA% 14:0 on serum LPC 14:0 were of equal magnitude: An increase of one standard deviation of *month 1* milk protein would results in an increase of 0.28 μM of serum LPC 14:0, while an increase of one standard deviations of *month 1* milk FA% 14:0 would results in an increase of 0.26 μM of serum LPC 14:0 (mean_LPC 14:0_ = 1.66 μM, sd_LPC 14:0_ = 0.91 μM). Explained variance by all independent variables together was R^2^ = 75%. No significant interaction between *month 1* milk protein and *month 1* milk FA% 14:0 was observed (*P*_LME_ = 0.61). Both components did not correlate either (ρ = 0.080).

## Discussion

We studied the association of components of human breast milk collected one or four months after birth with the infant serum metabolites at 4 month of age. Infant feeding is considered to play a crucial role in the later development of non-communicable disease. Studies collecting a large number of breast milk samples and corresponding infant blood samples offer an exclusive opportunity to elucidate the effect of milk components. We used maternal milk and infant serum samples available from the PreventCD study to assess the effects of milk on infant metabolites in mother/infant pairs in a longitudinal (*month 1* milk ~ month 4 infant metabolites) and a cross sectional approach (*month 4* milk ~ month 4 infant metabolites). In the longitudinal approach, the association of protein breast milk content and LPC 14:0 is striking. While fat and hormone breast milk content showed only a very weak effect on the infant metabolites, the milk FA% showed strong associations to infant serum phospholipid species, but in a PL species specific manner.

### Milk macronutrient classes and hormones

*Month 1* maternal milk protein was strongly associated to month 4 infant serum LPC 14:0, and, to a lesser extent, to other LPC. However, this effect could not be replicated for *month 4* breast milk. Probably the much higher protein concentration in milk shortly after birth has a stronger and longer effect on the serum LPC, compared to the reduced milk protein amount later during lactation [33]. In the European Childhood Obesity Programme (CHOP) study, infant serum LPC 14:0 at 6 month of age was significantly associated with both rapid weight gain in the first 6 months of life as well as overweight/obesity at 6 years [22]. In the CHOP study, infants were randomly allocated to receiving an IF containing conventionally high protein (HP) or an IF with reduced protein content. In this context, when referring to the only published study having found LPC 14:0, one has to note that IF in the first month of life is not following the natural decline of protein in milk. It may thus have much stronger effects on LPC 14:0, and the potential adverse effects of the LPC. The metabolites in infant blood which were expected to be influenced by higher protein intake and to affect infant metabolism were BCAA. However, these AA were not related to maternal milk protein in the presented study, not even by trend. Thus, LPC 14:0 seems to present a more reliable candidate for the programing mechanism linking early protein intake and childhood obesity risk. But how is protein in milk or IF affecting this metabolite? To answer this question, mechanistic studies are needed. We can only speculate on the pathways. There is a general effect of *month 1* breast milk protein on LPC, since the five strongest associations of *month 1* milk protein to *month 4* infant metabolites are all with LPC (14:0, 18:2, 16:1, 20:4, 22:6). LPC are a product of hydrolysis of phosphatidylcholines by phospholipases [34] or endothelial lipases [35], hepatic secretion [36] or lecithin cholesterol acyltransferase (LCAT). LCAT, which produces LPC and cholesterol esters by transferring a FA from a PC to free cholesterol, is the dominating enzyme for LPC formation [37]. Human LCAT preferentially transfers the FA at sn-2 position of PC to cholesterol [38], although the sn-1 position is utilized in case the sn-2 chain is very long (C20:4, C22:6) [39]. Thus, LPC in the infant serum are most likely a product of LCAT activity which is also regulated by the diet [40–43]. In general, lower protein intake results in lower LCAT activity [44, 45] which supports our findings. Thus, the association of milk protein to LPC is not surprising, but the accentuated relation to LPC 14:0 is still posing challenges. Maternal milk FA% 14:0 did not correlate with milk protein concentration and both components showed no interaction effect in a multivariable model. This fact points towards an independent effect of milk protein on serum LPC 14:0. Potentially, breast milk protein is acylated with FA 14:0 to a higher degree. Since this would only affect small concentrations, an effect of milk protein on the metabolism of middle-chain fatty acids in infants is more reasonable. Dietary protein was shown to increase the expression of lipogenic enzymes in the muscle of piglets [46, 47], while a high protein diet decreased lipogenic activity in adipose tissue of pigs [48]. Whey protein and soy protein suppressed hepatic fatty acid synthesis and accelerated fat oxidation in rodents [49, 50]. Thus, dietary protein seems to have a tissue-specific effect on fat and FA metabolism—additionally depending on the quality of the protein. Drawing conclusion from our trial is challenging in this context since we measure blood metabolites, influenced by metabolism of different tissues. Another challenge in the interpretation represents our finding that the LPC 14:0 concentration in infant serum 4 month after birth was influenced by *month 1* maternal milk protein only and not by *month 4* milk protein. Having speculated on the mechanisms provoking the high infant LPC 14:0 levels, the question on what potentially makes this metabolite deleterious now imposes itself. The degradation product of LPC, lysophosphatidic acid (LPA) [37], is a lipid mediator acting via G-protein coupled receptors [51]. LPA was shown to impair glucose homeostasis and inhibit insulin secretion [52, 53] and is associated to inflammation processes [54, 55] and obesity [56].

Next to the month 1 protein to serum LPC 14:0 association, the association of month 1 insulin to serum acetylcarnitine was the only other significant relationship found between breast milk macronutrient classes or hormones and any infant metabolite. In general, insulin suppresses hepatic acetyl-CoA production [57], but in this case the situation is different since we have to speculate about potential effects of breast milk insulin. Once taken up by the infant, breast milk insulin has to survive the gastrointestinal digestions, but orally administered insulin was found to be rapidly destroyed by trypsin activity in adults [58]. Probably, trypsin activity is reduced in infants and insulin may reach the systemic circulation in infants. Also, protease inhibitors α1-antitrypsin and α1-antichymotrypsin are present in human breast milk and decline throughout lactation [59]. However, no data is available about trypsin activity on insulin in infants or children. Thus, it is difficult to speculate about milk insulin effects. A recent study found that breast milk insulin was associated with lower infant weight [60]. There results might fit our findings: if milk insulin somehow induces fat oxidation, as indicated by higher acetylcarnitine levels, this might lead to lower infant weight. This however is just a weak hypothesis, since serum insulin usually support fat deposition rather than oxidation. Another explanation could be a strong relation between maternal and infant blood insulin, by genetic background. Maternal blood insulin would effect maternal BM insulin, and infant blood insulin has effects on energy metabolism.

Regarding the weak associations between macronutrients classes (fat, protein) as well as milk hormones (insulin, adiponectin, leptin, IGF-II) and the infant metabolites, it is particularly striking that milk protein content and serum essential AA as well as milk fat content and serum phospholipids did not correlate significantly. Since we have no information about the amount of milk intake of the infants, it may be of more importance to determine the amount of breast milk macronutrients intake rather than their concentrations in the milk. One can easily assume that lower fat and protein results in lower caloric content of breast milk. As shown previously [61], this lower caloric amount is compensated by higher milk intake of the infant. Thus, breast milk macronutrient composition is a weak proxy for the actual macronutrient amount reaching the infant gut and affecting the infant metabolism and growth.

### Breast milk FA%

When investigating the FA species, the FA% gain importance as they reflect the composition of the FA in the milk. The *month 1* and *month 4* FA% in breast milk were strongly related to infant metabolites and especially to the PL. These relative compositions of milk FA were stronger associated to serum PL compared to the associations of total milk fat to PL, probably because the effect of the breast milk composition is independent of the absolute intake of FA. Odd-chain FA%, middle-chain FA% and, to a lesser extent, n-3 FA% in milk showed strong associations to infant PL. The stronger correlation of milk n-3 FA% to infant serum metabolites compared to milk n-6 FA% [15] in month 4 and the missing associations of long-chain saturated and mono-unsaturated FA% in milk to infant FA% were described previously [12, 13]. Kankaanpää et al showed that serum lipid fatty acids in atopic infants did not correlate with those in maternal breast milk [12], but other trials also report strong correlations between milk trans-FA%, n-3 FA%, and n-6 FA% and infant serum FA% of PL [12, 17, 62]. Jonsson et al. recently showed that maternal dietary intake of oily fish correlated with milk FA% 20:5n-3 [13]. FA% 20:5n-3 was further correlated to infant serum FA% 20:5n-3 at four months after birth which was found to be lower in infants developing allergy until the age of 3 years in the same study [13]. However, these previous trials analysed FA% in the blood of infants and not molecular species like we did with our mass spectrometry-based approach. The associations found in the present study were rather not an overall effect on all PL species, but affected specific structures in the PL molecules. This finding would probably not have been found when analysing FA% in blood of infants, since our measurements capture FA derived from PL. The main class of PL are PCaa. In our study, PCaa only showed few associations to milk FA %. Odd-chain FA% in milk collected at *month 1* and *month 4* were mainly associated with odd-chain LPC and SM in infant serum. Middle-chain LPC were only associated to middle-chain FA% of maternal milk collected at *month 1*, but not at *month 4*. In contrast, middle-chain FA% of maternal milk collected at *month 1* and *month 4* were associated to PC and SM containing middle-chain fatty acids. The more prominent association of middle-chain milk FA% to infant PL compared to the other milk FA% is most likely due to the better resorption of middle-chain FA [63].

Interestingly, we also observed negative associations between breast milk FA% and infant serum metabolites. In particular n-6 FA% in and *month 4* milk were negatively associated to LPC, PC, and SM species probably not containing the corresponding FA. Usually, the sn-1 position of PCaa is esterified with a saturated or monounsaturated species, and the sn-2 is occupied by a FA with higher unsaturation [64]. The observed negative associations thus speak for a competitive mechanism for incorporation of FA into PL. However, it has to be noted that milk FA% which were negatively related to infant serum PL did not show any positive associations to any PL species which might contain this FA. Hence, it is likely that these FA are incorporated in other lipids like triacylglycerols or cholesterol esters rather than PL. Kankaanppää et al. showed that milk FA% correlated mainly with cholesterol ester FA% and triacylglycerol FA%, and not PL, in the infant serum [12]. In the present study, we did not measure cholesterol esters or triacylglycerols, hence we are not able to verify the previous finding. Furthermore, we have shown that breast milk n-6 FA% at *month 1*, but in particular in *month 4*, were negatively associated to serum long-chain AC levels in 4 months old infants. Since AC are formed at the outer mitochondrial membrane to transport acyl chains into the mitochondria for oxidation, one could speculate about effects of dietary n-6 FA on fatty acid oxidation. Recent publications report an influence of dietary n-3 FA on fat oxidation [65, 66], but it is suggested that a reduction in dietary n-6/n-3 FA ratio is associated with higher fat oxidation [67] which is not in line with our results.

Regarding n-3 FA%, although the spearman correlation showed significant associations for some PCae species, in the adjusted models we only observed a positive effect on serum LPC 22:6. We previously described that 22:6n-3 in infant formula is preferentially incorporated into PCae species [18], possibly because PCae provide a unique structure to protect 22:6n-3 from oxidation and transport to the tissue [19]. When it comes to brain supply with 22:6n-3, MFSD2, the transporter of LPC 22:6, seems to play a crucial role. In a mouse model, a MFSD2 deficiency was related to lower brain 22:6n-3 levels and cognitive deficits [68]. Thus, in contrast to other PL LPC 22:6 exhibits a beneficial role, and represents a potential pathway linking breastfeeding and brain development. However, no explanation can be given why maternal milk FA% 22:6n-3 as well as odd-chain and middle-chain FA% are closely related to LPC and weaker to the PC.

Our study generates hypotheses guiding opportunities for future research. The unique design of the present study with its large number of mother/infant pairs with available milk and serum samples gives specific advantages in attempts to further elucidate the possible mechanisms of the beneficial effects of breast milk components and composition. To our knowledge we are the first to investigate the influence of maternal milk on a wide range of metabolites in the infant serum to this extent and in this much detail. Our targeted mass spectrometry-based platform depicts a comprehensive and representative number of metabolites involved in the lipid- and energy metabolism. However, an even larger sample number would have given us the opportunity to even dive deeper into the mechanisms initiated, like stratification for sex or analysis of the effect of breastfeeding duration.

The biggest challenge was the missing data about the amount of breast milk intake of the infant which may possibly have led to some missing or weak associations. Thus, we strongly recommend collecting this variable in future trials with breastfed infants. Furthermore, the milk samples were not completely standardized to collection of fore- or hint-milk or daytime, which is challenging since FA composition varies over the course of the day [69]. We also have variation in frequency of breastfeeding, parity, maternal diet and age, and the technique used to pump milk (manually or by pump). Additionally, the age of the infant at the “month 1 breast milk” sampling has a huge variation (6 to 47 days), which will strongly affect the milk composition [70, 71]. In the presented results, we reduced this influence by adjusting for sampling date. All these factors potentially influence our results in particular given the small number of relevant findings of our work. We strongly recommend standardized collecting variables in future trials with breastfed infants.

## Conclusion

The composition of the measured human milk components has only a modest influence on the infant’s metabolite serum levels at 4 month of age. We speculate that the amount of milk intake and the absolute intake of specific metabolites may have a more important influence on the infant’s metabolism, and that infants may be able to adjust their milk intake according to milk composition. However, especially serum LPC correlate by breast milk FA% and, most surprisingly, milk protein content in early lactation. LPC 14:0 was the most associated serum metabolite by milk protein content and was found to be positively associated to growth and obesity risk in previous studies. Thus, LPC 14:0 might be a key metabolite not only reflecting milk protein intake in infants but also relating high protein content in breast milk or infant formula to childhood obesity risk.

## Supporting information

S1 Fig Associations between breast milk macronutrient classes and hormones to infant serum metabolites at 4 months of age in the subgroup of exclusively breastfed infants. Breast milk components were measured at month 1 (a) or month 4 (b). Negative log-transformed P-values are plotted for each metabolite arranged by metabolite group and species. Higher values represented in the outer circles present a higher association between metabolite and predictor. P-values were calculated by linear regression models with the milk compound as independent variable, adjusted for infant sex, and the infant’s age at blood withdrawal. Random intercepts were modelled for batch number and study centre. P-values were corrected (PLME) for multiple testing using Bonferroni’s methods, this is by dividing the p-value with number of metabolites (n = 184).
(TIFF)

S2 Fig **Inter-Correlations between breast milk fatty acids percentages at 1 month after birth (a) 4 months after birth (b)**. Spearman correlation coefficients are plotted for fatty acid-percentage-fatty-acid-percentage correlation.
(TIFF)

S1 Table Associations/Correlations between breast milk macronutrient classes (fat and protein) to infant serum metabolites at 4 months of age. Breast milk components were measured at month 1 or month 4. Spearman correlation (S) was performed to assess correlation. Correlations coefficients (R), P-values (P) and number of observation (N) are presented for S. Associations were calculated by linear regression models (LM) with the milk compound as independent variable, adjusted for infant sex, breastfeeding status at 4 blood withdrawal (exclusively BF yes/no), and the infant’s age at blood withdrawal. Random intercepts were modelled for batch number and study centre. Estimates (B), P-values (P) and number of observation (N) are presented for the LM. P-values of S and LM were further corrected for multiple testing using Bonferroni’s methods (Padj), this is by dividing the p-value with number of metabolites (n = 184).
AA, amino acids; Carn, acylcarnitines; LPC, lysophosphatidylcholines; PC aa, diacyl-phosphatidylcholines; PC ae, acyl-alkyl-phosphatidylcholines SM, sphingomyelins.
(XLSX)

S2 Table Associations/Correlations between breast milk fatty acid percentage to infant serum metabolites at 4 months of age. Breast milk components were measured at month 1 (a) or month 4 (b). Spearman correlation (“Spearman” sheets) was performed to assess correlation. Correlations coefficients (R) and P-values (are presented for S. Associations were calculated by linear regression models (“LME sheets”) with the milk fatty acid percentage as independent variable, adjusted for infant sex, breastfeeding status at 4 blood withdrawal (exclusively BF yes/no), and the infant’s age at blood withdrawal. Random intercepts were modelled for batch number and study centre. Estimates (beta) and P-values (p) are presented for the LME.
AA, amino acids; Carn, acylcarnitines; LPC, lysophosphatidylcholines; PC aa, diacyl-phosphatidylcholines; PC ae, acyl-alkyl-phosphatidylcholines SM, sphingomyelins.
(XLSX)

S3 Table Associations/Correlations between breast milk hormones (adiponectin, IGF-II, insulin and leptin) to infant serum metabolites at 4 months of age. Breast milk components were measured at month 1 or month 4. Spearman correlation (S) was performed to assess correlation. Correlations coefficients (R), P-values (P) and number of observation (N) are presented for S. Associations were calculated by linear regression models (LM) with the milk compound as independent variable, adjusted for infant sex, breastfeeding status at 4 blood withdrawal (exclusively BF yes/no), and the infant’s age at blood withdrawal. Random intercepts were modelled for batch number and study centre. Estimates (B), P-values (P) and number of observation (N) are presented for the LM. P-values of S and LM were further corrected for multiple testing using Bonferroni’s methods (Padj), this is by dividing the p-value with number of metabolites (n = 184).
AA, amino acids; Carn, acylcarnitines; LPC, lysophosphatidylcholines; PC aa, diacyl-phosphatidylcholines; PC ae, acyl-alkyl-phosphatidylcholines SM, sphingomyelins.
(XLSX)

S1 File Protocol of the PreventCD trial as outlined in the 6th framework programme, contract FP6-2005-FOOD-4B-36383–PREVENTCD. (PDF)

S2 File STROBE checklist of the used maternal breast milk and infant serum samples of the PreventCD Trial and the accomplished data analysis in the presented manuscript. (PDF)
