# Supplementary figures and images for: The impact of human breast milk components on the infant metabolism
Source: PLoS One. 2018 Jun 1;13(6):e0197713. doi: 10.1371/journal.pone.0197713 (PMC5983411; doi:10.1371/journal.pone.0197713)

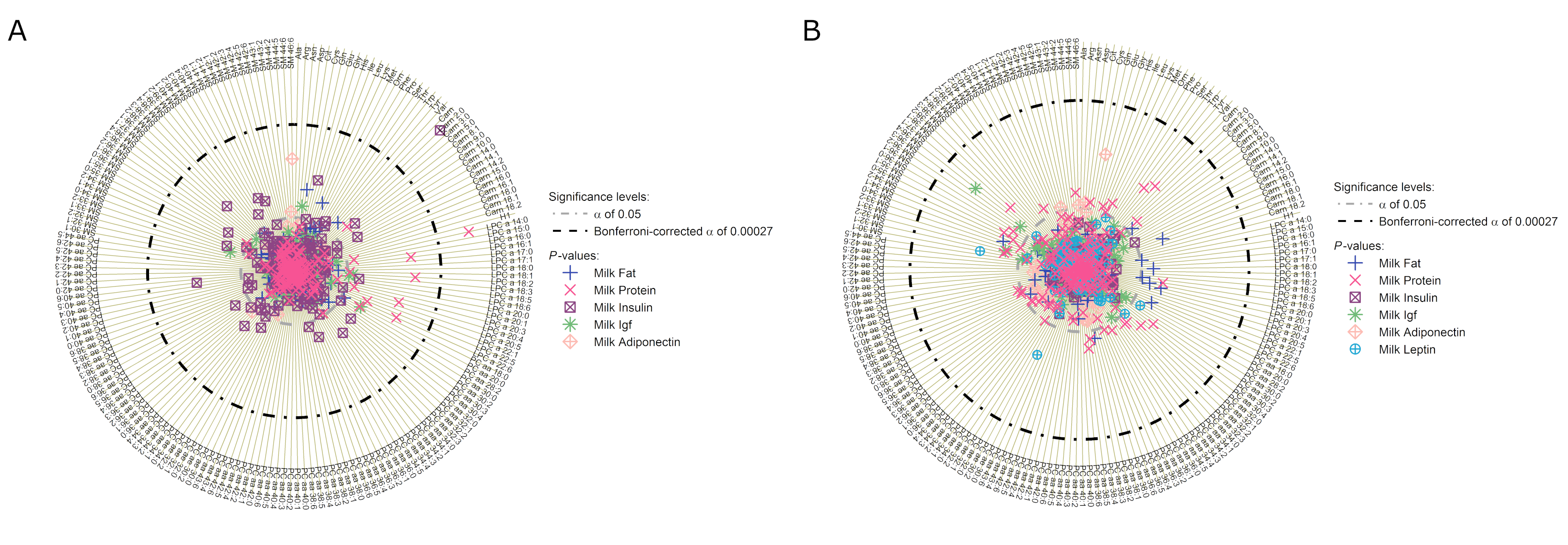

Supplement: S1 Fig — Breast milk components were measured at month 1 (a) or month 4 (b). Negative log-transformed P-values are plotted for each metabolite arranged by metabolite group and species. Higher values represented in the outer circles present a higher association between metabolite and predictor. P-values were calculated by linear regression models with the milk compound as independent variable, adjusted for infant sex, and the infant’s age at blood withdrawal. Random intercepts were modelled for batch number and study centre. P-values were corrected (PLME) for multiple testing using Bonferroni’s methods, this is by dividing the p-value with number of metabolites (n = 184). (TIFF) [file pone.0197713.s001.tiff]
