# Supplementary material for: The impact of human breast milk components on the infant metabolism
Source: PLoS One. 2018 Jun 1;13(6):e0197713. doi: 10.1371/journal.pone.0197713 (PMC5983411; doi:10.1371/journal.pone.0197713)
Supplement: S1 File — (PDF) [file pone.0197713.s006.pdf]

SIXTH FRAMEWORK PROGRAMME
PRIORITY 5
Food Quality and Safety

Contract for:

SPECIFIC TARGETED RESEARCH OR INNOVATION PROJECT

Annex I - "Description of Work"

Project acronym: PREVENTCD
Project full title: **INFLUENCE OF THE DIETARY HISTORY IN THE PREVENTION OF COELIAC DISEASE: POSSIBILITIES OF INDUCTION OF TOLERANCE FOR GLUTEN IN GENETIC PREDISPOSED CHILDREN**

Proposal/Contract no.: **036383**
Related to other Contract no.: *(to be completed by Commission)*

Date of preparation of Annex I: July 20 2010
Operative commencement date of contract: *29 December 2006*

Table of Contents

1. Project summary p. 4

2. Project objective(s) p. 5

3. Participant list p. 14

4. Relevance to the objectives of the specific programme and/or thematic priority p. 15

5. Potential Impact p. 18
- **5.1 Contributions to standards** p. 19
- **5.2 Contribution to policy developments** p. 19
- **5.3 Risk assessment and related communication strategy** p. 21

6. Project management and exploitation/dissemination plans p. 24
- **6.1 Project management** p. 24
- **6.2 Plan for using and disseminating knowledge** p. 29
- **6.3 Raising public participation and awareness** p. 29

7. Workplan– for whole duration of the project
- **7.1 Introduction - general description and milestones** p. 31
- **7.2 Workplanning and timetable** p. 33
- **7.3 Graphical presentation of work packages** p. 34
- **7.4 Work package list /overview** p. 35
- **7.5 Deliverables list** p. 36
- **7.6 Work package descriptions** p. 39

8. Project resources and budget overview
- **8.1 Efforts for the project (STREP/STIP Efforts Form in Appendix 1)** p. 65
- **8.2 Overall budget for the project (Forms A3.1 & A3.2 from CPFs)** p. 67
- **8.3 Management level description of resources and budget** p. 69

9. Ethical issues p. 71
- **9.1 Declaration on the exclusion of particular research activities** p. 71
- **9.2 Conformity with fundamental ethic principles as well as national and EU legislations** p. 72
- **9.3 Description of the sensitive ethical issues of the proposed research** p. 77
- **9.4 Handling of human biological samples and personal data** p. 96
- **9.5 Conflicts of interest** p. 98
- **9.6. Ethical management** p. 98

10. Other issues (optional) p. 100
10.1 Gender dimension p. 100
10.2 List of the members of the CDEUSSA European platform of experts on CD p. 101
10.3 List of Abbreviations p. 104
11. Appendix A. Consortium description p. 106
11.A.1 Participants and consortium p. 106
11.A.2 Sub-contracting p. 136
11.A.3 Third Parties p. 136
11.A.4 Funding of Third country participants p. 136

Fig 1 Management and governance structure of PREVENTCD p. 25
Fig 2 Work planning, showing the timing of the different Work Packages (Gantt chart) p. 33
Fig 3 Graphical presentation of the Work Packages p. 34
Table1 Time scheme of the assessments in the enrolled children until the age of 37 months p. 46
Table2 Members of the coeliac disease patient societies collaborating in the project p. 48
Table3. Participants in the consortium: functions and Complementarity p. 107
Annex1 Approval of the Swedish population based multicenter study by the relevant Swedish Ethical Review Board, Sweden
Annex2 Example of written informed consent form for the family study
Annex3 Example of written informed consent form for DNA research in the family study
Annex4 Folder invited children and parents for the Swedish population based study

1. Project summary

Coeliac disease (CD) is a chronic disorder caused by hypersensitivity to some of the most common proteins (gluten) in the diet of the European population. CD affects as much as 1% of the Europeans (2.5 million people) and is the most common food intolerance in Europe. If recognised, CD patients have only limited access to safe foods and there is not causal therapy available. The general objective of this STREP is to significantly reduce the number of people suffering from CD in Europe, by developing primary prevention strategies for CD**.** By European collaboration of leaders from disciplines beyond those traditionally used a.o. from academia, patient organisation and industry, we will carry out innovative research to examine the hypothesis that it is possible to induce tolerance for gluten in genetically predisposed children through the introduction of small quantities of gluten during the period of breast-feeding. Measurable objectives are: 1) Taking full advantages of genomics techniques: Identification of the immunological mechanisms involved in initiating the aberrant response to gluten introduction in the diet of infants genetically predisposed to CD; 2) Identification of the factors in the early dietary history involved in the aberrant response to gluten in children; 3) Development of new European guidelines for early nutrition in order to prevent CD. To achieve our objectives we propose the following structure and methodology: I) A prospective, randomized, blind dietary intervention study in 1000 young children from high-risk families for CD, to asses the possibilities to induce immune tolerance for gluten in genetically predisposed children; II) A follow-up population study in 16.000 Swedish children aged 12 years, born during and after the Swedish epidemic of CD, to asses the late effect of dietary history, concerning gluten exposure and breast-feeding, on the development of CD and related autoimmune phenomena**.**

2. Project objective(s) Coeliac disease (CD) is a chronic disorder caused by hypersensitivity to some of the most common proteins (i.e. gluten) in the diet of the European population. CD affects as much as 1% of the Europeans.

The general objective of this STREP is to significantly reduce the number of people suffering from CD in Europe, by developing primary prevention strategies for CD. To achieve this objective we propose to study:

1. ***The influence of early dietary history*** *on the prevention and development of CD;*
2. *Its relation to* ***genetic, immunological and environmental factors****;*
3. *The different* ***early feeding practices, including gluten introduction and breast-feeding, in young European children****.*

The proposed project will examine the complex interactions between gluten consumption and other determinants in the development of CD, including:

1. Genetic background;
2. Early immune response to gluten introduction;
3. Early dietary history, including:
    a. Gluten introduction and
    b. Breast-feeding

To identify preventable risk factors and develop prevention strategies for CD in Europe. To achieve this aim, European leaders in basic science on CD will collaborate with their counterparts in clinical and preventive medicine. Together they will examine the hypothesis that it is possible to induce tolerance for gluten in genetically predisposed children through the introduction of small quantities of gluten during the period of breast-feeding.

The measurable objectives within the project are:

1. **Identification of the immunological mechanisms involved in initiating an aberrant response to gluten introduction in the diet of infants genetically predisposed to CD.**

    We will develop a common European databank on such complex interactions as the early gluten intake by young European children from high-risk families regarding CD, their early immune responses to this food antigen, and their genetic backgrounds. Thus, we seek to identify key risk factors in early nutrition with regard to the development of the food hypersensitivity most

common in Europe (i.e. CD). We will carefully assess the early immune response to gluten in 1.000 infants from high-risk families regarding CD from different European countries. We will relate this to the risk determined by the currently accepted genetic risk factors (HLA-DQ, CTLA4, the CELIAC2 locus on 5q, and gene X) under different nutritional conditions, including early gluten introduction and breast-feeding.

2. **Identification of the factors in the early dietary history involved in the aberrant response to gluten in children.**
   We will assess for CD 16.000 children aged 12, who were born during the Swedish CD epidemic (8.000 children born in 1993) and during the post-epidemic period (8.000 children born in 1997), respectively. The differences between both groups with respect to gluten exposure during infancy have been suggested to influence the risk for CD.
   This population study in two large, well documented European cohorts of children, will offer the opportunity to identify the late effects of early dietary history with respect to gluten exposure and breast-feeding, involved in triggering the abnormal response to gluten and to another auto-antigens in the general population.
3. **Development of new European guidelines for early nutrition in order to prevent CD in genetically predisposed young children.**
   Our hypothesis is that it is possible to induce tolerance for gluten in children by gradually introducing small quantities of gluten during the period of breast-feeding. If this hypothesis is proven to be correct, we will develop new European guidelines for early nutrition with regards to gluten introduction and breast-feeding. Thus, we aim to achieving primary CD prevention in the general population and, in particular, in children from high-risk families for CD. The new European guidelines will be developed and disseminated in collaboration with the Committee of Nutrition of the European Society for Paediatric Gastroenterology, Hepatology and Nutrition (ESPGHAN).

2.1 State of the art

Coeliac disease (CD) is a chronic disorder of the small intestine, resulting from aberrant cellular responses to gluten peptides, affecting as much as 1% of the

European population (1, 2). In susceptible individuals, ingestion of gluten (i.e. proline and glutamine rich proteins) triggers an aberrant immune response which results, by means of a cascade of events, in chronic enteropathy (3-6). The aetiology of CD is complex and not yet fully understood. CD has a strong genetic component: 95% of the patients carry the HLA-DQ2 (A1*0501-B1*0201) genes and 3% the HLA-DQ8 (A1*0301-B1*0302) genes (7). Outside the HLA region there are at least 3 genomic areas related to CD: CELIAC 2 on 5q31-33, CELIAC3 on 2q33 and CELIAC4 on 19p13 (8). From two of these regions the responsible genes have been identified: CTLA4 on 2q (9) and gene X on 19p (10). The significant contribution of genes to the disease is also evident from the increased familial clustering: first-degree relatives of CD patients have an increased risk of 5-10% of developing the disease (11).

CD is a frequent disease. Screening studies have shown that CD is severely under-diagnosed, with a prevalence of 0.5-1% among the European population (12). CD is frequently unrecognised by physicians, among others because of its variable clinical presentation and symptoms (13). Assuming a conservative prevalence of 0.5% this corresponds to about 2.5 million CD cases in Europe. Approximately 85% of these cases are unrecognised and thus also untreated. The health burden of CD is considerable: reducing the quality of life both for the affected subjects and for their families (14). On a societal level it has extensive negative economical consequences. CD subjects also have an increased risk for other autoimmune diseases (20%); a risk suggested to increase with duration of gluten exposure. Moreover, a gluten-dependent occurrence of diabetes and thyroid related antibodies have been demonstrated (16). Similarly, some 5% of patients with other autoimmune diseases, i.e. type 1 diabetes and thyroid disease, suffer from CD (1). Moreover, a gluten-dependent occurrence of diabetes and thyroid related antibodies has been demonstrated, with unknown clinical impact (17, 18). An important American study has beeb published on a cohort of 1560 children who had an increased risk of developing CD or type1 diabetes, as defined by possessing either HLA-DR3 or DR4 alleles, or having a first-degree relative with type 1 diabetes, derived from the DAISY project (Diabetes Autoimmunity Study in the Young). At a mean follow-up of 4.8 years, the authors concluded that: 1) there is a “window of opportunity” of introducing gluten into the diet when the child is aged between 4 and 6 months with regard to the risk of developing CD, and 2) that the contribution of breast-feeding was to be disregarded in this respect (19). However, the authors did not make specific attempts to calculate the

gluten amount ingested by the children or to correlate this important early nutrition event with the presence or absence of breast-feeding.

A systematic review and a meta-analysis of observational studies published between 1966 and June 2004 that examined the association between breast feeding and the development of CD has been published (20), showing that breast feeding during the introduction of dietary gluten, and increased duration of breast feeding were associated with reduced risk of developing CD. Additional studies, like the one proposed here by us, may shed light on the importance of the quantity of exposure to gluten in early life in the development of CD (21). One problem in this respect is that, until now, there were no validated instruments to quantify gluten intake by young infants. The instruments available were work intensive and time consuming and difficult to use in population studies. Recently the Dutch partner has developed and validated food frequency questionnaires (FFQ's) for this propose, using the 2-day food record as a reference (22). In the proposed STREP we will use these FFQ's to calculate the gluten amount ingested by the infants.

CD has a multifactorial aetiology, resulting from an interaction between a strong genetic basis, immunological and environmental factors (2). During recent years an impressive amount of research work on CD has been produced by different European groups, among others by those collaborating in FP5 and FP6 projects. Thus many immunological, genetic, epidemiological, environmental and socio-economic factors implicated in CD have been clarified (23). However, there is a fundamental gap between the groups of researchers working on the basic scientific aspects of CD (immunology, genetics, cereal chemistry) and those working on the clinical and social aspects of the disease (epidemiology, preventive and curative medicine). That is possibly the reason why the only treatment currently available for CD is exactly the same as the one discovered more than 50 years ago: a life-long gluten-free diet. This diet is very difficult to follow, because gluten-containing foods are widespread in the European daily diet. In addition, a gluten-free diet is expensive, given an extra cost of € 1,200-1,300 per patient and year, according to an estimate by the Dutch Association of Coeliac Patients (NCV). *This corresponds to a total European financial burden of € 3.0-3.3 billion, when considering all 2.5 million CD cases.* When CD cases are adequately diagnosed and treated, this extra cost is compensated by an increase in working time and reduced health care costs, beside the important increase in life

quality gained by the affected person. However, primary prevention would be even more beneficial both for economic and humanitarian reasons.

Epidemiological studies from Sweden suggest that early nutrition patterns may have a significant impact on the later risk of developing CD. The occurrence of "epidemics" of CD after changes in the Swedish infant feeding during the 1980s and 1990s, suggests that the disease may be preventable by improving early nutrition and inducing tolerance to gluten in predisposed individuals. The leader of Swedish research, A. Ivarsson, who is a partner of the proposed STREP, has shown that ongoing breast-feeding during the period of gradual introduction of gluten containing foods into the infant diet reduces the risk of symptomatic CD (24,25). Based on an estimate of the attributable fraction, *half of the CD cases during the Swedish epidemic might have been avoided* if all infants had been introduced to gluten in small amounts while still being breast-fed. The latter finding opens the way to possible prevention strategies, but they are in conflict with the actual European guide lines for infant nutrition. This advice is to introduce gluten into the diet no earlier than at the age of 6 months (26). At this age, only a low percentage of the European children, ranging from 1-46% in the different European countries, receive breast-feeding (27). On the other hand, the Swedish study only investigated the effect of the early dietary history on symptomatic CD in children aged 2. It may be that the nutritional factors only have an effect on the symptoms of CD and/or on the time of presentations of the symptoms. In addition, there is no information on the biological mechanisms involved in the effect of early nutrition in the development of CD.

Basic science, including genetics, immunology and genomics, should find its way into the clinical application in the prevention of CD. *The objective of this STREP is to close the gap between basic science knowledge, concerning genomics and immunology, and its applications in the prevention of CD, concerning nutrition history, medicine, Public Health and food industry.*

Through European collaboration of leaders from disciplines beyond those traditionally used, among others from academia, patient organisations and industry, we will carry out innovative research on primary prevention of CD by studying the effect of the dietary history in the induction of early and later (in)tolerance for gluten and other auto-immune phenomena. Taking full advantage of genomics techniques we will elucidate (some of) the genetic and immunological mechanisms involved in this process.

The proposed project will help to identify vulnerable population groups, genetically predisposed to CD, and to assess how improved food and early nutrition guidelines might reduce the prevalence of CD in the future. A better understanding of the influence of the dietary history on human health will establish the basis to provide safer, and health-promoting early nutrition guidelines so as to improve the health and well-being of the European citizens.

The proposed project represents a significant step forward in the integration of knowledge from basic science into the prevention and treatment of food-related diseases in Europe, in this case CD.

REFERENCES

1. Green PHR, Jabri B. Coeliac disease. Lancet 2003; 362:383-91.
2. Koning F, Schuppan D, Cerf-Bensussan N, Sollid LM. Pathomechanisms in celiac disease. Best Pract Res Clin Gastroenterol 2005;19: 373-87.
3. Wal van de Y, Kooy Y, Veelen van P, Pena S, Mearin L, Molberg Q, Lundin L, Mutis T, Benckhuijsen W, Drijfhout J.W, Koning F. Small intestinal cells of celiac disease patients recognize a natural pepsin fragment of gliadin. Proc. Natl. Acad. Sci. USA 1998; 95: 10050-54.
4. Vader W, Kooy Y, van Veelen, P, de Ru A, Harris D, Benckhuijsen W, Pena S, Mearin L, Drijfhout JW, Koning F. The gluten response in children with recent onset celiac disease. A highly diverse response towards multiple gliadin and glutenin derived peptides. Gastroenterology 2002, 122:1729-37.
5. Vader W, de Ru A, van der Wal Y, Kooy Y, Benckhuijsen W, Mearin L, Drijfhout JW, van Veelen P, Koning F. Specificity of tissue transglutaminase explains cereal toxicity in celiac disease. J. Exp. Med 2002; 195: 643-9.
6. Spaenij-Dekking EHA, Kooy-Winkelaar EMC, Drijfhout JW, Koning F . A novel and sensitive method for the detection of T cell stimulatory epitopes of α/β- and γ- gliadin. GUT 2004; 53: 1267-73
7. Sollid LM, Markussen G, Ek J, Gjerde H, Vartdal F, Thorsby E. Evidence for a primary association of celiac disease to a particular HLA-DQ alpha/beta heterodymer. J Exp Med 1989; 169:345-50.
8. van Heel DA, Hunt K, Greco L, Wijmenga C. Genetics in coeliac disease. Best Pract Res Clin Gastroenterol 2005; 19: 323-39.
9. van Belzen MJ, Mulder C JJ, Zhernakova A, Pearson PL, Houwen RHJ, Wijmenga C. *CTLA4*+49 A/G and CT60 polymorphisms in Dutch coeliac disease patients. Eur J Hum Genet 2004;12:782-5.
10. Monsuur AJ, de Bakker PIW, Alizadeh BZ, Bevova MR, Franke L, van t'Slot R, van Belzen MJ, Diosdado B, Zhernakova A, Daly MJ, Mulder CJJ, Mearin ML, Meijer JWR, Meijer GA, van Oort E, Wapenaar MC, Koeleman BPC, Wijmenga C. Myosin IXB variant increases the risk of celiac disease and points toward a primary intestinal barrier defect. Nat Genet. 2005;37:1341-4.
11. Babron MC, Nilsson S, Adamovic S, Naluai AT, Wahlstrom J, Ascher H, Ciclitira PJ, Sollid LM, Partanen J, Greco L, Clerget-Darpoux F. European

Genetics Cluster on Coeliac Disease. Meta and pooled analysis of European coeliac disease data. Eur J Hum Genet 2003;11: 828-34.

12. Mearin ML, Ivarsson A, Dickey W. Coeliac disease: is it time for mass screening? Best Pract Res Clin Gastroenterol 2005; 19:441-52.
13. Steens RFR, Csizmadia CGDS, George EK, Ninaber MK, Hira Sing RA, Mearin ML. Better recognition of childhood celiac disease in the Netherlands and its changing clinical picture: a national prospective study 1993-2000. J Pediatr 2005; 147: 239-43.
14. Kolsteren MMP, Koopman HM, Schalekamp G, Mearin ML. Health-related quality of life of childen with celiac disease. J Pediat 2001;138:593-5.
15. Spaenij-Dekking L, Kooy-Winkelaar Y, van Veelen P, Wouter Drijfhout J, Jonker H, van Soest L, Smulders MJ, Bosch D, Gilissen LJ, Koning F. Natural variation in toxicity of wheat: potential for selection of nontoxic varieties for celiac disease patients. Gastroenterology 2005; 129: 797-806.
16. Ventura A, Magazzu G, Greco L. Duration of exposure to gluten and risk for autoimmune disorders in patients with celiac disease. Gastroenterology 1999;117:297-303.
17. Norris J, Barriga K, Klingensmith G, Hoffman M, Eisenbarth G, Erlich H, et al. Timing of initial cereal exposure in infancy and risk of islet autoimmunity. JAMA 2003; 290: 1713-20.
18. Ziegler A, Schmid S, Huber D, Hummel M, Bonifacio E. Early infant feeding and risk of developing type 1 diabetes-associated autoantibodies. JAMA 2003; 290: 1721-8.
19. Norris JM, Barriga K, Hoffenberg EJ, Taki I, Miao D, Haas JE, Emery LM, Sokol RJ, Erlich HA, Eisenbarth GS, Rewers M. Risk of celiac disease autoimmunity and timing of gluten introduction in the diet of infants at increased risk of disease. JAMA 2005; 293: 2343-51.
20. Akobeng AK, Ramanan AV, Buchan I, Heller RF. Effect of breast feeding on risk of coeliac disease: a systematic review and meta-analysis of observational studies. Arch Dis Child 2006; 91: 39-43.
21. Farrel RJ. Infant gluten and celiac disease: too early, too late, too much, too many questions. JAMA 2005; 293: 2410-2.
22. Kiefte-de Jong JC, Hopman GD, Cessie le S, Bleeker SE, Moll HA, Mearin ML. Gluten introduction and breast-feeding in Dutch infants. In preparation.

23. National Institutes of Health Consensus Development Conference Statement on Celiac Disease, June 28-30, 2004. Gastroenterology 2005; 128:S1-9.
24. Ivarsson A, Hernell O, Stenlund H, Persson LÅ. Breast-feeding protects against celiac disease. Am J Clin Nutr 2002;75: 914-21.
25. Carlsson A, Agardh D, Axelsson IEM, Borulf SK, Grodzinsky, Ivarsson SA. Prevalence of celiac disease: Before and after a national change in feeding recommendation. Scand J Gastroenterol. In press.
26. WHO, The optimal duration of exclusive breastfeeding. Systematic Review, March 2001, Geneva, 28-30.
27. Cattaneo A, Yngve A, Koletzko B, Guzman LR; Promotion of Breastfeeding in Europe project. Protection, promotion and support of breast-feeding in Europe: current situation. Public Health Nutrition 2005, 8: 39-46.

3. Participant list

List of Participants

Partic. Role*	Partic. no.	Participant name	Participant short name	Country	Date enter project**	Date exit project**
CO	1.	Academisch Ziekenhuis Leiden *acting under the name* Leids Universitair Medisch Centrum	LUMC	Netherlands	Month 1	Month 48
Partner	2.	University of Naples, Federico II	UNINA	Italy	Month 1	Month 48
Partner	3.	Umeå University	UMU	Sweden	Month 1	Month 48
Partner	4.	Akademia Medyczna w Warszawie	WMU	Poland	Month 1	Month 48
Partner****	5.	University of Medical Center Utrecht	UMCU	Netherlands		
Partner***	5.	University Medical Center Groningen	UMCG	Netherlands	Month 1	Month 48
Partner	6.	La Paz University Hospital	HULP	Spain	Month 1	Month 48
Partner	7.	La Fe University Hospital	HULF	Spain	Month 1	Month 48
Partner	8.	Danone Research BV	Danone	Netherlands	Month 1	Month 48
Partner	9.	Association of European Coeliac Disease Societies	AOECS	Belgium	Month 1	Month 48
Partner	10.	Phadia GmbH	Phadia	Germany	Month 1	Month 48
Partner	11.	Eurospital SpA	Eurospital	Italy	Month 1	Month 48
Partner	12.	TECHNION – Israel institute of Technology	TECHNION	Israel	Month 1	Month 48
Partner	13.	University of Oslo	UO	Norway	Month 1	Month 48
Partner	14.	Children's Hospital Zagreb	CHZ	Croatia	Month 1	Month 48
Partner***	15.	Heim Pal Children's Hospital	HP	Hungary	Month 2	Month 48
Partner***	16.	Universitat Rovira i Virgili, Hospital Universitari de Sant Joan de Reus	URV	Spain	Month 2	Month 48
Partner***	17.	Ludwig Maximilians University<br>Third party: Dr. v. Haunersches Kinderspital (third party)	LMU	Germany	Month 3	Month 48

* CO = Coordinator
CR = Contractor
** Normally insert "month 1 (start of project)" and "month n (end of project)"
These columns are needed for possible later contract revisions caused by joining/leaving participants

*** Partners will accede to the contract in accordance with the 1st contract amendment

**** Partner removed from consortium due to its nonaccession to the contract

4. Relevance to the objectives of the specific programme and/or thematic priority

The proposed project contributes to the objectives of the 6th Framework Programme, priority "Food Quality and Safety", and its scientific objectives strictly comply with those of the area "Epidemiology of food-related diseases and allergies". Coeliac disease (CD) is the most common food intolerance, its prevalence being approximately 1:100 in the European population. **If recognised, CD patients have only limited access to safe foods and there is no causal therapy available.** This project is expected to add significantly to the present knowledge on the possibility of identifying vulnerable individuals within the population; it will provide information concerning the natural history of this condition and about the possible protective role of specific early dietary regimens. Moreover, the influence of genetic variability will be studied, linking it to the measurement of the immune response to gluten.

Influence of early nutrition in relation to genetic factors

The main objective will be to study the influence of dietary history, including early nutrition, on the development of CD in relation to genetic factors. These aims will be accomplished both at general population level and in a selected "high-risk" group. In the first case, the long-term influence of dietary regimes in the first year of life on the development of CD will be investigated, taking advantage of the epidemic of CD experienced in Sweden in the mid-80s. Potential contributing environmental risk factors were identified, as it was found that the risk of developing CD was possibly related to the dietary history and reduced when gluten-containing foods were introduced into the diet while the infant was still being breast-fed. Now, we wish to determine the long-term effects of these different dietary regimens. In the second arm of this part of the project, these conditions will be reproduced in a prospective randomised controlled study, through an active intervention based on the administration of small amounts of gluten in a period when the infant is still being breast-fed. This second part of the study will be realised in subjects genetically at risk; i.e. first-degree relatives of celiac patients. It is expected that the potential results of this effort will go well beyond the limit of the project. This cohort could be followed for a longer period of time, adding further relevant information on the natural history of this condition and on its relation with the genetic make-up of the individual. In fact, from the genetic point of view, this project will contribute to verifying prospectively

whether the genetic factors identified already are really associated with the risk of developing CD and, if so, to what extent.

Identification of molecules involved in the expression of the disease and genes they are linked to.

This project aims to elucidate the role of HLA and non-HLA genes in relation to early feeding in the development of CD. It will take advantage of the knowledge available so far (data obtained mostly by the same researchers involved in this project). Importantly, the information, here validated in a prospective study, is expected to reinforce the role attributed to HLA and non HLA genes The identification of molecules coded by the latter genes, in some cases in a very advanced phase, will significantly contribute to the better comprehension of the pathogenesis of the disease.

Early nutrition and autoimmunity

The main outcome evaluated in this project is the development of CD. This condition is strictly associated with autoimmune conditions and it is considered to be autoimmune in nature. Both the follow-up of the Swedish cohorts and of the newborns from the intervention study in high-risk families may provide important information on the relation between the early dietary history and the development of autoimmune diseases in general. Again, this is very relevant to understand the relation between early nutrition and health in the remaining lifetime of the individuals. As a whole, the project will provide new knowledge about the amount and the modalities of the introduction of gluten in the diet of the infant in its first year of life. As a result of that, new regulations could be implemented at the European level, based on scientific evidence.

Relevance to the wider societal and policy objectives of the 6th FP.

All aspects of the proposed project relate to society and comply with the wider societal and policy objectives of the 6th FP. Multifactorial diseases and the relationship between food and health are major issues in Europe, as well as the improvement of the health and well-being of the European citizens through improved food habits during infancy and early childhood. Through the participation of the European patient organisations and the integration of societal research into the project, the results will become directly available for implementation. In fact, the

Association of European Coeliac Disease Societies (AOCS) is a full partner in the project and will collaborate on the management of the ethical issues and socio-economic and public health perspectives. The possibility of identifying those predisposed to the disease and of implementing prevention strategies could have heavy implications, both socially and for public health, with regard to the direct and indirect cost of the disease. The mandatory dietary regimen is supported by most European Countries and sums up to several hundreds of million Euros at the EC level. Moreover, the actual diagnostic procedure requires invasive and expensive procedures (endoscopy and small bowel biopsies). Hence, the patients association wishes the development of diagnostic tools based on molecular methods in order to minimise the requirement for invasive tests. The development of genetic markers will increase the post test likelihood of predicting the disease, and the combination with highly sensitive and specific tools might eventually build up an alternative diagnostic tool. Finally, this project is very much coherent with the activities promoted by the Specific Support Action on CD, recently approved by the European Commission: CDEUSSA (Food – CT- 2005- 517787). This project aims to establish a European platform on the prevention of CD, integrating basic scientific knowledge in clinical applications and activities of the food industry. The proposed project establishes an active European research network where basic scientists (geneticists and immunologists), epidemiologists, clinicians, patient organisations and food and diagnostics firms are represented and could be considered as the first important result of the new European platform.

5. Potential Impact

Screening studies for coeliac disease (CD) in Europe have revealed prevalence as high as 1.0% and in first-degree relatives of CD patients the frequency of the disease is as common as 10%. Thus, CD has emerged as a *new European public health problem*. A conservative prevalence of 0.5% corresponds to about 2.5 million CD cases in Europe, out of which about 85% are unrecognised and thus also untreated. In a life perspective *the negative health consequences of untreated CD are extensive* and considerably reducing the quality of life. The necessary gluten free diet gives an extra cost of € 1,200-1,300 per patient a year, according to an estimate by the Dutch Association of Coeliac Patients (NCV). This corresponds to a total European financial burden of €3.0-3.3 billion, when considering all 2.5 million CD cases. Thus, *the negative economical consequences are substantial,* with additional expenditure due also to lost working-time and misspent healthcare cost.

Solving society problems

CD has, until recently, been considered as unavoidable. However, this view was recently challenged by an epidemic of CD among children in the genetically stable Swedish population. Such an epidemic curve of CD incidence indicates an abrupt change in causal factors. Further studies suggest that half of the epidemic might have been prevented if all infants had been introduced to gluten-containing foods gradually and preferably while still breast-fed. **If so, it might be that CD could be prevented in millions of Europeans by changes in infant diet practices.** *Thus, primary prevention might be an option for CD, and this is highly preferable for both humanitarian and economical reasons.*

By European collaboration among leaders from academia, patient organisations and industry, **the PREVENTCD project will carry out *innovative research on primary prevention of CD by studying the effect of the early dietary history in the induction of (in)tolerance for gluten in genetic predisposed children*.** The proposed project represents a significant step forward in the integration of knowledge from basic science into the prevention and treatment of food-related diseases in Europe, in this case CD.

Innovative impact

The results of PREVENTCD may have an innovative impact in the European Food Industry, concerning the scientific basis for the composition of bottle-feeding, aimed

to prevent CD in children in whom breast-feeding is not possible. In this context, the studies on gluten content in breast-feeding samples from mothers from high risk families from different European countries with different nutritional practices, will offer novel data to the European Industry in its effort to develop food for health. Morover, the *commercial diagnostic tools* used in PREVENTCD, i.e. serological markers for CD (AtTG, AGA) and autoimmunity (TPO), and also for determining the genetic vulnerabilty (HLA-typing) for CD, will have a *marketing advantage* as being used in high quality international research.

5.1 Contributions to standards

European and national guidelines for gluten introduction into the diet of infants have been changed several times over the years, and in retrospect the scientific evidence behind some of these changes can be questioned. In fact, the European infants of today get an introduction to gluten containing foods following guidelines that, for the most part, are not based on scientific evidence and the option for primary prevention of CD is not properly taken care off. The large majority of the European infants get relatively big quantities of gluten introduced at once after the age of 6 months, a moment when very few of them are still breast-fed. Thus, ***new European guidelines for infant feeding with respect to introduction of gluten-containing foods are needed***, and should be based on high quality scientific evidence. The PREVENTCD project will provide such evidence through *multidisciplinary research collaboration across Europe*. A better understanding of the influence of the dietary history on human health will establish the basis to provide safer, and health-promoting early nutrition guidelines to improve the health and well being of the European citizens. If the PREVENTCD hypothesis concerning the possibility of induction of tolerance for gluten in children is proven to be correct, we will develop new *European guidelines of early nutrition, concerning gluten introduction and breast-feeding*, to achieve primary CD prevention in the general population and, particular, in children from high-risk families for CD. The new European guidelines will be developed and disseminated in collaboration with the Committee of Nutrition of the European Society for Paediatric Gastroenterology, Hepatology and Nutrition (ESPGHAN).

5.2 Contribution to policy developments

In Europe, CD has been on the scientific agenda since the 1950's, however, increasingly so during the last twenty years. In comparison, in U.S and globally CD

has only recently been recognized as a frequent health problem. By PREVENTCD the *European researchers* within their respective field, and the industry involved, *will keep and strengthen the leading role of Europe in the frontline of CD research worldwide.* Success within this research area can only be reached by an *integrated European research effort*, taking advantage of all the currently available European knowledge and know-how with the large range of disciplines involved. Moreover the participation of the large number of families with CD necessary to carry out the study is only possible by collaboration among European countries with well-organized Coeliac Patients Societies. In this context the participation of the Association of European Coeliac Disease Societies (AOECS) is highly valuable. In addition, the "experiment in nature" that has taken place in Sweden during and after national changes in early gluten consumption, offers a unique opportunity to study the effect of these changes in the primary prevention of CD in one big population of European pre-adolescents.

The PREVENTCD partners are well established in the scientific community within their own countries, and also have international networks across Europe as well as globally. The Dutch partnership includes leaders of the Dutch Coeliac Disease Consortium (CDC), a project supported by the Dutch Government, which brings together partners from industry, patient community and Dutch knowledge-based institutions, to develop the scientific bases for safer foods and for effective diagnosis, prevention and therapy of CD. The coordinator of PREVENTCD, ML Mearin, and several of the partners (A Ivarsson, R Trocone, and H Szajewksa) are responsible for the EU funded project CDEUSSA (Food – CT- 2005- 517787) aiming, a.o., to create a *European platform for future research on prevention and effective treatment of CD by a multidisciplinary approach to integrate basic scientific knowledge in clinical applications and food industry*. This European platform offers an excellent opportunity to bring new knowledge into the project and will act as its Advisory Board.

5.3 Risk assessment and related communication strategy

WP1: the management and governance structure of PREVENTCD is described in detail, including the risks and contingency plans, under "6.1 Project management".

WP2: no contingencies or risks are expected.

WP3: the main activity in WP3 is represented by the recruitment of newborns belonging to at-risk families for CD. The number of newborns expected from each participating group has been carefully evaluated on the basis of the number of families followed-up in that group and of the birth rate of the local general population. Deliverables set at the end of each year of the project will allow following strictly the progression of the recruitment. Already at month 12 it will be possible to have a first evaluation of the recruitment. If at this moment it turns that the recruitment of newborns is significantly lower than expected, all efforts will be done to increase the recruitment rate. This will mainly be done by increasing the awareness about the project by, among others, the members of the local Coeliac Patients Associations. Reinforcement of the call to participate may be done by additional information on the Journals and web-sites of the AOECS and local associations, as well as on the local newspapers and radio and television programmes. Those strategies have previously proved to be successful in increasing the participating rate by target groups in research projects.

Although the project clearly states that breast feeding will be strongly encouraged for all the participating children, and it is expected that families with coeliac cases will strictly comply with this prescription, it is possible that a proportion of infants at the age of 4 months will turn out not to be breast fed. To avoid bias they will be in any case enrolled. Retrospectively, the impact of the length of breast feeding on the outcome of the intervention will be evaluated. It must be emphasised that no evident risk are foreseen for deliverable 3.1, which may be considered as one of the most important deliverables of the whole project. This project is the first large prospective study that will describe the natural history and the development of the specific immune response of subjects belonging to “at-risk” families, in relation to a very precisely defined genetic background. It is also important to stress that the work planned in two other important WPs (genetics and immunology) although certainly done on the infants recruited in WP3 and WP4, are not strictly dependent on the deliverables 3.4, 3.5 and 3.6.

WP4: the population based study doesn't involve any intervention with respect to infant feeding, but takes advantage of a previous "experiment" in Sweden with extensive changes with respect to breast-feeding and early gluten exposure. Thus, for WP 4 risks no contingencies or risks are expected.

WP5: this part of the project relies on existing knowledge on the genetics of CD and the technology available for high-throughput genotyping. This implies that the feasibility is good. The genotyping will be performed on an Illumina platform which is an extremely powerful technology currently in use by numerous groups and also fully operational the UMCG. No contingencies or risks are expected to execute this WP.

WP6:

Gluten analysis

Characterization of the gluten preparation will be done with T cell reagents already established by partners 1 and 13 and with monoclonal antibodies established by partner 1. These tests have already proved to yield reliable results. We therefore see no risks associated with this part of the work package.

Breast milk analysis

For the breast milk analysis we will primarily use the monoclonal antibody based assays. In a pilot study performed in the Netherlands our preliminary findings indicate that gliadin fragments can be reliably detected in the breast milk samples and that these levels remain stable over extended periods of breast milk feeding. We therefore see no risks associated with this part of the work package.

Detection of gluten specific T cells HLA tetramers during gluten exposure.

Detection of gluten specific T cells in peripheral blood by HLA tetramers has so far been successfully undertaken in adult coeliacs in remission on day 6 after a 3 day oral gluten challenge. Detection of HLA tetramer positive T cells has so far not been tested in children and it is unclear whether gluten reactive T cells can be detected in peripheral blood when CD is about to precipitate. The amount of blood needed to detect tetramer positive T cells in children is also uncertain because there is no data

available on the frequency of gluten reactive T cells in peripheral blood under these conditions. Initially we will therefore test 10 patients for tetramer staining. If no positive results emerge, we will terminate this approach and explore alternatives, in particular ELIspot analysis of peripheral blood samples or CFSE dilution experiments in which responding T cells are visualized by the CFSE dilution as the results of cell division. Again we will test 10 patients in each group. If all approaches fail, this part of the workpackage will be terminated.

Repertoire analysis

Intestinal biopsies taken from children at partner site 1 will require no transport and can be analyzed directly. Biopsies taken at sites different from that of partner 1, will require transportation before the culturing of gluten reactive T cells. It is uncertain how well biopsies will maintain their ability to produce functional T cell reagents. This will be tested in simulation experiments before large scale transfer of biopsies will take place. In these simulation experiments we will define the best conditions for transport. If transportation of biopsies does affect T cell outgrowth, we will develop alternative protocols through which the start of the biopsy cultures can be initiated at sites other than partner 1 and 13. Such sites could include partners 2, 4, 6 and 7. After the initial outgrowth of the T cells these could be frozen and shipped to partners 1 and 13 for further analysis.

Regulatory T cell compartment

There is now a large body of literature of markers though which regulatory T cell can be identified through FACS-analysis. Markers include Fox-p3, CTLA-4, CD4CD25 and characteristic cytokine secretion profiles. The techniques for this analysis are all well established. We therefore see no risks associated with this part of the work package.

<u>WP7</u>: no contingencies or risks are expected in this WP.

6. Project management and exploitation/dissemination plans

The management and governance structure of PREVENTCD ensures:

- Effective and optimised management;
- Effective and optimised decision making;
- Enhanced networking between the participants and stakeholders (like patient organisations, public health authorities, research initiatives on coeliac disease (CD)).

The public participation and awareness of the project will be raised by widely dissemination of its activities and results to other directly interested parts, such as food industry, CD patients and patients associations.

The PREVENTCD project will have a *website* and a *newsletter* both to facilitae internal communication, and to make the process and results easily accessible externally. Once strategies for primary prevention of CD have been identified the new European platform on CD formed by EU funded project CDEUSSA (Food – CT-2005- 517787) will contribute to the promptly *dissemination of the new findings to reach and be of benefit to the European citizens health and well-being.* The results will be reported at *national and international scientific meetings*, and published in *national and high impact international journals*. The European citizen, the CD patients and their families, the CD patients Societies, the Food Industry and other relevant groups will be reached by the knowledge gained through workshops and by information on flyers and on-line publications.

6.1 Project management

PREVENTCD is an organization of 14 research, health care, industry and patients' associations with a vested interest in life sciences research, especially in the field of CD. The relationships between the 14 partners in PREVENTCD and their rights and obligations with respect to each other and the project will be regulated in the **Consortium Agreement** for the project according to the STREP requirements. These agreements will be signed before the actual start of the project. The management and governance structure are shown in figure 1.

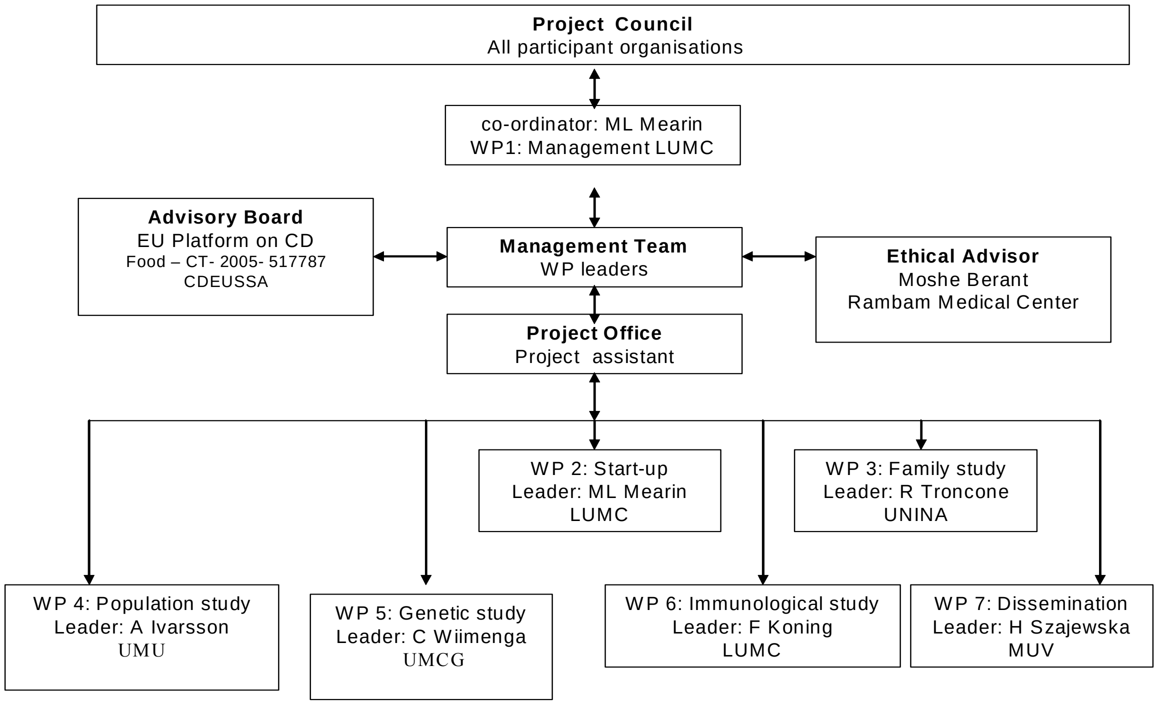

Figure 1. Management and governance structure of PREVENTCD

The **coordinator, Dr. ML Mearin**, of the Leiden University Medical Center (LUMC), acts as the coordinator of PREVENTCD and is responsible for the project reporting and accounting. The duties of the coordinator are described in the Consortium Agreement. They include administrative and financial management, legal and contractual issues, organization of meetings, creating and maintaining the website, and functioning as the central contact and communication point for the European Commission. A **Project Assistant** will support the co-ordinator in her tasks. She/he will be responsible for setting up the **Project Office**, for management, financial accounting and organizational aspects, and for maintaining the web-based activities and the networking activities. They will receive assistance within the LUMC from a financial project officer, an officer of European research grants, from IPR and legal experts, and from ICT specialists and communication experts.

The coordinator will ensure that reports are prepared and submitted to the EC project officer in time and according to instructions (i.e. periodic- mid-term- and final reports, including cost statements and audit certificates).

The **Project Council**, which is composed by the representatives from all the collaborating organisations and is chaired by the co-ordinator, is responsible among others for:

1. Deciding upon the allocation of the project's budget in accordance with the EU Contract, and reviewing and proposing budget re-allocations;
2. Deciding on the plan for using and disseminating knowledge arising from the project.

The major decision making body in the PREVENTCD project is the **Management Team,** which is composed by the leaders of the 7 Work Packages and is chaired by the coordinator. The PREVENTCD **Management Team** will be responsible for the day-to day direction of the project and will be responsible, among others for:

1. Deciding upon changes to the roadmap of activities necessary to achieve the objectives of the project;
2. Deciding upon measures in the framework of controls and audit procedures to ensure effective day-to day coordination and monitoring of the work affecting the project.

The PREVENTCD **Management Team** will meet quarterly. The coordinator and the Management Team together constitute the PREVENTCD management. In order to assist in the management tasks, the website of PREVENTCD will contain a Project Management System.

An **Advisory Board** will be formed by the experts of the European platform on CD, created by the EU SSA project CDEUSSA (Food – CT- 2005- 517787). A list with the members of the CDEUSSA European platform of experts on CD is included in annex 10.2. The aim of this platform of experts is to close the gap between basic science on CD and applications of its results in prevention, clinical treatment and in the food industry. The CDEUSSA platform on CD is formed through European collaboration among leaders from disciplines beyond those traditionally used; among others from academia, patients' associations and industry, so, as to generate a

scientific basis for innovative research on primary prevention and effective treatment of CD. Members of the Management Team will present the results of the project during the platform meetings and will receive advice from the platform on scientific and industrial aspects and with regard to the patients' interest.

Ethical management

Because of the potential ethical aspects of the project the management structure will include as ethical advisor Professor Moshe Berant, chairman of the Helsinki Committee at Rambam Medical Center and member of the Steering Committee of the Israeli Ministry of Health for Regulatory Issues and Monitoring of Clinical Trials in Human Subjects (annex 10.3 CV). The ethical advisor will give advise, asked and unasked, to the Management team of the project with respect to the ethical questions and decisions. See Ethical review PREVENTCD (annex 10.4)**.**

Planning and Control

PREVENTCD is a project in which decisions on activities to be undertaken will be made centrally. On the other hand, the execution of these activities will be decentralized. The activities are subdivided in 7 **Work Packages**. For each Work Package one of the partners is made responsible. The responsible partner or **Work Package Leader** coordinates a taskforce designated to achieve the goals set out in the Work Package description. The overall planning of the PREVENTCD project is given in part 7 (Workplan) and in the Gantt charts. For the planning the following procedure will be followed. Every three months, progress will be reported at the Management Team meetings. In case of changes in planning or research, the Work Package Leaders will propose measures needed to realise the Project's goals. The management will decide minor changes in the execution of the project. The Project Consortium will decide on major changes. However, it is expected that there will be no large deviations from the overall work plan submitted in this proposal.
Every three months the Management Team will make an evaluation of the progress of the project based on material that has been submitted to the project database and on short progress reports submitted to the coordinator. These reports should at least contain descriptions of the deliverables and milestones achieved and a self-evaluation of the progress and difficulties encountered. Because of the close coupling of planning and control within a three months cycle, the Management Team will be able to control

the project closely and to adjust the various building blocks where needed, or shift activities between partners.

Role of the Partners

Each partner forms part of a larger network of related institutes and research centres in Europe and has a track record in performing and coordinating large nationally projects, funded on a national or European level. A partner will participate in several other Work Packages as described in the work plan (part 7). Each partner has the right to re-allocate resources in the Work Package, subject to the prior permission of the management, the Project Council and the EC representative. Adjustments in the Work Package resulting in other and/or delayed milestones and deliverables require prior permission of the Management Team.

Communication

The communication in the PREVENTCD project will first of all be based on the Internet. A website will be maintained in which all information is brought together. This information is accessible to the partners and to the research community at large whenever appropriate. An area with restricted access will be used for communication on:

1. Management issued and storage of used documents (i.e. reporting formats);
2. Research data, results and discussions (see WP 7).

Twice a year **progress meetings** will be organized in which all the partners are expected to participate. These progress meetings will also have a formal role in the management of the project, because at these meetings the progress report will be presented and decisions will be made with respect to the plans for the next period. The partners will be obliged to submit their contributions for this report in time. Other means of communication, like e-mail, telephone calls or telephone conferences, will also be used when useful or necessary to fulfil the obligations of the project.

Intellectual Property Rights and Exploitation

The PREVENTCD partnership functions as an European Interest Group in the sense that the partners strive to realise joint objectives in order to establish scientific bases for effective prevention of the most common food sensitivity in Europe (CD). There may be a need to explore the role of intellectual property rights (IPR) protection for

research results derived from the project. The partners are members of larger research organizations and universities which have gained considerable experience in protecting IPR and in the exploitation of knowledge. It should not be difficult to involve experts from within the partners' institutes. The results of PREVENTCD will be scanned (yearly and before publication of results) for IPR potential by the IPR specialists employed by the participants. The involvement of three industrial companies and patient organisations ensures that results will be implemented and commercialized in (baby) food and diagnostics. Through national networks several participants have contacts with other industrial stakeholders. These will be contacted if the use of results is foreseen in other areas than the present industrial participants are working. IPR issues will be arranged in a Consortium Agreement.

6.2 Plan for using and disseminating knowledge

The results of PREVENTCD will be disseminated beyond the consortium as described in WP7. Stakeholders and users are public health professionals, patients (through patients' associations), researchers, regulatory bodies, governments, and the European Commission. These will be reached by a mix of communication measures like the website, newsletters, presentation at conferences and meetings, publications in journals, and new guidelines. The targeted dissemination ensures exploitation/use of the results in the public health sector.

The results of PREVENTCD will, if appropriate, take the form of policy statements on a European Common Strategy to prevent CD. PREVENTCD will offer new, evidence-based, European Guidelines for early feeding practices, including gluten exposure and breast-feeding. Such results will be presented to the scientific community as a position paper and to the coeliac patients and the Europeans in general in the form of flyers and on-line publications. Regulations, rights and obligations with respect to prior knowledge (prior defined as preceding the date of contract signature) will adhere to standard regulations defined in the model contract.

6.3 Raising public participation and awareness

The results of the project will be widely disseminated to other directly interested parts, such as food the industry and patients' associations. Once the strategies for primary prevention of CD will have been identified, the ongoing integrated research

collaboration network will facilitate the dissemination of the new findings, in that it promptly will reach and be of benefit to the European citizens' health and well-being.

7. Workplan– for whole duration of the project

7.1 Introduction - general description and milestones

Our hypothesis is that early dietary history, i.e. the introduction of small quantities of gluten during the period of breast-feeding, may prevent coeliac disease (CD) in genetically predisposed individuals by induction of tolerance for gluten and for other related auto-antigens.

Through integration of novel collaborative research among European leaders from different and crosscutting areas we will examine the complex interactions and molecular mechanisms involved in the development of CD including:

1. Genetic background: stratification by genetic risk in the individual and the family;
2. Early immune response to gluten introduction;
3. Early dietary history including:
    c. Gluten introduction and
    d. Breast-feeding

To identify safe strategies for primary prevention of CD in genetically predisposed children.

Our strategy will include a roadmap for the integration of nutrition intervention studies, both in a cohort of children with a high-risk for developing CD as in the general population, with genetic and immunology response trials where the early dietary history in relation to the development of CD is considered.

The project will be achieved by collaboration of European crucial players in the field of CD, not only in the basic scientific community, but also in clinical practice, food industry, Public Health and patient organizations, determined to pro-actively cooperate on an integrated European research project on effective prevention of CD. Our project represents an example of Transitional Medicine aiming to integrate the knowledge from basic science into clinical applications providing solutions to the problems of the Europeans suffering from CD.

To achieve our objectives we propose the following structure and methodology:

1. A prospective early dietary intervention study in 1000 young children from high-risk families for CD with at least one case of CD among the siblings and

/or the parents, to assess the possibilities to induce immune tolerance for gluten in genetically predisposed children;

2. A prospective dietary intervention population study in 16.000 Swedish children aged 12 years, born during and after the Swedish epidemic of CD, to assess the late effect of dietary history, concerning gluten exposure and breast-feeding, on the development of CD and related autoimmune phenomena.

The work of the proposed STREP will be integrated in the following Work Packages (WP):

- WP 1. Management
- WP 2. Start-up phase
- WP 3. Family study: European early feeding intervention study in infants from high risk families for CD
- WP 4. Population based study exploring birth cohorts with different early dietary history
- WP 5. Genetics
- WP 6. Immunological studies on early response to gluten introduction
- WP 7. Dissemination

7.2 Workplanning and timetable

The work planning of the project, showing the timing of the different Work Packages (WP's) and their components is presented in figure 2 (Gantt chart).

Months project																				
Y	**1st**				**2nd**				**3rd**				**4th**				**5th**			
WP	3	6	9	12	15	18	21	24	27	30	33	36	39	42	45	48	51	54	57	60
1																				
2																				
3																				
4																				
5																				
6																				
7																				

(Y= year of the project; WP = work package)

The proposed STREP requires duration of 5 years because:

1. The 1000 infants from the family study are enrolled during pregnancy, born during the period of the project and CD can be assessed at the age 2-3 years.
2. The time required to carry out the large population study in 2 cohorts of 8.000 children

7.3 Graphical presentation of work packages

Figure 3

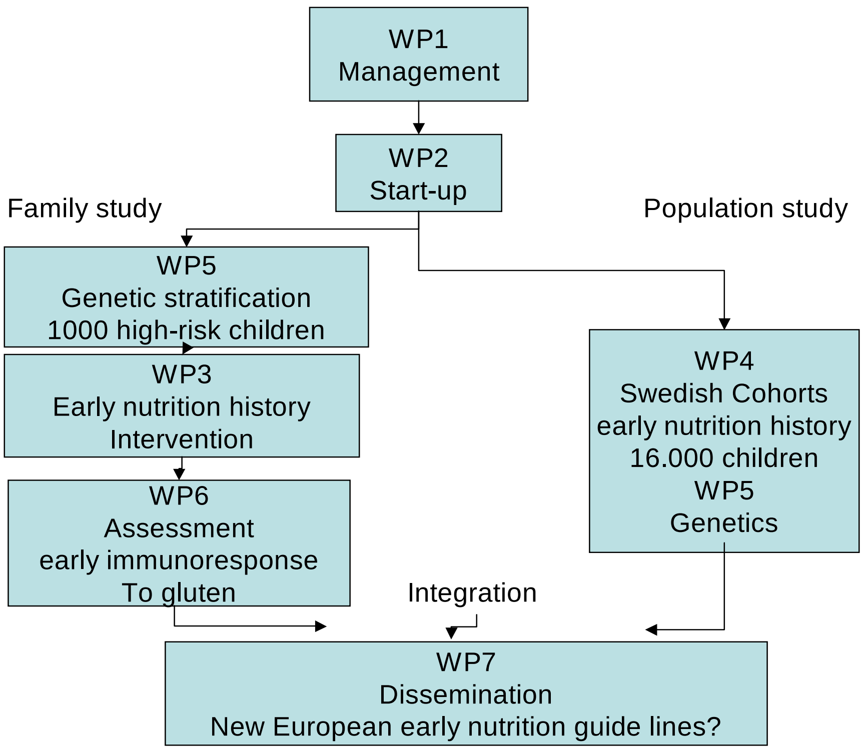

7.4 Work package list /overview

Work package list (full duration of project)

Work-package No[1]	Workpackage title	Lead contractor No[2]	Person-months[3]	Start month[4]	End month[5]	Deliverable No[6]
1	Management	1	63	1	60	D1.1 – D1.7
2	Start-up phase	1	3	1	3	D2.1 – D2.2
3	Intervention family study	2	410	1	60	D3.1 – D3.6
4	Intervention population study	3	142	1	60	D4.1 – D4.3
5	Genetic studies and risk stratification	5	104	1	60	D5.1 – D5.6
6	Immunological studies on early response to gluten introduction	1	88	1	60	D6.1 – D6.6
7	Dissemination	4	15	4	60	D7.1 – D7.8
	TOTAL		825			

[1] Workpackage number: WP 1 – WP n.
[2] Number of the contractor leading the work in this workpackage.
[3] The total number of person-months allocated to each workpackage.
[4] Relative start date for the work in the specific workpackages, month 0 marking the start of the project, and all other start dates being relative to this start date.
[5] Relative end date, month 0 marking the start of the project, and all ends dates being relative to this start date.
[6] Deliverable number: Number for the deliverable(s)/result(s) mentioned in the workpackage: D1 - Dn.

7.5 Deliverables list

Deliverables list

Del. no. [7]	Deliverable name	WP no.	Lead partici-pant	Estimated person-months	Nature [8]	Dissemination level [9]	Delivery date [10] (project month)
D1.1	Project office	1	1	46	O	PP	M1
D1.2	Bi-annual progress reports	1	1	3	R	CO	M6,18,30,42,54
D1.3	Annual progress reports	1	1	2	R	CO	M12, 24,36,48,60
D1.4	Quarterly progress management reports	1	1	3	R	CO	M3,9,15,21,
D1.5	Mid term review	1	1	3	R	PP	M24
D1.6	Final report	1	1	3	R	PP	M 60
D1.7	Mid-term assessment report	1	1	3	R	PP	M25
D2.1	Digital standardized forms	2	1	2	P	CO	M 4
D2.2	Digital databank for data management	2	1	1	P	CO	M 6

[7] Deliverable numbers in order of delivery dates: D1 – Dn
[8] Please indicate the nature of the deliverable using one of the following codes:
R = Report
P = Prototype
D = Demonstrator
O = Other
[9] Please indicate the dissemination level using one of the following codes:
PU = Public
PP = Restricted to other programme participants (including the Commission Services).
RE = Restricted to a group specified by the consortium (including the Commission Services).
CO = Confidential, only for members of the consortium (including the Commission Services).
[10] Month in which the deliverables will be available. Month 1 marking the start of the project, and all delivery dates being relative to this start date.

D3.1	European databank on the interaction between early gluten intake and breast-feeding in children from high-risk families for CD, their early immune response to gluten and their genetic background	3	2	90	P	CO	M 54
D3.2	Report on the possibilities to achieve primary prevention for CD in genetically predisposed children by early dietary history intervention	3	2	5	R	CO	M 56
D3.3	New European guidelines for early nutrition, concerning gluten introduction and breast-feeding	3	2	15	P	CO	M 60
D3.4	350 newborns enrolled	3	2	100	O	CO	M 12
D3.5	700 newborns enrolled	3	2	100	O	CO	M 24
D3.6	1000 newborns enrolled	3	2	100	O	CO	M 36
D4.1	Report on the cohort from the CD epidemic (1993)	4	3	42	R	CO	M 30
D4.2	Report on the cohort from the post-epidemic period (1997)	4	3	50	R	CO	M 54
D4.3	Report combining the results from the 2 cohorts	4	3	50	R	CO	M 56
D5.1	Protocol to capture the genetic variation for the four genetic loci	5	5	20	P	CO	M6
D5.2	Database to store patient and genotype information	5	5	20	P	CO	M6
D5.3	6-months updates on the collection of DNA samples for the biobank	5	5	20	R	CO	M6, 12,18,24,30,36.42 ,48, 54, 60
D5.4	Biobank containing DNA from the population-based cohort study and the family-based cohort study (approx. 4.000 samples)	5	5	20	P	CO	M38

D5.5	Full genotype data for *HLA*, gene *XX, CTLA4,* and the *CELIAC2* locus in CD cases and in the controls	5	5	20	R	CO	M38
D5.6	Report on the genetic risk profiles and distribution of CD across Europe	5	5	4	R	CO	M60
D6.1	Quantitative and qualitative analysis data of the composition of the gluten used in the dietary intervention	6	1	17	R	CO	M36
D6.2	Collection of breast-milk samples	6	1	3	O	PP	M50
D6.3	Quantitative and qualitative analysis data of the composition of gluten present in breast-milk of the mothers participating in the study	6	1	17	R	CO	M54
D6.4	Analysis data of the repertoire of gluten specific T cells in children developing CD during the intervention family study	6	1	17	R	CO	M54
D6.5	Analysis data of the immunological impact of the dietary intervention on the regulatory T cell compartment	6	1	17	R	CO	M56
D6.6	Analysis data of the spectrum of mucosal immune abnormalities in "at-risk" infants with CD in relation to their genetic background	6	1	17	R	CO	M56
D7.1	Publications and information on the project website	7	4	1	R	PU	M4,13,19,25,31,37,43, 48, 54, 60
D7.2	Newsletters	7	4	1	R	PU	M4,13,19,25,31,37,43, 48, 54, 60
D7.3	Presentation at relevant conferences and meetings (twice a year)	7	4	1	D	PU	To be determined
D7.4	Website (EXTRA-net)	7	4	3	O	PU	M6

D7.5	Publications in relevant national and international journals, both in paper and electronic form.	7	4	1	R	PU	M60
D7.6	Scientific based Guidelines on the introduction of gluten into the infants diet to be prepared together with the ESPGHAN Committee on Nutrition	7	4	1	R	PU	M60
D7.7	Notes for European policy makers and international (e.g. Codex Alimentarius) regulatory bodies	7	4	1	R	PU	M60
D7.8	Slide presentation, brochure, and CD/DVD leaflet with educational information on CD and on the project	7	4	6	R	PU	M12
			**TOTAL**	**825**			

7.6 Work package descriptions

Milestones list

Milestone no.	Milestone name	Delivery date[11] (project month)
M1	Opening project office	m1
M2	Kick-off meeting	m3
M3	First year analysis (m 12)	m12
M4	Mid-term analysis	m24
M5	Presentation of the results during the final meeting	M60
M6	Opening web-site of the project (m3)	m3
M7	Submission of 2 papers for publications in scientific journals (m48)	M60
M8	Submission of guidelines on optimal gluten introduction	M60

[11] Month in which the deliverables will be available. Month 1 marking the start of the project, and all delivery dates being relative to this start date.

Only person months in WP1 (management) are changed into 60 months. The work done in the other WP's will not be higher than the original person months.

Work Package (WP) 1. Management

Introduction

The management and governance structure of PREVENTCD ensures effective and optimised management and decision making and enhances networking between the participants and stakeholders (such as patients' organisations, public health authorities and research initiatives on CD) and is described in detail, including the risks and contingency plans under "6.1 Project management". The management and governance structure of PREVENTCD are presented in figure 1. The coordinator and her team will ensure that reports (periodic- mid-term- and final reports, including cost statements and audit certificates) are prepared and submitted to the EC project officer on time and according to instructions.

**Work package number**	1				**Start date or starting event:**							Month1					
**Activity Type**[12]	Management																
**Participant id**	1	2	3	4	5	6	7	8	9	10	11	12	13	14	15	16	17
**Person-months per participant:**	60	1	1	1	1	1	1	1	1	1	1	1	1	1	1	1	1

Objectives

- Coordination by ensuring effective day-to-day work affecting the project
- Management of the project facilitating the roadmap of activities necessary to achieve the objectives, milestones and deliverables.
- Production of reports containing accounting information, descriptions of the achieved deliverables and milestones and a self-evaluation of the progress and difficulties encountered.

Description of work

- A project office, with a project assistant and a data manager, will be open.
- The project assistant and the data manager will support the coordinator in her tasks and will be responsible for maintaining the web-based and networking activities.
- The coordinator, ML Mearin, is responsible for the financial management, facilitating the allocation of the funding to achieve the objectives of the project. She produces accounting information.

The coordinator is responsible for the operational management, chairs the Management Team, is the contact person for the Advisory Board and for the EU Commission and produces reports of the work achieved.

[12] For STREPs/STIPs each WP must relate to one (and only one) of the following three possible Activity Types: RTD/Innovation activities, Demonstration activities, Management activities.

Deliverables

D.1.1 Project office with a project assistant and a data manager (m1)
D.1.2 Bi-annual progress reports (m6, m18, m30, m42, m54)
D.1.3 Annual progress reports (m12, m24, m36, m48)
D.1.4 Quarterly progress management reports (m3, m9, m15, m21, m27, m33, m39, m45, m51, m57)
D.1.5. Mid term review (m 24)
D.1.6 Final report (m60)
D1.7 Mid-term assessment report (m25)

Milestones and expected result

M1. Opening project office: m 1

The final report will contain an overall description of the work, the results achieved and accounting information.

Work Package (WP) 2. Start-up phase

Introduction

This WP is necessary to get the infrastructure ready to carry out the project and it ends with the "Kick-off meeting". During this WP the project-office and website will be open, the personnel involved in the project will be contracted and the digital standardized forms and databanks will be developed. During the start-up phase, the frequency food questionnaires (FFQ's) will be adapted in each participating country to their own eating pattern and their own brands of food products. No contingencies or risks are expected.

**Work package number**	2	**Start date or starting event:**							Month1							
**Activity Type**	RTD/Innovation															
**Participant id**	1	2	3	4	5	6	7	8	9	10	11	12	13	14	15	16	17
**Person-months per participant:**	3	0	0	0	0	0	0	0	0	0	0	0	0	0	0	0	0

Objectives

- To get the project started
- Development of the infrastructure to carry out the project

Description of work

- Development of soft-ware necessary for the project
- Development of the INTRA-net of the project
- Organization Kick-off Meeting

Deliverables

D.2.1 Digital standardized forms to be used for the assessment of the children in the family study (m4)
D.2.2 Digital databanks for data management (m6)

Milestones and expected result

M2. Kick-off meeting (m3)

Work Package (WP) 3: European early feeding intervention study in infants from high risk families for coeliac disease (CD).

Introduction

We will carefully assess the early immune response to gluten in 1.000 infants from high-risk families for CD from different European countries and with different degrees of genetic predisposition for the disease, under different nutritional conditions, including gluten introduction, gluten exposure and breast-feeding practices.

- Design: double blind prospective randomised food intervention study. During the start-up period of the project, due to delay in obtaining the gluten preparation for the intervention on time, after informed consent of their parents-guardians, the recruited children received placebo in a non-randomised, open way.
- Invitation to participate: the families will be invited to participate by the Association of European Coeliac Disease Societies (AOECS) (www.aoecs.org) or by local Coeliac Disease Societies. The CD Societies from the Netherlands (NCV) (www.coeliakievereniging.nl); Campania, Italy (www. rcm.napoli.it/celiachia); Madrid (SCM) (www.celiacosmadrid.org), Valencia (ACECOVA)**,** Catalonia (SMAP) (www.celiacscatalunya.org), Spain; Israel (CAI), Poland (PCS), Croatia, Hungary **(**www.coeliac.hu**)** and Germany **(**www.dzg-online.de**)** will invite their members with CD expecting a newborn (child or sibling) during the next 18 months to participate. Partner 9, the AOECS, is able to easily link the project to each country in Europe, in particular those member states with large populations, in order to ensure the necessary number of infants. (table 2).
- Informed consent: during pregnancy, the families will be asked informed consent for participation by the local responsible physician.
- Enrolment: after informed consent is given, the families will be enrolled in the study. Breast-feeding during at least six months will be STRONGLY encouraged to all the families.
- Genetic risk stratification: the genetic risk for CD will be assessed and stratified as described in WP 5 “Genetics”.
- Randomisation to "tolerance induction for gluten" or to the "control" group. Shortly before the dietary intervention, the children bearing HLA-DQ2 and/or

DQ8, and who consequently may at some time develop CD, will be randomised to the group for "tolerance induction for gluten" or to the "control" group (table 1). The randomisation is performed by partner 1 (LUMC, dept. of Medical Statistics). The randomistion codes will be kept by the department of Medical Statistics LUMC.

- Intervention

  At the age of 4 months the blind intervention will take place. This age is chosen because it is known to represent a “window of opportunity” to introduce grains into the diet, with respect to development of autoimmune phenomena. The present evidence over this “window of opportunity” is based on recent publications covering, among others, two studies in Germany and USA. The American study concerns the first prospective observational study that attempts to outline a link between early infant diet and development of CD (19). The study was performed on a cohort of 1560 children derived from the DAISY project (Diabetes Autoimmunity Study in the Young), who had an increased risk of developing CD or type1 diabetes, as defined by possessing either HLA-DR3 or DR4 alleles, or having a first-degree relative with type 1 diabetes. At a mean follow-up of 4.8 years, the authors concluded that: 1) there is a “window of opportunity” of introducing gluten into the diet when the child is aged between 4 and 6 months with regard to the risk of developing CD, and 2) that the contribution of breast-feeding was to be disregarded in this respect. However, the authors did not make specific attempts to calculate the gluten amount ingested by the children or to correlate this important early nutrition event with the presence or absence of breast-feeding. The German BABYDIAB study followed 1610 newborn children of parents with type 1 DM and found that food supplementation with gluten-containing foods before age 3 months was associated with significantly increased islet autoantibody risk (18). A systematic review and meta-analysis of observational studies published between 1966 and June 2004 that examined the association between breast feeding and the development of CD has been published (20). This study gives also support for the concept that breast feeding during the introduction of dietary gluten and increasing duration of breast feeding are associated with reduced risk of developing CD. Prospective cohort studies, like the one proposed here by us, may shed light on the importance of the quantity of exposure to gluten in early life in the development of CD (21).

- o <u>Tolerance induction</u> will attempted in the randomised group of children by blind daily intake of 1g wheat flour (100 mg gliadin, approximately 7-10 % of the gluten intake at age 1 year) during 8 weeks. Gluten is a common name used for some proteins (prolamins and glutenins) of wheat, barley, rye, and oats. Being gluten a naturally occurring component of food, it should be considered a food that is supplemented. Compliance will be assessed by 2 weekly visits or interviews.

  <u>The control intervention</u> will take place by blind daily intake of 1g. lactose during 8 weeks.

Danone will provide the placebo (lactose, Pharmatose) and the intervention product (Glutival obtained from Cargill). Danone has outsourced the production of the identical sachets with the gluten intervention / placebo products to NIZO Food Research, Ede, the Netherlands.

NIZO will prepare and package the gluten intervention product as well as the placebo according to quality criteria adequate for infants aged 4-6 months..

Every intervention sachet will be filled 1.8 gr Pharmatose and 0.2 gr Glutival (gluten intervention product); the placebo sachets will be filled with 2 gr of Pharmatose.

NIZO will pay special attention to the microbiological assessment of the products and will make sure that the results will be in accordance with the standards of Danone. Also it will be analytically verified whether the blending of the materials has been adequate and resulted into a homogeneous mixture.

- <u>Monitoring of early immune response to gluten in all the children</u>: see Description of WP 6 “Immunological studies on early response to gluten introduction” and table 1.
- <u>Nutritional assessment:</u>

  After the intervention, at the age of 6 months, all the families will be advised to gradually introduce gluten into the diet of their children. The parents will be advised to give the children 500 mg of gluten at the age of 7 months (10 g of ordinary biscuit), 1000 mg at 8 months (10 g semolina and 10 g biscuits), 1500 mg at 9 months (20 g semolina, or pasta, and 10 g biscuits). After that, the children will consume "ad libitum" quantities of gluten-containing products. The use of breast-feeding and the quantities of gluten ingested will be assessed using

food frequency questionnaires (FFQ's) recently developed and validated by the Dutch partner.

Table 1. Time scheme of the assessments in the enrolled children

Months	Pregn ancy	Birth	1	2	3	4	5	6	7	8	9	10	11	12	14	16	18	20	22	24	28	30	34	36	48	
Enrolment	+																									
Randomisation					+																					
Intervention						+																				
Anthropometrics		+	+	+	+	+	+	+			+			+			+			+				+	+	
Clinical History		+	+	+	+	+	+	+	+	+	+	+	+	+	+	+	+	+	+	+	+	+	+	+	+	
Food Questionnaire		+	+	+	+	+	+	+	+	+	+	+	+	+	+	+	+	+	+	+	+	+	+	+		
Cord blood Sample Genetic study		+																								
Breast milk Sample			+	+	+	+	+	+			+															
Blood sample Immunological Studies						+		+			+			+			+			+				+	+	

- Follow-up 0-4 years: All the children will be frequently controlled for clinical and nutritional assessment and for the presence of immunological markers of gluten (in)tolerance, including determination of serum IgA antibodies against gliadin and tissue trans- glutaminase (tTGA) (table 1). In case of health complaints extra controls will take place and, if indicated, the intervention will be stopped.
- Diagnosis of CD: The parents of the children with tTGA or symptoms of CD will be offered a small bowel biopsy for the diagnosis of CD. Biopsie will only be performed when medically indicated, that is: only in these children with CD antibodies in their serum that indicate gluten sensitivity and are highly suspect for active CD, and NOT just for purpose of the study. Such children would undergo a biopsy also in non-study circumstances.

  A reduction of 50% of CD among the intervention group at the age of 3 will be considered as an effective prevention.
- Power Analysis:(80%, significance level 5%, 2-sided test): Assuming a frequency of 10% CD among the enrolled infants we need to study 474 children randomized to tolerance induction and 474 controls. The number of members of the participating Coeliac Disease Societies (table 3) makes the study feasible.

Table 2. Members of the coeliac disease patient societies collaborating in the project

CD Societies	Members (no.)	0-10 years (siblings) (no.)	Female Fertile age (mothers) (no)	Male Fertile age (fathers) (no.)	Expected newborns to be recruited (no.)
Netherlands	7902	1494	670	390	150
Campania	3000	800	300	100	150
Madrid	5133	2413	387	162	100
Valencia	2050	475	152	67	75
Israel	4800	800	470	830	100
Poland	7500	1200	n.k.***	n.k.***	100
Croatia	470**	400	n.k.***	n.k.***	50
Catalonia	6000	2300	1319	1680	75
Hungary	2800	660	850	410	100
Germany	33.335	2767	5400	2040	100
Total					1000

(*=invited to participate; the local societies are members of the AOECS; ** officially registered, non-officially more than 1800; ***not know).

**Work package no.**	3				**Start date or starting event:**						Month1						
**Activity Type**	RTD/Innovation																
**Participant id**	1	2	3	4	5	6	7	8	9	10	11	12	13	14	15	16	17
**Person-months per participant:**	54	54	0	38	0	38	30	6	0	13	12	42	0	27	38	20	38

Objectives

Assessment of the possibilities to achieve primary prevention for CD in genetically predisposed children by early dietary intervention consisting of introduction of small quantities of gluten during the period of breast-feeding

Description of work

- Assessment of the early immune response to gluten in approximately 1000 infants from high-risk families for CD from different European countries. The families will be invited to participate, among others, by the local Coeliac Disease Societies in collaboration with the Association of European Coeliac Disease Societies (AOECS). The CD Societies from the Netherlands (NCV)

(www.coeliakievereniging.nl); Campania, Italy (www. rcm.napoli.it/celiachia); Madrid (SCM) (www.celiacosmadrid.org), Valencia (ACECOVA), Catalonia (SMAP) (www.celiacscatalunya.org), Spain; Israel (CAI), Poland (PCS), Croatia, Hungary (www.coeliac.hu) and Germany (www.dzg-online.de) will collaborate in the study.

- The Societies will invite to participate their members with CD expecting a newborn (child or sibling) during the next 18 months. Informed consent to participate will be asked from the families by the local responsible physician.
- The children bearing HLA-DQ2 and/or DQ8 will be blindly randomised to either a group for "tolerance induction for gluten" or to a "control" group. At least 6 months of breast-feeding will be STRONGLY encouraged for all the children. At the age of 4 months tolerance induction will be attempted by the daily intake of 1g wheat flour (100 mg gliadin) during 8 weeks while continuing breast-feeding. No gluten will be given in these 8 weeks to control infants, but 1g. lactose as a placebo intervention. Danone will provide the placebo (lactose, Pharmatose) and the intervention product (Glutival obtained from Cargill). Danone has outsourced the production of the sachets with the gluten intervention / placebo products to NIZO Food Research, Ede, the Netherlands. NIZO will prepare and package the gluten intervention product as well as the placebo according to quality criteria adequate for infants aged 4-6 months. Every intervention sachet will be filled 1.8 gr Pharmatose and 0.2 gr Glutival (gluten intervention product); the placebo sachets will be filled with 2 gr of Pharmatose.
  NIZO will pay special attention to the microbiological assessment of the products and will make sure that the results will be in accordance with the standards of Danone. Also it will be analytically verified whether the blending of the materials has been adequate and resulted into a homogeneous mixture. Compliance will be assessed by visit or interview.
- After the 8 weeks intervention period, all the families will be advised to gradually introduce gluten into the diet of their children, and to increase it gradually till the age of 12 months, for which simple recommendations will be provided to the parents.
- The infants will be strictly followed up (Table 1). Blood (5 ml) is sufficient to allow for the screening of CD-specific antibodies, phenotypic characterization of 20 markers indicating lymphocyte activation and regulatory T cell induction, and monitoring for the occurrence of gluten-specific T cell responses typical for CD. At age 3 years 10 ml blood will be obtained to test for additional (auto)immune phenomena related to the development of CD.
- Children with positive antibodies strongly indicating CD or with clinical suspicion of CD will be offered a small bowel biopsy for the definitive diagnosis a reduction of 50% of CD among the intervention group at the age of 3 will be considered as an effective prevention.

Deliverables

D.3.1 European databank on the complex interaction between early gluten intake and breast-feeding in children from high-risk families for CD, their early immune response to gluten and to other related auto-antigens and their genetic background (m 44)
D.3.2 Assessment of the possibilities to achieve primary prevention for CD in genetically predisposed children by early dietary history intervention (m 46)
D.3.3 New European guidelines for early nutrition, concerning gluten introduction and breast-feeding (m 48)
D.3.4 350 newborns enrolled (m12)
D.3.5 700 newborns enrolled (m 24)
D.3.6 1000 newborns enrolled (m 36)

Milestones and expected result

M3. First year analysis (m 12)
M4. Mid-term analysis (m 24)
M5. Presentation of the results during the final meeting (m 60)

Development of a common European databank on the complex interaction between early gluten intake by European young children with a high-risk for CD, their early immune response to this food antigen and their genetic background

Work Package (WP) 4: Population based study exploring birth cohorts with different early dietary history

Introduction

We will take advantage of the “experiment” in the Swedish population with extensive changes in infant feeding practices within a few years, which resulted in a unique epidemic of symptomatic CD in children below 2 years of age. The incidence rate of symptomatic CD increased four-fold to levels higher than ever previously reported, and after a ten-year period on this high level, suddenly returned back to the previous level. A representative sample of 16.000 Swedish children aged 12 years, 8.000 born during the peak of the epidemic (1993) and 8.000 born after the epidemic (1997), will participate in the study. This study is based on a long-standing collaboration with paediatric units in different parts of Sweden, and also a well-developed collaboration with the school health services.

- Design: prospective population based intervention study with a long-term follow-up
- Study base: the study on the 8.000 children from the epidemic will be done before the project, but the results will be used to be compared with the data from the cohort from the post-epidemic period.
- Invitation to participate: requirement for inclusion is an informed consent from the child and its parents.
- Symptomatic CD cases diagnosed before the age of 12-years will be identified through the National Register of CD in Swedish children.
- Screening for CD: all children will be invited for a blood sample during the school-year 2009-2010 for determination of tissue transglutaminase antibodies type IgA (AtTG), and those with s-IgA-deficiency also for AtTG type IgG. When CD is suspected the child is referred for a diagnostic small bowel biopsy. Biopsies will only be performed when medically indicated, that is: only in these children with CD antibodies in their serum that indicate gluten sensitivity and are highly suspect for active CD, and NOT just for purpose of the study. Such children would undergo a biopsy also in non-study circumstances.

- Double the numbers of screening-detected cases in the epidemic cohort are expected compared to the cases in the post-epidemic cohort.
- HLA and non-HLA genetic characteristics and autoimmune serological markers will be assessed in all the children with CD (symptomatic and screening-detected) and in 5 population based controls per case (n=1800).
- Early dietary history: The parents will be asked to fill in a questionnaire about the child's history with respect to breast-feeding and early gluten exposure.
- Statistical power analysis: A sample size of 5.000 participants from each cohort is necessary to determine an expected difference in prevalence of CD between the cohorts of the epidemic (1993) and post-epidemic (1997) periods. However, to get enough CD cases also for sub-studies the number of participant invited children will be 8.000 in each cohort.

**Work package no.**	4						**Start date or starting event:**				Month 1						
**Activity Type**	RTD/Innovation																
**Participant id**	1	2	3	4	5	6	7	8	9	10	11	12	13	14	15	16	17
**Person-months per participant:**	0	0	116	0	0	0	0	0	0	7	19	0	0	0	0	0	0

Objectives

- Determination of the possible achievement of primary prevention of Coeliac Disease (CD) and related auto-immune phenomena by favourable early dietary practices in the general population
- Evaluation of the interaction between early dietary history and CD related genetic factors known to us on the prevalence and expression of CD in the general population.

Description of work

- The study base is defined by a representative, population based sample of children born in 1993 and 1997, 8.000 from each cohort, using the Swedish population register. The study on the first cohort (1993) will be finalized before this project starts. However, the results will be brought into the project. The study on the second cohort (1997) falls within the project.
- All cases of symptomatic CD diagnosed before the age of 12 will be identified through the National Register of CD in Swedish children.
- All children will be invited to a screening for CD at the age of 12 including blood sampling and parental questionnaires (on e.g. early dietary history and autoimmune diseases).
- Blood samples of all children will be analysed for tissue transglutaminase antibodies (TTGA), and when CD is suspected a diagnostic small intestinal mucosa will be evaluated we expect to find double

the number of screening-detected cases in the cohort of 1993 compared to the one of 1997.

- Blood samples from all children with CD (symptomatic and screening-detected), from those with an isolated aberrant TTGA response, and from five population based controls per case (n=1800) will be used for HLA and non-HLA genetic analyses (WP5), and for determination of auto-antibodies.
- Questionnaire data on early dietary history and auto-immune diseases will be compared between the CD cases (symptomatic and screening-detected), and population based controls, as differences between these groups and also between the cohorts of 1993 and 1997 are expected.
- The prevalence of CD, both symptomatic and screening-detected, will be compared between the two birth cohorts, and it will be related to differences in early dietary history and genetics (WP5).

Deliverables

D.4.1.Report on the cohort from the CD epidemic (1993) with respect to prevalence of CD and related autoimmune phenomena, n relation to early dietary history and HLA profile (m 30).

D.4.2.Report on the cohort from the post-epidemic period (1997) with respect to prevalence of CD and related autoimmune phenomena, n relation to early dietary history and HLA profile (month 42).

D.4.3 Report combining the results from the 2 with respect to prevalence of CD and related autoimmune phenomena in relation to early dietary history and HLA and non-HLA genetic profile (month 44).

Milestones and expected result

M3. First year analysis (m 12)
M4. Mid-term analysis (m 24)
M5. Final meeting: presentation of the results (m 60)

This population based study exploring birth cohorts with different early dietary history, and with a follow-up of twelve years, gives an opportunity to determine the late effects of breast-feeding and early gluten exposure involved in the primary prevention of CD.

Work Package (WP) 5: Genetic studies and risk stratification

Introduction

There is a strong inherited predisposition to CD susceptibility. Around 90% of CD patients possess the HLA-DQ2 and/or HLA-DQ8 molecules. However, the HLA-DQ2 allele is common in the healthy population, carried by approximately 30% of Caucasians. Therefore, genetic factors outside the HLA region contribute to disease susceptibility or resistance. To date, at least three non-HLA loci have been identified that are likely to contain disease predisposing variants, on chromosome 2q (*CELIAC3* locus), on chromosome 5q (*CELIAC2* locus), and on chromosome 19p (*CELIAC4* locus). For two of these regions the disease-causing genes have been identified: the *CTLA4* gene on 2q and the *XX* gene on 19p. *CTLA4* confers an approximately a 1.5-fold increased risk to CD and gene *XX* a 2.3 fold risk. Eleven genome-wide searches for linkage regions have been performed in CD, as well as numerous region specific linkage studies. Participant 5 is involved in fine-mapping of two additional CD loci on chromosomes 6q21-22 and 9p13, large-scale genetic testing of some 100 functional candidate genes, and a genome-wide genetic association studies. The data generated through these ongoing efforts will benefit this proposal. Therefore, it is expected that in the next few years additional loci/genes will be added to the current list of CD susceptibility genes and these will then also be included without jeopardizing the proposed plan of work and the deliverables.

In this proposal we will study the effect of the currently known genetic risk factors on CD and the early dietary intervention, in two cohorts: a Swedish population-based cohort (a representative sample of approximately 1.800 individuals from a total cohort of 16.000 individuals) and a European-based family cohort (approx. 3.500 individuals). Each individual will be genotyped for the four genes listed above: HLA-DQ, CTLA4, CELIAC2, *MYO9B*. DNA will be isolated from peripheral blood from all participants according to standard protocols. Genotyping will be performed using 384 single nucleotide polymorphisms (SNP) covering these four regions and analyzed on an Illumina Bead Array system. All samples will be blindly coded to prevent any biases in data-analysis. Appropriate controls will be included to control for genotyping and sample

errors. Statistical analysis will be performed separately for the population-based cohort and the family-based cohort.

Evaluating HLA and non-HLA susceptibility genes in the family-based cohort

Since the families will be collected from different geographic areas we can also study if this affects their genetic risk profile. Upon the early dietary intervention study the gluten-treated CD cases (randomized to tolerance induction) will be compared to the placebo-treated CD cases for their genetic profile to identify differences in distribution of the four genetic risk factors in high risk families.

Evaluating HLA and non-HLA susceptibility genes in the population-based cohort

The population study encompasses in total 16 000 children divided among the birth cohorts of 1993 and 1997. DNA will be available for all CD cases diagnosed before the age of 12, both symptomatic (expected n= 93) and screening-detected (expected n=69), and a representative sample of five controls per identified CD case. Therefore, this cohort is excellently suitable to properly assess the effect of HLA and non-HLA genes at both the individual and the population level.

Genetic testing for the HLA and non-HLA loci will allow evaluation of these genetic factors in relation to the prevalence and the phenotype (symptomatic/screening-detected) of CD. This will allow us to determine the role of both HLA and non-HLA genes on disease-risk in patients and their possible predictive value in the population. In addition, we can determine if these genes are differentially involved in symptomatic *versus* screening-detected CD subjects. Interestingly, since the 1993 and 1997 birth cohorts experienced different exposure with respect to early feeding we will also be able to assess the effect of genetic risk factors on the effect of different early feeding patterns.

**Work package number**	5				**Start date or starting event:**					Month 1							
**Activity Type**	RTD/Innovation																
**Participant id**	1	2	3	4	5	6	7	8	9	10	11	12	13	14	15	16	17
**Person-months per participant:**	0	48	8	0	48	0	0	0	0	0	0	0	0	0	0	0	0

Objectives

- Evaluation of the genetic factors known to us on the prevalence and phenotype of coeliac disease (CD) by exploring the genetic loci/regions on chromosomes 6p21 (*HLA*), 5q31-q33 (*CELIAC2*), 2q33 (*CTLA4*), 19p13 (*MYO9B*) in both a population-based and a high risk family-based cohort.
- Analysis of the effect of the currently known genetic factors on the response to early dietary intervention in a population-based CD cohort and in a high risk family-based CD cohort

Description of work

- Setting up a Biobank with DNA from 1.000 high-risk CD cases and their family members, and in 1800 children from the population cohort (expected 160 CD cases and age and sex matched controls).
- Evaluating the currently known genetic risk factors using high throughput SNP genotyping: the HLA predisposing genes (*DR* and *DQ*), gene *MYO9B* (19p13.1), the *CTLA4* (2q33) gene, and the *CELIAC2* (5q31-q33) locus
- Stratification of subjects in different genetic profile classes and comparing the response to dietary intervention among the classes using logistic regression
- Assess whether if the genetic profiles differ across European CD populations
- Assess whether if the genetic profiles differ between high-risk CD families and population-based CD cases

Deliverables

D.5.1.Single nucleotide polymorphisms (SNP) to capture the genetic variation for the four genetic loci (m6)

D.5.2.Database to store patient and genotype information (m6)

D.5.3 6-months updates on the collection of DNA samples for the biobank

D.5.4.Biobank containing DNA from the population-based cohort study and the family-based cohort study (approx. 4.000 samples) (m38)

D.5.5 Full genotype data for *HLA*, gene *MYO9B* , *CTLA4,* and the *CELIAC2* locus in CD cases and in the controls (m38)

D.5.6 Genetic risk profiles and distribution of CD across Europe (m48)

Milestones and expected result

M3. First year analysis (m 12)
M4. Mid-term analysis (m 24)
M5. Final meeting: presentation of the results (m 60)

<u>Work Package (WP) 6</u>: Immunological studies on early response to gluten

<u>Introduction</u>

The Objectives of this WP are: 1) to determine the impact of early dietary intervention, in terms of gluten exposure and breast-feeding, on the development of a gluten specific T cell repertoire and on the regulatory T cell compartment; and 2) to describe the spectrum of mucosal immune abnormalities in relation to the genetic background.

Gluten analysis

For the early dietary intervention in the family study (WP3), a standardized gluten preparation will be used. There is strong evidence that particular gluten peptides are more frequently recognized by patient derived T cells than others. It is therefore imperative that the composition of the gluten used for the intervention is determined. Recently, we have developed novel methods to carry out such a quantitative and qualitative analysis of the presence of harmful gluten fragments in gluten and we will use these for this purpose.
At random, monsters of the gluten provided for the dietary intervention will be solubilized, treated with pepsin at acid pH, followed by trypsin cleavage at neutral pH. The resulting preparation will be divided in two portions. One for determination of T cell stimulatory gluten fragments from gliadin and glutenin and the other for tests with a panel of patient derived T cell clones specific for gliadin and glutenin, as described previously.

Breast-milk analysis

Breast-milk contains low but measurable amounts of gluten that might have an impact on the early dietary intervention in children from high risk families for CD (WP3). Therefore, we will use the above described monoclonal antibody-based assays to determine the level of gluten exposure of the children enrolled into the dietary intervention due to the presence of gluten in breast-milk. For this purpose breast-milk samples will be collected monthly after birth for quantitative and qualitative analysis of gluten content using an established protocol.

Detection of alterations in gene expression profiles that correlate with gluten introduction and/or disease development.

As discussed extensively during the midterm review the limited amount of blood available for analysis precludes the use of HLA tetramers to analyze the presence of gluten reactive T cells in peripheral blood. Moreover, alternative protocols using IFNgamma and IL-10 ELISPOT assays that have been developed by partners 2 and 13 (UNINA and UO) are also likely to be inadequate for the reliable detection of gluten-specific T cells in the samples available. In agreement with the discussion with the reviewers of the midterm review, we have therefore decided to use the samples for gene expression analysis. As until now the blood samples have been used to isolate serum and lymphocytes we will continue to collect samples in an identical fashion to allow comparison of samples in the future. For the gene expression analysis a series of samples from children that did develop CD and control samples from children that did not develop CD will be thawed and stimulated with PHA in a standard protocol in order to obtain sufficient amounts of cells for isolation of good quality RNA. Subsequently gene expression analysis will be performed with the use of the latest available technology. While gene expression microarrays have been the default technology for transcriptome analysis, the introduction of deep sequencing technology now enables the simultaneous sequencing of up to millions of different DNA molecules. This has been proven to be a powerful technique for the identification of differentially expressed transcripts and allows the detection of differential expressed low-abundant transcripts that are well beyond the reach of classical micro-array analysis. For this approach we will collaborate with partner 5 who has well established expertise in this field.

Repertoire analysis

During the course of the family study, between 50 and 100 children are expected to develop CD. This is by far the largest group of paediatric patients to have been included in a study to determine the repertoire of gluten-specific T cells. Moreover, due to the set-up of the study, it can be expected that the development of symptoms associated with CD

will be noted very early in the disease development. The repertoire analysis will thus give a clue to which T cell responses are the earliest to develop. Finally, the repertoire analysis will reveal whether the early dietary intervention will have an impact on the repertoire development. For this purpose we will make use of established protocols to generate gluten-specific T cells from biopsies of children who developed CD. In short, polyclonal gluten specific T cell lines will be generated from these biopsies by a three day specific stimulation with pepsin/trypsin (+/- tissue transglutaminase) treated gluten followed by one round of a-specific expansion with IL-2. These polyclonal T cell lines will subsequently be tested against synthetic peptides representing previously characterized gluten derived T cell epitopes. T cell proliferation and IFNgamma production will be measured. In particular cases we will generate gluten specific T cell clones to analyse further the fine specificity of the gluten response. Furthermore, we will use ELISPOT to determine the (relative) frequency and specificity of the gluten reactive T cells in these polyclonal T cell lines.

Regulatory T cell compartment

Given the immunogenic nature of gluten, the maintenance of tolerance towards gluten is similar to the results of active regulation of gluten specific T cell responses in healthy individuals, a mechanism that fails in CD patients. In murine models regulatory cells have been identified in the mucosa and were found to differentiate from naive T-cells in the periphery, to be antigen-specific and to suppress irrespective of ongoing Th1 or Th2 cytokine polarization. The mechanism underlying suppression by these mucosal regulatory T cells may include secretion of TGF-β or IL-10, but remains debated. Based on these observations we will test whether the early dietary intervention in children from high risk families induces regulatory T cells that suppress the development of an inflammatory T cell response to gluten. In particular, we will determine whether gluten-specific, IL-10 and/or TGF-β producing cells are present in peripheral blood and biopsy material from individuals included in the early dietary intervention. In these studies we will pay particular attention to the possibility that these cells will be present within the CD4CD25 regulatory T cell compartment. For this purpose, peripheral blood and biopsy

samples from the children who develop CD will be collected and tested against gluten. Subsequently, cytokine secretion by T cell subsets will be measured using established protocols.

Genetic background and mucosal immune response to gliadin

In relation to the early dietary intervention towards "tolerization for gluten" planned in the cohort of newborns from coeliac families, the main outcome will be the development of CD. Its recognition will be based on standard diagnostic criteria (i.e. enteropathy in presence of serum anti-tissue transglutaminase antibodies, possibly in a symptomatic child). Nonetheless, the alterations elicited by the ingestion of gluten are now considered to be a spectrum, ranging from the sole presence of immune abnormalities to minor enteropathy, to the full blown picture of flat jejunal mucosa. We will take advantage of this large cohort of subjects at risk. By means of this cohort we wish to evaluate 1) the relationships between their genetic backgrounds and the extent of the mucosal immune response to gliadin; 2) the natural immunopathological history of gluten sensitivity. In the laboratory of the Department of Paediatrics of the University of Naples a number of tests have been developed in the last decade to measure the degree of mucosal inflammation in relation to the exposition to gluten. They include immunohistochemical analysis of the jejunal mucosa, the mucosal cytokine pattern and the detection of intestinal auto-antibodies. The data will allow us a precise description of mucosal abnormalities in subjects who are at risk.

The amount of blood to be obtained (5 ml) is sufficient to allow for the screening of CD-specific antibodies, phenotypic characterization of 20 markers indicating lymphocyte activation and regulatory T cell induction, and monitoring for the occurrence of gluten-specific T cell responses typical for CD. At the age of 3 years 10 ml blood will be obtained from the children to allow for determination of additional (auto) immune phenomen related to the development of CD.

Work package number	6				Start date or starting event:						Month 1						
Activity Type	RTD/Innovation																
Participant id	1	2	3	4	5	6	7	8	9	10	11	12	13	14	15	16	17
Person-months per participant:	32	24	0	0	0	0	0	0	0	0	0	0	32	0	0	0	0

Objectives

- Determination of the immunological impact of the early dietary intervention on the development of a gluten specific T cell repertoire in children from high risk families for CD.
- Determination of the immunological impact of the intervention on the regulatory T cell compartment.

Analysis of the spectrum of mucosal immune abnormalities in relation to the genetic background.

Description of work

- *Gluten analysis*

The composition of the gluten used for the family intervention study will be determined by using our recent developed method to carry out quantitative and qualitative analyses of harmful gluten fragments.

- *Breast-milk analysis*

We will use monoclonal antibody-based assays to determine the level of gluten exposure in the children enrolled into the dietary intervention due to the presence of gluten in breast-milk. Breast-milk samples will be collected monthly after birth.

- *Detection of alterations in gene expression profiles that correlate gluten introduction/or disease development*

Gene expression profiles visualize gluten specific T cells. Using this technological breakthrough, we will be determinedanalyse the presence of gluten reactive T cells in the peripheral blood samples collected from the children that did develop CDrandomised to tolerance induction for gluten and compared with similar expression profiles of from the children that did not develop CD. randomised to the group of controls.

- *Repertoire analysis*

We will make use of established protocols to generate polyclonal specific T cells from biopsies of children who develop CD. These polyclonal T cell lines will subsequently be tested against synthetic peptides representing previously characterized gluten derived T cell epitopes. T cell proliferation and IFNgamma production will be measured. We will use ELISPOT to determine the (relative) frequency and specificity of the gluten reactive T cells in these polyclonal T cell lines.

- *Regulatory T cell compartment*

We will test whether the early dietary intervention in children from high risk families induces regulatory T cells that suppress the development of an inflammatory T cell response to gluten. We will determine whether gluten-specific, IL-10 and/or TGF-β producing cells are present in peripheral blood and biopsy material. We will pay particular attention to the possibility that these cells will be present within the CD4CD25 regulatory T cell compartment. Peripheral blood and biopsy samples from the children who develop CD will be collected and tested against gluten. Subsequently, cytokine secretion by T cell subsets will be measured using established protocols.

- *Genetic background and mucosal immune response to gliadin*

We will evaluate 1) the relationships between their genetic background and extent of the mucosal immune response to gluten; 2) the natural immunopathological history of gluten sensitivity. Tests to measure the degree of mucosal inflammation in relation to the exposition to gluten include

immunohistochemical analysis of the jejunal mucosa, the mucosal cytokine pattern and the detection of intestinal auto-antibodies.

Deliverables

D.6.1.Quantitative and qualitative analyses of the composition of the gluten used in the dietary intervention (m36);
D.6.2 Collection of breast-milk samples (m 36)
D.6.3 Quantitative and qualitative analyses of the composition of gluten present in breast-milk of the mothers participating in the study (m44)
D.6.4 Analysis of the repertoire of gluten specific T cells in children developing CD during the intervention family study (m 45)
D.6.5 Analysis of the immunological impact of the dietary intervention on the regulatory T cell compartment (m45).
D.6.6 Analysis of the spectrum of mucosal immune abnormalities in infants "at-risk" for CD in relation to their genetic background (m45).

Milestones and expected result

M3. First year analysis (m 12)
M4. Mid-term analysis (m 24)
M5. Final meeting: presentation of the results (m 60)

Work Package (WP) 7. Dissemination

This package is needed to disseminate the activities and results of the project at different levels: namely the scientific community, industry, regulation bodies and patients' associations. The dissemination in the PREVENTCD project will be first of all based on the internet. A website will be maintained in which all information is brought together. This information is accessible to the research community and at large whenever appropriate. Stakeholders and users are public heath professionals, patients and patient's organisations, researchers, regulatory bodies, governments, and the European Commission. The results of PREVENTCD will take the form of scientifically based European early nutrition guidelines concerning gluten introduction and exposure and breast-feeding, as well as the prevention of CD. The dissemination will be reached by a mix of communication measures like the website, newsletters, presentation at conferences and meetings, publications in journals, and new guidelines. The targeted dissemination ensures exploitation/use of the results in the public heath sector. Such results will be presented to the scientific community as a position paper and to the coeliac patients and the general European population in the form of flyers and on-line publications.

The project has also an educational dimension, among others, through the role of partner 9 (AOECS) in WP7. The AOECS is very experienced and generally interested in all matters related to education on CD and its task in this WP is specifically devoted to the preparation of teaching aids on CD which can be used for education and training purposes at all levels. Education shall be targeted not only to the typical CD stakeholders as the families and patients suffering from CD and/or the relevant health professional groups (such as nurses, midwifes, dieticians, paediatricians, etc.), but also to the general public (teachers, pupils, journalists, etc.) The teaching aids and educational material will be prepared in the language of the target population.

Work package number	7				Start date or starting event:					3							
Activity Type	RTD/Innovation																
Participant id	1	2	3	4	5	6	7	8	9	10	11	12	13	14	15	16	17
Person-months per participant:	0	0	0	9	0	0	0	0	6	0	0	0	0	0	0	0	0

Objectives

The objective of this WP is to ensure that the results of PREVENTCD will be widely known to the scientific and general community and that potential users such as industry, regulation bodies and patients' associations will have access to the results.

Description of work

- The project's newsletter will be published quarterly to give information about actual project proceedings and the results, as well as general information about the project
- Website (EXTRA-net) of the project
- Publications in journals
- Presentations at relevant national and international conferences and meetings
- Organization meetings with regulatory bodies, governments, and the European Commission
- Preparation of a multimedia presentation of the project, which will include a slide presentation, a brochure, and a CD/DVD leaflet.
- Preparation of educational information on CD for health professionals (nurses, midwifes, dieticians, paediatricians, etc.) and for the general public (teachers, pupils, journalists, etc.) which will include a slide presentation, a brochure, and a CD/DVD leaflet.

Deliverables

D.7.1 Publications and information on the project website
D.7.2 Newsletters
D.7.3 Presentation at relevant conferences and meetings (twice a year)
D.7.4 Website (EXTRA-net)
D.7.5 Publications in relevant national and international journals, both in paper and in electronic form.
D.7.6 Scientifically based Guidelines on the introduction of gluten into the infants diet to be prepared together with the ESPGHAN Committee on Nutrition
D.7.7 Notes for European policy makers and international (e.g. Codex Alimentarius) regulatory bodies
D.7.8 Slide presentation, brochure, and CD/DVD leaflet with educational information on CD and on the project.

.

Milestones and expected result

M6. Opening web-site of the project (m3)
M7. Submission of 2 papers for publications in scientific journals (m60)
M8. Submission of guidelines on optimal gluten introduction (m 60)

8. Project resources and budget overview
8.1 Efforts for the project (STREP/STIP Efforts Form)

PROJECT EFFORT FORM person months																		
Participant	1LUMC	2UNINA	3UMU	4WMU	5UMCG	6HULP	7HULF	8 Danone	9AOECS	10Phadia	11Eurospital	12TECHNION	13UO	14CHZ	15HP	16URV	17LMU	Total
Research																		
WP1	0	0	0	0	0	0	0	0	0	0	0	0	0	0	0	0	0	0
WP2	3	0	0	0	0	0	0	0	0	0	0	0	0	0	0	0	0	3
WP3	54	54	0	38	0	38	30	6	0	13	12	42	0	27	38	20	38	410
WP4	0	0	116	0	0	0	0	0	0	7	19	0	0	0	0	0	0	142
WP5	0	48	8	0	48	0	0	0	0	0	0	0	0	0	0	0	0	104
WP6	32	24	0	0	0	0	0	0	0	0	0	0	32	0	0	0	0	88
WP7	0	0	0	9	0	0	0	0	6	0	0	0	0	0	0	0	0	15
Total research	89	126	124	47	48	38	30	6	6	20	31	42	32	27	38	20	38	762
Demonstration																		
WP1	0	0	0	0	0	0	0	0	0	0	0	0	0	0	0	0	0	0
WP2	0	0	0	0	0	0	0	0	0	0	0	0	0	0	0	0	0	0
WP3	0	0	0	0	0	0	0	0	0	0	0	0	0	0	0	0	0	0
WP4	0	0	0	0	0	0	0	0	0	0	0	0	0	0	0	0	0	0
WP5	0	0	0	0	0	0	0	0	0	0	0	0	0	0	0	0	0	0
WP6	0	0	0	0	0	0	0	0	0	0	0	0	0	0	0	0	0	0
WP7	0	0	0	0	0	0	0	0	0	0	0	0	0	0	0	0	0	0
Total	0	0	0	0	0	0	0	0	0	0	0	0	0	0	0	0	0	0
Management																		
WP1	47	1	1	1	1	1	1	1	1	1	1	1	1	1	1	1	1	63
WP2	0	0	0	0	0	0	0	0	0	0	0	0	0	0	0	0	0	0
WP3	0	0	0	0	0	0	0	0	0	0	0	0	0	0	0	0	0	0

WP4	0	0	0	0	0	0	0	0	0	0	0	0	0	0	0	0	0
WP5	0	0	0	0	0	0	0	0	0	0	0	0	0	0	0	0	0
WP6	0	0	0	0	0	0	0	0	0	0	0	0	0	0	0	0	0
WP7	0	0	0	0	0	0	0	0	0	0	0	0	0	0	0	0	0
Total	47	1	1	1	1	1	1	1	1	1	1	1	1	1	1	1	63
TOTAL	136	127	125	48	49	39	31	7	7	21	32	43	33	28	39	21	39	825

8.2 Overall budget for the project (Forms A3.1 & A3.2 from CPFs)
Copy Form A3.1 from CPFs

Contract Preparation Forms

EUROPEAN COMMISSION

6th Framework Programme on Research, Technological Development and Demonstration

Specific Targeted Research or Innovation Project

A3.1

Proposal Number	036383	Proposal Acronym	PREVENTCD

Financial information - whole duration of the project

Participant n°	Organisation short name	Cost model used	Estimated eligible costs and requested EC contribution (whole duration of the project)		Costs and EC contribution per type of activities: RTD or innovation related activities (1)	Demonstration activities (2)	Consortium Management activities (3)	Total (4)=(1)+(2)+(3)	Total receipts
1	LUMC	AC	Eligible costs	Direct Costs (a)	430.257,00	,00	214.374,00	644.631,00	
				of which subcontracting				,00	
				Indirect costs (b)	86.051,00		42.874,00	128.925,00	
				Total eligible costs (a)+(b)	516.308,00	,00	257.248,00	773.556,00	
			Requested EC contribution		516.308,00	,00	257.248,00	773.556,00	
2	UNINA	AC	Eligible costs	Direct Costs (a)	323.883,00	,00	,00	323.883,00	
				of which subcontracting				,00	
				Indirect costs (b)	64.777,00		,00	64.777,00	
				Total eligible costs (a)+(b)	388.660,00	,00	,00	388.660,00	
			Requested EC contribution		388.660,00	,00	,00	388.660,00	
3	UMU	AC	Eligible costs	Direct Costs (a)	568.878,00	,00	,00	568.878,00	
				of which subcontracting				,00	
				Indirect costs (b)	113.775,00		,00	113.775,00	
				Total eligible costs (a)+(b)	682.653,00	,00	,00	682.653,00	
			Requested EC contribution		682.653,00	,00	,00	682.653,00	
4	WMU	AC	Eligible costs	Direct Costs (a)	87.219,00	,00	,00	87.219,00	
				of which subcontracting				,00	
				Indirect costs (b)	17.444,00		,00	17.444,00	
				Total eligible costs (a)+(b)	104.663,00	,00	,00	104.663,00	
			Requested EC contribution		104.663,00	,00	,00	104.663,00	
5	UMCG	AC	Eligible costs	Direct Costs (a)	298.179,00	,00	,00	298.179,00	
				of which subcontracting				,00	
				Indirect costs (b)	59.636,00		,00	59.636,00	
				Total eligible costs (a)+(b)	357.815,00	,00	,00	357.815,00	
			Requested EC contribution		357.815,00	,00	,00	357.815,00	
6	HULP	AC	Eligible costs	Direct Costs (a)	121.828,00	,00	,00	121.828,00	
				of which subcontracting				,00	
				Indirect costs (b)	24.366,00		,00	24.366,00	
				Total eligible costs (a)+(b)	146.194,00	,00	,00	146.194,00	
			Requested EC contribution		146.194,00	,00	,00	146.194,00	
7	HULF	AC	Eligible costs	Direct Costs (a)	98.167,00	,00	,00	98.167,00	
				of which subcontracting				,00	
				Indirect costs (b)	19.633,00		,00	19.633,00	
				Total eligible costs (a)+(b)	117.800,00	,00	,00	117.800,00	
			Requested EC contribution		117.800,00	,00	,00	117.800,00	
8	Danone	FC	Eligible costs	Direct Costs (a)	103.745,00	,00	,00	103.745,00	
				of which subcontracting	[illegible]0.000,00			60.000,00	
				Indirect costs (b)	20.749,00		,00	20.749,00	
				Total eligible costs (a)+(b)	124.494,00	,00	,00	124.494,00	
			Requested EC contribution		62.247,00	,00	,00	62.247,00	

9	AOECS	AC	Eligible costs	Direct Costs (a)	14.351,00	,00	,00	14.351,00	
				of which subcontracting				,00	
				Indirect costs (b)	2.870,00		,00	2.870,00	
				Total eligible costs (a)+(b)	17.221,00	,00	,00	17.221,00	
			Requested EC contribution		17.221,00	,00	,00	17.221,00	
10	Phadia	FC	Eligible costs	Direct Costs (a)	217.949,00	,00	,00	217.949,00	
				of which subcontracting				,00	
				Indirect costs (b)	43.724,00		,00	43.724,00	
				Total eligible costs (a)+(b)	261.673,00	,00	,00	261.673,00	
			Requested EC contribution		108.020,00	,00	,00	108.020,00	
11	Eurospital	FC	Eligible costs	Direct Costs (a)	349.950,00	,00	,00	349.950,00	
				of which subcontracting				,00	
				Indirect costs (b)	44.794,00		,00	44.794,00	
				Total eligible costs (a)+(b)	394.744,00	,00	,00	394.744,00	
			Requested EC contribution		197.372,00	,00	,00	197.372,00	
12	TECHNION	AC	Eligible costs	Direct Costs (a)	101.077,00	,00	,00	101.077,00	
				of which subcontracting				,00	
				Indirect costs (b)	20.216,00		,00	20.216,00	
				Total eligible costs (a)+(b)	121.293,00	,00	,00	121.293,00	
			Requested EC contribution		121.293,00	,00	,00	121.293,00	
13	UO	AC	Eligible costs	Direct Costs (a)	176.370,00	,00	,00	176.370,00	
				of which subcontracting				,00	
				Indirect costs (b)	35.274,00		,00	35.274,00	
				Total eligible costs (a)+(b)	211.644,00	,00	,00	211.644,00	
			Requested EC contribution		211.644,00	,00	,00	211.644,00	
14	CHZ	AC	Eligible costs	Direct Costs (a)	14.250,00	,00	,00	14.250,00	
				of which subcontracting				,00	
				Indirect costs (b)	2.850,00		,00	2.850,00	
				Total eligible costs (a)+(b)	17.100,00	,00	,00	17.100,00	
			Requested EC contribution		17.100,00	,00	,00	17.100,00	
15	HP	AC	Eligible costs	Direct Costs (a)	83.970,00			83.970,00	
				of which subcontracting				,00	
				Indirect costs (b)	16.794,00			16.794,00	
				Total eligible costs (a)+(b)	100.764,00	,00	,00	100.764,00	
			Requested EC contribution		100.764,00			100.764,00	
16	URV	AC	Eligible costs	Direct Costs (a)	84.532,00			84.532,00	
				of which subcontracting				,00	
				Indirect costs (b)	16.906,00			16.906,00	
				Total eligible costs (a)+(b)	101.438,00	,00	,00	101.438,00	
			Requested EC contribution		101.438,00			101.438,00	
17	LMU	AC	Eligible costs	Direct Costs (a)	138.800,00			138.800,00	
				of which subcontracting				,00	
				Indirect costs (b)	27.760,00			27.760,00	
				Total eligible costs (a)+(b)	166.560,00	,00	,00	166.560,00	
			Requested EC contribution		166.560,00			166.560,00	
TOTAL			Eligible costs		3.785.391,00	,00	257.248,00	4.042.639,00	,00
			Requested EC contribution		3.417.752,00	,00	257.248,00	3.675.000,00	

Copy Form A3.2 from CPF's

Estimated breakdown of the EC contribution per reporting period				
Reporting Periods	Start month	End month	Estimated Grant to the budget	
				Of which first 6 months
Reporting Period 1	1	12	816.697	
Reporting Period 2	13	24	1.241.955	413.985
Reporting Period 3	25	36	899.580	449.790
Reporting	37	48	366.768	183.384

Period 4				
Reporting period 5	49	60	350.000	175.000
Total			3.675.000	

8.3 Management level description of resources and budget.

Partner	Management Activity	Research Activity						Indirect Costs	Total	EU contribution
	wp 1	wp 2	wp 3	wp 4	wp 5	wp 6	wp 7			
LUMC	214.374	29.190	262.317	0	0	138.750	0	128.926	773.557	773.557
UNINA	0	0	138.583	0	91.500	93.800	0	64.777	388.660	388.660
UMU	0	0	0	546.761	22.117	0	0	113.775	682.653	682.653
WMU	0	0	66.719	0	0	0	20.500	17.444	104.663	104.663
UMCG	0	0	0	0	298.179	0	0	59.636	357.815	357.815
HULP	0	0	121.828	0	0	0	0	24.366	146.194	146.194
HULF	0	0	98.167	0	0	0	0	19.633	117.800	117.800
Danone	0	0	103.745	0	0	0	0	20.749	124.494	62.247
AOECS	0	0	0	0	0	0	14.351	2.870	17.221	17.221
Phadia	0	0	103.111	64.883	0	0	0	48.046	216.040	108.020
Eurospital	0	0	182.600	167.350	0	0	0	44.794	394.744	197.372
TECHNION	0	0	101.078	0	0	0	0	20.216	121.293	121.293
UO	0	0	0	0	0	176.370	0	35.274	211.644	211.644
CHZ	0	0	14.250	0	0	0	0	2.850	17.100	17.100
HP	0	0	83.970	0	0	0	0	16.794	100.764	100.764
URV	0	0	84.532	0	0	0	0	16.906	101.438	101.438
LMU	0	0	138.800	0	0	0	0	27.760	166.560	166.560
Total	214.374	29.190	1.499.700	778.994	411.795	408.920	34.851	664.816	4.042.639	3.675.000

Twice a year a progress meeting will be organized in which all the partners (approximately 30 persons) are expected to participate. The meetings are expected to have place on months: 3, 9,15,21,27,33,39,47,53 and 60. The place of the meetings will be the working place of one of the European partners. Eight of the management meetings will take place at the same place, before the progress meeting. The rest of the management meetings will take place as telephonic conferences.

Justification of the requested budget for travels and meetings

Budget for meetings (10 total expected)

Budget for travel/transport

Travel expenses for Meetings:	(10 meetings in total – 10.000,00 per meeting)	100.000,00
**Per Meeting:**	Accomodation	
	30 people – 2 nights	13.000,00
	Rooms hire	1.500,00
	Coffee breaks	381,75
	Audio visual	381,75
	Administration	700,00
	Dinners	3.000,00
	**Subtotal**	**18.963,50**
**Total**	**10 meetings**	**189.635,00**
Travel to patients/transport samples		29.959,00
**Total**		**129.959,00**

9. Ethical issues

9.1. Declaration on the exclusion of particular research activities.

Ethical issues in EU research proposals - checklist

**The proposal nr. 036383 PREVENTCD involves**	**Yes**	**No**	**Uncertain**
**· Research on human beings**	Yes		
Persons not able to give consent		No	
Children	Yes		
Adult healthy volunteers		No	
**· Human biological samples**	Yes		
Human foetal tissue/cells		No	
Human embryonic stem cells		No	
Use of banked or isolated human embryonic stem (hES) cells in culture, human foetuses, and human foetal tissue		No	
**· Human embryos**		No	
**· Human genetic information**	Yes		
Research activity intended to modify the genetic heritage of human beings which could make such changes heritable		No	
**. Human clones**		No	
**· Other personal data**	Yes		
Sensitive data about health, sexual lifestyle, ethnicity, political opinion, religious or philosophical conviction	Yes		
**· Animals (any species)**		No	
Non- human primates			
Transgenic small laboratory animals			
Transgenic farm animals			
Cloning of farm animals			
**· Research involving developing countries (e.g. clinical trials, use of human and animal genetic resources...)**		No	
**· Dual use**		No	

PREVENTCD confirms that the proposed research does not involve:

- Research activity aimed at human cloning for reproductive purposes.
- Research activity intended to modify the genetic heritage of human beings, which could make such changes heritable.

- Research activity intended to create human embryos solely for the purpose of research or for the purpose of stem cell procurement, including by means of somatic cell nuclear transfer.

9.2. Conformity with fundamental ethic principles as well as national and EU legislations.

9.2.a. Identification of countries where specific research will be carried out*

Country	Participant org. short name
The Netherlands	LUMC
	UMCG
	Danone
Sweden	UMU
Italy	UNINA
	Eurospital
Poland	WMU
Spain	HULP
	HULF
	URV
Belgium	AOECS
Germany	Phadia
	LMU
Norway	UO
Israel	TECHNION
Poland	UO
Croatia	CHZ
Hungary	HP

9.2.b. Relevant legislations and international texts which will be followed.

The fundamentals of clinical research are contained within the Declaration of Helsinki. PREVENTCD wholly and unreservedly supports and actively promotes the principles laid down by this key document. The study will be conducted in compliance with the

rules of the ICH-GCP (International Conference on Harmonization for Good Clinical Practice and with the Council of Europe Convention on Human Rights and Biomedicine (Additional Protocols on Biomedical Research – 2005), since all the European participating countries have signed the Convention (Source : Treaty Office on http://conventions.coe.int/). In addition, there is clear legislation in Israel (2001) concerning the strict prohibition of economic, employment, social or insurance discrimination on the grounds of genetics.

Important EU directives, which have a significant impact on the work of our project, are:

- The Charter of fundamental Rights of the EU, conventions of the Council of Europe on human rights and biomedicine, and the UNESCO Declaration on the human genome.
- Directive 95/46/EC of the European Parliament and of the Council of 24 October 1995 on the protection of individuals with regard to the processing of personal data and on the free movement of such data.

In addition, each partner will comply with its relevant national legislation. A list of applicable laws and regulatory texts follows for each country involved.

<u>The Netherlands</u>:

- Wet Medisch–wetenschappelijk Onderzoek met mensen (WMO), 1 december 1999.
- Implementation of EU directive 2001/20/EC into national legislation in The Netherlands is aimed by changing the WMO and the WOG.
- Wet op de Geneeskundige Behandelingsovereenkomst (WGBO), 1 april 1995.
- Wet Bescherming Persoonsgegevens (WBP), 6 juli 2000.
- Wet veiligheid en kwaliteit lichaamsmateriaal (WVKL), 6 februari 2003.

<u>Sweden</u>:

- The Ministry of Education and Cultural Affairs; The Act concerning the Ethical Review of Research Involving Humans (2003:460), issued June 5, 2003, and with the following regulations a) Statute (2003:615) concerning the ethical vetting of research involving humans, issued October 9, 2003, b) Statute (2003:616) containing instructions for regional ethical review boards, issued October 9, 2003, and c) Statute

(2003:617) containing instructions for the Central Ethical Review Boards, issued October 9, 2003.

- Ministry of Health and Social Affairs; The Biobanks in Medical Care Act (2002:297), issued May 23, 2002 (Biobankslagen) with the following regulation; Statute (2002:746) concerning biobanks in health care and medical care, issued February 10, 2002.
- Ministry of Justice; The Personal Data Act (1998; 204), issued April 29, 1998 (Personuppgiftslagen, PUL).

Italy:

- Decreto Legislativo n. 211 del 24 giugno 2003: Attuazione della direttiva 2001/20/CE relativa all'applicazione della buona pratica clinica nell'esecuzione delle sperimentazioni cliniche di medicinali per uso clinico.
- Circolare Ministeriale n. 6 del 2 settembre 2002 : Attività dei comitati etici istituiti ai sensi del decreto ministeriale 18 marzo 1998.
- Decreto del Presidente della Repubblica n. 439 del 21 settembre 2001:Regolamento di semplificazione delle procedure per la verifica e il controllo di nuovi sistemi e protocolli terapeutici sperimentali.
- Decreto Legislativo n. 196 del 30 giugno 2003: Codice in materia diprotezione dei dati personali.

Poland

- ROZPORZADZENIE MINISTRA ZDROWIA I OPIEKI SPOLECZNEJ z dnia 11 maja 1999 r. w sprawie szczególowych zasad powolywania i finansowania oraz trybu dzialania komisji bioetycznych. Dz.U. 1999 Nr 47 poz. 480 [Directive of the Ministry of Health of 11 May 1999 on regulations relating to creation, financing and operating activies of bioethical committees. Dz. U. 1999 Nr 47 poz. 480]
- USTAWA z dnia 5 grudnia 1996 r. o zawodach lekarza i lekarza dentysty. Dz.U. 1997 Nr 28 poz. 152. [Directive of 5 December 1996 on the medicine physicians and dental physicians. Dz.U. 1997 Nr 28 poz. 152]
- ROZPORZADZENIE MINISTRA ZDROWIA z dnia 10 grudnia 2002 r. w sprawie okreslenia szczególowych wymagan Dobrej Praktyki Klinicznej. Dz. U. 02 nr 221

poz.1864 [Directive of the Ministry of Health of 10 December 2002 on special regulations regarding Good Clinical Practice. Dz. U. 02 nr 221 poz.1864]

- Rozporzadzenie Ministra Finansów z dnia 30 kwietnia 2004 r. w sprawie obowiazkowegoubezpieczenia od odpowiedzialnosci cywilnej badacza i sponsora. Dz. U. 04 nr. 101 poz. 1034 z pózn. zm. [Directive of the Ministry of Finance of 30 April 2004 relating to the obligatory insurance for civil responsibility of the sponsor and investigator. Dz. U. 04 nr. 101 poz. 1034 with later changes]

Spain:

- BOE nº 15 de fecha 17 de Enero de 1996, Ley orgánica 1/96 de 15 de enero de 1996 de PROTECCIÓN JURÍDICA DEL MENOR.

Belgium:

- Loi du 7 mai 2004 relative aux expérimentations sur la personne humaine. Wet van 7 mei 2004 inzake experimenten op de menselijke persoon. Version coordonnée (août 2003) de la loi relative à la protection des données à caractère personnel du 8 décembre 1992. Gecoördineerde versie (augustus 2003) van de wet van 8 december 1992 tot bescherming van de persoonlijke levenssfeer ten opzichte van de verwerking van persoonsgegevens.

Germany:

- Gesetz über Medizinprodukte (Medizinproduktegesetz - MPG) 07-08- 2002 (Medical Devices Law).

Norway:

- Menneskerettsloven av 1999.Biobankloven av 2003. Genteknologiloven av 1993. Personopplysningsloven av 2000.
  All medical research that involves humans (including personal identifiable information and human material) need to be approved by a regional ethics committee. All research that includes handling of personal data, including de-identified information (i.e “avidentifisert”), need to be presented and approved either by an ombud for protection of privacy or the Data Inspectorate.

Israel:

- Governing Regulations of the Israeli Ministry of Health. 1999 Amendment of Public Health Regulations (Medical Trials in Human Subjects) – 1980

- All clinical studies proposals are reviewed, discussed and approved by the local Helsinki Committee (and ratified by the Human Studies Ethics Committee of the Ministry of Health), which carefully abide to the FDA (USA) and ICH-GCP rules.

Croatia:

- Kodeks medicinske etike i deontologije, Narodne novine br. 47, 13.4.2004. (NN 47/04) (Codex of medical ethics and deontology published at Narodne novine No 47, 13th April, 2004).
- Pravilnik o klinickim ispitivanjima i dobroj klinickoj praksi, Narodne novine br 175, 15.10.2003. (NN 175/03) (Regulations on clinical studies and good clinical practice published at Narodne novine No 175, 15th October, 2003).

Hungary:

- 1997. évi CLIV. Törvény az egészségügyről. VIII. Fejezet. Az emberen végzett orvostudományi kutatások. 157-164. §
- 23/2002 EüM rendelet az emberen végzett orvostudományi kutatásokról
- 1997. évi LXXXIII. Törvény a kötelező egészségbiztosítás ellátásairól, egységes szerkezetben a végrehajtásáról szóló 217/1997. (XII. 1.) Korm. Rendelettel
- 1997. évi XLVII. eü. Adatvédelmi törvény.
- Ccomprehensive, EU-conform legislation sumary at: http://ec.europa.eu/research/science-society/pdf/hu_hong_lr.pdf

PREVENTCD confirms that the participants will observe the above-specified relevant national rules concerning research with humans, particularly with children.
PREVENTCD confirms that the project will require the approval of the Independent Ethic Committee that governs clinical studies for each participating study site and that they will inform the Commission whether the local, regional or national ethical approval has been obtained before the research to which it relates is carried out.
The Ethics Committee approval or authorisation of a competent body will be specified as a project deliverable.
The population based multicenter study will only involve Swedish participants, and has already been approved by the relevant Regional Ethical Review Board.

9.3. Description of the sensitive ethical issues of the proposed research

Identification of the potential ethical aspects of the proposed research:

- 9.3.a. Research on human beings, including: Infants (1,000) and children of school age (16,000) not able to give consent.
- 9.3.b. Human biological samples (e.g. umbilical cord blood, venous blood, small bowel biopsy samples).
- 9.3.c. Human genetic data and personal data.

9.3.a. Research on human beings, including: Infants (1,000) and children of school age (16,000) not able to give consent.

9.3.a.I. *Early feeding intervention family study*

A prospective early dietary intervention study of 1000 young children from high-risk families for CD, to assess the possibilities to induce immune tolerance for gluten in genetically predisposed children.

Explanation and justification of the research design

The proposed project must necessarily involve infants, and cannot be performed in any other age group, because it is during infancy that the first exposure to gluten takes place and that the immunological response to this antigen occurs and may be modulated. The only way to find out whether the introduction of small quantities of gluten into the diet of infants during the period of breast-feeding does indeed protect against CD is to conduct a double blind prospective randomised study, as the one we here we herewith propose.

Informed consent:

Informed consent to participate will be obtained from the parents, guardians or other legitimate representative, as it is usual in the case of young children. The AOECS or the local Coeliac Disease Societies will invite their members with CD expecting a newborn (offspring or sibling) during the next 18 months to participate. All parents, guardians or other legitimate representative of the children, interested to collaborate will be given full information and explanations about the trial and asked to sign a written informed consent form (annexes 2 and 3) by the local responsible physician according to the national legislation. Each parent, guardians or other legitimate representative of the children will receive detailed information about the study procedures, the potential risks and benefits

of the intervention, as well as information about the fundamental rights of a patient included in an experimental study. In addition, care will be taken to inform the patient about the potential benefits of the experimental intervention in a manner that avoids an overestimation of these benefits, by explicitly mentioning that the intervention is fully experimental, that it has no proven efficacy, and that no guarantee can be given that it will work against CD.

These information's will be provided by a physician in charge of the trial, and an informed consent document, preliminarily approved by the relevant ethics committee, will be given to the parents, guardians or other legitimate representative of the children. In case of acceptance by the parents, guardians or other legitimate representative of the children, they/he/she will sign the informed consent document, which will be countersigned by the responsible physician. The signed informed consent form will be archived in the Case Report Form (CRF) at the project office, and a copy will remain with the parents, guardians or other legitimate representative of the child. The parents, guardians or other legitimate representative of the children will always give informed consent by their free will. Only after informed consent is signed will the families be enrolled in the study. The family physician will be duly informed and his standing as the family's health provider will be respected – and relied on for his/her cooperation. The participants may retract from their participation at any moment, also without giving their reasons to do so and without any adverse consequences regarding their relationship with their paediatrician/responsible physician. A copy of both the informed consent form and of the parent's information sheet will be submitted to the pertinent Independent Review Board. No financial reward will be given to participating families at all. Only their real expenses (primarily travel costs), which are related to their accurate participation in the studies, shall be reimbursed. The only exception will be to give a small present to the children: this will not be told to the families in advance, to avoid influencing them in their decision to participate or not.

Breast-feeding during at least six months will be STRONGLY encouraged to all the families.

<u>Care for the children and their families during the study</u>

The children and their families will receive frequent and careful medical follow up during

the study (table 1), to ensure their support and their welfare, to ascertain that any undesired effects or symptoms are rapidly detected and that adequate measures are promptly undertaken. In case of health complaints, extra observation will be provided and, if indicated, the participation will be stopped. The main discomfort for the children concerns the blood sampling and the clinical follow up examinations, as detailed in table 1. The risk element involved with this trial concerns that of the performance of small bowel mucosal biopsy in the children with suspected CD. It is again to be stressed that such a biopsy is still the "gold standard" for diagnosing CD, and would be performed only when medically indicated, that is: only in these children with CD antibodies in their serum that indicate gluten sensitivity and are highly suspect for active CD, and NOT just for purpose of the study. Such children would undergo a biopsy also in non-study circumstances. Each centre where the research takes place will make the necessary treatment and infrastructure available to patients who develop trial-related injuries and will provide sufficient insurance to cover subjects for adverse effects directly arising from participation in the trial. All adverse and serious adverse events will be reported according to the regulations cited above.

Implications of the results.

The implications of the proposed study may be important, because its results may contribute to the development of new European guidelines for early infant feeding practices, aimed at the primary prevention of CD and related autoimmune diseases.

In all the countries involved in the project there is clear legislation concerning the strict prohibition of economic, employment, social or insurance discrimination on the grounds of genetics. In the participating countries is illegal for prospective employers to demand genetic testing or to demand to receive the results of genetic testing. Genetic issues may be considered in very limited instances of LIFE insurance. In this context it must be taken into account that testing for HLA in a child of a family with coeliac disease may be regarded as a clinical service, meant to detect a subject that needs to be followed up for an early recognition of the disorder and timely institution of adequate management. In this respect, the HLA testing of infants in this study might constitute an advantage for the participating infants. The information thereby obtained is of a nature that is similar, if not better, than the knowledge that this is an infant of a "coeliac" family.

The results will have no potential ethical adverse implications, concerning the protection of dignity, autonomy, integrity and privacy of persons, and may lead to healthier early feeding recommendations for infants. Finally, the results may lead to a decline in the incidence of CD in the European population.

9.3.a.II. *Population based study*

A study in 16.000 children aged 12 years, born during and after the Swedish epidemic of CD, to assess the late effect of dietary history, concerning gluten exposure and breast-feeding, on the development of CD and related autoimmune phenomena**.**

The study has been approved by the Research Ethics Committee at Umeå University [official register number UmU 04-156 M] (annex 2). There are, however, several ethical considerations involved, which have been carefully considered, and are also briefly discussed below.

Explanation and justification of the research design

The "*Swedish case*" as it is called among scientists internationally, provides a unique opportunity to increase our knowledge concerning CD aetiology. Notably, this study can only be carried out in Sweden, and only for a limited period. The study takes advantage of the recent Swedish epidemic of CD, which has no likeness anywhere else in the world. The epidemic of CD was most likely initiated by changes in infant feeding, which, while considered appropriate at the time, were in retrospect probably quite unfortunate. However, now that the epidemic has occurred it provides a unique opportunity to increase our knowledge concerning CD aetiology. The results may contribute to primary prevention strategies for CD such as changed infant feeding guidelines. Thus, the results may lead to a decline in the incidence of CD in the European population.

- *Possible disadvantages for participants:* Invited children and parents might feel anxiety when reading the folder inviting them to the study (annex 3), as it describes CD as a rather common disease, that a blood sample is needed as the first step to identify currently undiagnosed cases, etc. Families, in which the child has increased values of the serological marker (AtTG) suggesting undiagnosed CD (2%), are also likely to experience some anxiety when waiting for further examinations, thus this time period will be kept as short as possible. The small intestinal biopsy necessary for confirming or

excluding CD is a well-established method for diagnosis and complications are extremely rare, however, during the procedure some discomfort isn't uncommon. Biopsie will only be performed when medically indicated, that is: only in these children with CD antibodies in their serum that indicate gluten sensitivity and are highly suspect for active CD, and NOT just for purpose of the study. Such children would undergo a biopsy also in non-study circumstances. With the CD screening strategy and serological tests that are used a high sensitivity and specificity are combined, thus, almost all CD cases will be identified, and the number of "unnecessary" small intestinal biopsies will be kept to a minimum. A database system is used for the storage of all findings, e.g. results of blood sample analyses and replies in the questionnaires, which might worry some families. However, informed consent is required before inclusion of any child into the study and the database, only a small group of researchers have access to the database, and if the child and/or parents express a wish to end their participation their identity code will immediately be erased.

Advantages for participants: Children with newly diagnosed CD, determined by the study (1%), who follow a gluten-free diet, will gain a lot as their health and quality of life will increase, and the risk for long-term health complications will decrease. All children with a normal blood-sample result, and their families, will most likely experience a relief that CD has been excluded.

Risk-benefit evaluation for participants:

The participating children are expected to fall into one of the three different groups with respect to risk-benefit evaluation:

A) AtTG with a normal value. The child and parents might experience some anxiety when reading the folder with information about the study and when the child takes part in the blood sampling. These children will incur minimal risk/burden and gain the knowledge that CD can be excluded for them. Risk and benefit are considered balancing each other.

B) AtTG is elevated and the follow-up confirms the CD diagnosis. These children will benefit considerably from the study as an early diagnosis of a chronic and treatable disease (CD) should allow improved future health and well being for all years coming.

C) AtTG is elevated but CD is excluded by further examinations. These children will – without any apparent benefit to themselves –undergo a small bowel biopsy in addition to the minimal risks/burdens experienced by others (blood sample, anxiety). These children and parents are "unnecessarily" exposed to anxiety. Also the small intestinal biopsy has to be done, which causes some discomfort, but no medical risks. The burden may be considered larger than the benefits.

Informed consent

The population based study invites children who are 12-years old, in the sixth grade of basic school, to participate in a screening for CD. These children are at an age approaching adolescence. They have the right to be informed about the study, and their wish to participate or not should be taken into account. However, their parents or other legitimate guardian should make the final decision after having informed and discussed it with the child. Informed consent from both the child *and* their parents or other legitimate guardian is required before participation is accepted. Only children and parents who give informed consent will be approached regarding participation. Information and an invitation to participate are sent to the child's home through their school (annex 4). This folder with information has several parts; one for the parents (Dear Parents!), one for the child (Greetings to you in the 6th grade!), and another to read and discuss jointly. Information is given about the study procedures, the potential risks and benefits of the intervention, as well as information about the fundamental rights of any person taking part in a research study. The informed consent will always be given by free will. The written information will be kept by the child and their parents, while the signed consent form will be archived at the project office. The participants will not receive any payment or other benefit for their participation, except jus, fruit and/or a small biscuit at the blood sample occasion, and a small present (value 2 Euro), which is not notified beforehand as it should not influence the decision to participate or not. The participants are clearly informed that they may retract from participation at any moment, also without giving their reasons to do so and without any consequences regarding their relationship with their school, school health service or paediatrician.

Care for the children and their families during the study

The children and their parents invited to the study are met respectfully, irrespective of their decision to participate or not. Blood sampling of the children is performed only after informed consent is given and by experienced paediatric nurses working jointly with the nurse in the school health service, who is a person the children are familiar with. The children are offered a topical anesthetic preparation before sampling and may withdraw from participation any time during the procedure. The time period between the blood sampling and reporting the results of the AtTG analyses to the parents is kept as short as possible, to minimize any anxiety. The parents of children not suspected to have CD are informed by a letter, including information on how the local responsible paediatric gastroenterologist can be reached if they have any questions. An experienced paediatric gastroenterologist takes care of informing the parents of children with suspected CD; first by telephone, immediately thereafter with a letter, and shortly after this in a doctors visit arranged for the child and parents at the paediatric unit. Suspected CD can only be verified by a small intestinal biopsy, which is a standard procedure within paediatric

Possible implications of the results

By the PREVENTCD project, combining the family intervention approach and the population based survey, we will be able to clarify whether *primary prevention of CD* is possible by means of a change in infant feeding practices, or whether it is merely the symptoms and thereby the chance of being diagnosed that are influenced. By the population based screening at 12 years of age we will also determine whether infant feeding practices delay the development of CD enteropathy, or if the reduced risk also remains later in life. We will also evaluate whether the *risk for autoimmunity can be reduced* in CD and non-CD subjects by a reduction in the amount of gluten ingested during infancy. Moreover, we can determine whether CD subjects will have fewer additional autoimmune diseases if their CD is detected and treated early in life. Thus, PREVENTCD may contribute to the *development of new European guidelines for early feeding*, aimed at the primary prevention of CD and related autoimmune diseases. It may lead to *healthier* early feeding *recommendations for infants*. Finally, the results may lead to a *decline in the incidence of CD in the European population.*

Identification of four possible problems concerning compliance with the Convention on Human Rights and Biomedicine Article 17 – Protection of persons not able to consent to research.

Executive summary:

Problem I: Gluten will be given to the infants at the age of 4-6 months, which is against the current recommendations of WHO and ESPGHAN.

The project is - in principal – strongly backing the general principle of breast feeding, which is the only generally accepted prevention strategy with respect to CD. This leading principle is clearly in line with current recommendations.

The current WHO and ESPGHAN recommendations refer to the general population, but not to the high risk group predisposed to CD as within the family study of this project. For this sub-population, there are no guidelines at all.

The strong recommendation of ESPGHAN not to feed gluten before month 4 will strictly be followed. The current advice for the age between months 4 and 5 (and later) is less strict in the current recommendations.

Gluten is given only in the lowest possible amount expected to have an effect on the immune system (max. 100 mg), which is much lower (aprox. 3%) and not comparable to the amount of gluten introduced as complementary food after weaning (grams).

There are several studies on epidemiology and basic immunological principles supporting the “window of opportunity concept”. Scientific studies by the Swedish partner of PREVENTCD exploring in detail the "CD epidemic" in Sweden, strongly support introduction of gluten during the 4th to the 5th month of age not to increase the risk for CD, and possibly also to decrease the risk. Notably, the CD epidemic started in 1983 when gluten introduction was postponed from month 4 to 6 by changed national recommendations to parents, and also the baby food producers taking out existing gluten from products aimed for this age-group. Moreover, the epidemic subsided when gluten introduction in 1996 was once again “allowed” from 4 months of age.

Problem II: the bowel biopsies in the intervention study go beyond the minimum burden/risk.

A small bowel biopsy will only be performed on children participating in the study if there is a medical indication to do so. A small bowel biopsy is the only tool to provide a diagnosis of CD. The biopsies are part of the normal clinical diagnostic routine and are not exclusively used for research purposes in this study. Early identification of CD in high risk populations is an accepted recommendation and should be offered to these high risk children also if they do not participate in the project. This is a crucial aspect, since a diagnostically/medically indicated intervention is not an issue of the Convention. For individuals with elevated immunological blood markers, a biopsy is strongly recommended and medically indicated.There are no limiting recommendations with respect to the children's age for diagnostic biopsies. Because of their high risk for CD 8-10 % (n = 80-100) of the children would undergo a biopsy even without the study.

Problem III: The dense blood sampling scheme for infants in the intervention study was regarded to be beyond minimum burden/risk.

The ethically justified blood sampling scheme should not only avoid and limit burden, but should also be of high diagnostic value for the patients and to allow valid scientific results. The consortium will reduce the number of blood samples to seven blood punctures in total (Table 1) and the quantity of blood needed to 5 ml. The number and timing of the blood punctures is such that it will enable to monitor the short and long-time effects of the intervention. At the age of 3 years 10 ml blood will be obtained from the children to allow for determination of additional (auto)immune phenomena related to the development of CD.

Problem IV: The Swedish population study imposes biopsies on an estimated subgroup of 160 children without direct benefit to them.

Biopsies in the population study will only be performed when medically indicated, which means only in children with CD antibodies in their serum implying a high suspicion of active CD. The first screening round, funded mainly by Swedish government research agencies, has revealed that undiagnosed and, thus, untreated CD is more common than expected (aprox. 2%). A second screening round might therefore be considered as a benefit to all the children involved since: 1) 2% of children will benefit considerably from the study as an early diagnosis of CD should allow improved future health; 2)

97.6% of them will incur minimal risk/burden and gain the knowledge that CD can be excluded for them and 3) the small group (aprox. 0.4%, 58 children) with signs of gluten sensitivity, as evidenced by the presence of CD antibodies in serum, but without evident small intestinal lesions, will benefit from the planned follow-up by a paediatric gastroenterologist. Preliminary results already indicate that most of these children already have health problems as in undiagnosed CD, and will develop evident CD within short.

National/local ethical approval of the studies

The second round of the Swedish population study has already been approved by the regional ethical committee. For the family intervention study the approval of the local ethical committees is a necessary step before the research can start in any of the countries.

A more detailed explanation of the reasons involved in the Executive Summary is exposed below.

Children with a genetic predisposition for CD will be exposed to small amounts of gluten at ages 4-6 months, contrary to current dietary advice.

This point represents indeed a possible ethical concern, because the current dietary advice in most countries is to not introduce gluten into the diet before the age of six months. This is based on recommendation issued by the European Society for Paediatric Gastroenterology, Hepatology and Nutrition (ESPGHAN 1982) and by the World Health Organisation (WHO 2003). ESPGHAN states that: "*Gluten containing foods should not be introduced before 4 months of age. Even further postponement until the age of 6 months may be advisable*". Moreover, WHO recommends exclusive breast-feeding for six months, with introduction of complementary foods and continued breast-feeding thereafter. However, it is necessary to clarify the following aspects that should be taken into account:

- It is important to emphasize that the current advice with respect to the introduction of gluten into the diet of young children is meant for the general population, but there is not current dietary advice for children genetically predisposed for CD.
- PREVENTCD concerns the administration of very small amounts of gluten (100mg)

after the age of 4 months, which may be interpreted as in accordance with the ESPGHAN recommendations. PREVENTCD is fully aware that the project, where plans to give a subgroup of infants 100 mg gluten from the 4th to the 6th month of age, somewhat contradicts the WHO guidelines recommending exclusive breast-feeding for six months with introduction of complementary foods and continued breast-feeding thereafter. However, it should be noted that PREVENTCD strongly encourages breast-feeding for at least 6 months and that we do not propose weaning at the age of 4 months, but to give very small amounts of gluten.

CD is mainly a genetically-based immunologically-mediated disorder, whose major feature concerns a destructive immunologic/inflammatory process of the upper small intestine in genetically predisposed individuals who consume gluten. According to the immunological principles regarding treatment protocols whereby tolerance to an offending antigen can be induced by very small quantities of this same antigen, PREVENTCD considers that these principles can be employed in order to produce a benefit on the health of the coeliac-prone infant: that is, to render him/her tolerant to gluten by administration of very small quantities of this antigen, to avert – or at least minimize – the manifestation of frank CD. The dose of 100 mg, which is less than 3% of the amount of gluten introduced in the diet at weaning time (Hopman, 2007) is the lowest that can be expected to have an effect on the immune system (Catassi 1993), which is a requirement for immunomodulatory effects and for the possible induction of tolerance to avoid the development of CD. PREVENTCD does not propose to advance the current introduction of gluten into the diet of the children prone to CD: that is the administration of (relatively) large quantities of gluten at once, but to administer 100mg gluten per day, an amount not comparable to those usually given at the weaning time. Therefore, it should not be considered "gluten introduction", but rather a gluten tolerance-inducing procedure. Notably, the current dietary advice with respect to gluten introduction to infants is not evidence-based. The only international guideline available is the one mentioned above by ESPGHAN, and the studies behind this recommendation do not at all fulfil today's requirements for valid scientific evidence. This is important to consider because, since this recommendation was launched, the incidence of CD in Europe has not decreased, but increased (Auricchio

1992; Catassi 2001, Ivarsson 2000, Steens 2005) suggesting that the current dietary advice does not necessarily represent a benefit for the participating children.

- The available scientific evidence of today shows that gluten introduction within the interval of 4-6 months of age is not a risk factor for the development of CD, since none of the studies performed on this topic have ever identified the age of the infant at introduction of gluten as an independent risk factor for developing the disease (Auricchio 1983; Greco 1988, Cataldo 1991; Falth-Magnusson 1996; Ascher 1997, Peters 2001, Ivarsson 2002). Moreover, recent research has suggested that children genetically predisposed for CD might actually benefit from introduction of dietary gluten within the age interval of 4-6 months. This age interval is suggested to represent a "window of opportunity", i.e. introduction of gluten within this age interval might reduce the risk for development of autoimmune phenomena as diabetes and CD (Ziegler 2003, Norris 2005).
- The experience with the Swedish "CD epidemic", and studies that followed thereafter, strongly support that introduction of gluten during the 4th to the 5th month of age does not increase the risk for CD, and possibly also to decrease the risk. Notably, the CD epidemic started in 1983 when gluten introduction was postponed from month 4 to 6 by changed national recommendations to parents, and also the baby food producers taking out existing gluten from products aimed for this age-group. Moreover, the epidemic subsided when gluten introduction in 1996 was once again “allowed” from 4 months of age. However, scientific studies by the Swedish partner of PREVENTCD exploring the epidemic in detail suggest that the true causal factors were whether breast-feeding was ongoing or not while gluten was introduced and also the amount of gluten then given (Ivarsson 200, 2002, 2005). Thus, when introduction of gluten in 1983 was postponed it also implied that more infants had ended breast feeding, and that gluten was introduced by larger amounts. When gluten once again in 1996 was given from the age of 4 months, more of the infants were still breast-fed and gluten was introduced by smaller amounts. Thus, the Swedish studies strongly support to introduce gluten in small amounts and while breast-feeding is ongoing, as proposed in PREVENTCD.

For the above named reasons the proposed dietary intervention may be considered in the category of "not more than minimal risk".

As stated above there is strong scientific evidence suggesting that introduction of gluten in the age interval of 4-6 months is not a risk factor to develop CD, and it might even be beneficial to seize this "window of opportunity" for developing oral tolerance towards gluten. Also, by introducing very small quantities of gluten earlier than commonly done today, a larger proportion of infants will still be breast feeding and this is likely to increase there capability to develop oral tolerance to gluten and avoid CD (Ivarsson 2002, Ivarsson 2005, Akobeng 2006).

Notably, studies performed by the Swedish partner of our consortium, Anneli Ivarsson, strongly indicate that the introduction of small amounts of gluten during the period of breast-feeding may indeed prevent the development of as much as 50% of the cases of CD (Ivarsson 2000, 2002, 2005). In these studies the consequences of early exposure to gluten were also explored in detail with conclusions emphasising the importance of introducing gluten to infants in small amounts. A recent systematic review and a meta-analysis of observational studies published between 1966 and 2004 (Akobeng 2006), also concludes that breast-feeding during the introduction of dietary gluten, and increased duration of breast-feeding are associated with a reduced risk of developing CD. Because the majority of the European infants are not breast-fed after the age of 6 months, when current (not evidence based) advice is to introduce gluten, the European populations is presently not benefiting from the possible tolerogenic effect that breast milk might have on gluten, but would have to a larger extent if gluten introduction was done earlier in life, e.g. between 4-6 months of age as suggested in PREVENTCD.

The bowel biopsies in the intervention study go beyond the minimum burden/risk.

- A small bowel biopsy is the only tool to provide a diagnosis of CD. The biopsies are part of the normal clinical diagnostic routine and are not exclusively used for research purposes in this study. Early identification of CD in high risk populations is an accepted recommendation and should be offered to these high risk children also if they do not participate in the project. This is a crucial aspect, since a diagnostically/medically indicated intervention is not an issue of the Convention. The

small intestinal biopsy necessary for confirming or excluding CD is a well-established method for diagnosis and complications are extremely rare, however, during the procedure some discomfort isn't uncommon. In this context we would like to clarify the following aspects:It is important to emphasize that the small bowel biopsies in PREVENTCD will be performed only when medically indicated, that is: only in these children with CD antibodies in their serum that indicate gluten sensitivity and are highly suspect for active CD, and NOT just for purpose of the study. Such children would undergo a biopsy also in non-study circumstances.

In addition PREVENTCD wishes to make clear that the number of expected small bowel biopsies (70) in the dietary intervention study (1000 children prone to CD) is not increased because of the intervention. The consortium hopes even to reduce the number of necessary biopsies to only 70 instead of 100 (50 from the sub-group without intervention plus about 20 from the sub-group with an intervention); which means a reduction of CD by 50 % after intervention. This figure is based on evidence from the Swedish study on the "CD epidemic" in Sweden. Following the current dietary advice the frequency of CD expected in this high risk population is 8-10%: thus, without the dietary intervention 80-100 children are expected to undergo a diagnostic small bowel biopsy for CD. Based on the hypothesis that with the proposed dietary intervention we will be able to prevent around 50% of the disease occurrence and including a marginal increase, we have calculated for 70 biopsies during the intervention study.

- This project, by virtue of careful scrutiny and follow-up of children with high risk for CD, is likely to detect CD BEFORE the start of its clinical manifestations, allowing the prompt institution of a gluten-free diet and preventing the adverse long-term health implications of active CD. These children will have the genuine benefit of the detection of CD BEFORE it becomes clinically evident. "Clinically evident CD" is a stage that already carries an adverse impact on the child's growth, development, and health.
- Furthermore, there will be a direct health benefit for all the participating children since: 1) In the immunologically identified and diagnosed sub-group of individuals, the clinical manifestation will be detected at an early stage allowing the prompt

institution of a gluten-free diet; 2) the children who, thanks to the study, can be classified as early as possible as not being prone to CD, will be spared the anxiety of vigilance and expectation toward the development of CD and 3) the small group of children with signs of gluten sensitivity, as evidenced by the presence of CD antibodies in serum, but without evident small intestinal lesions, will benefit from the planned follow-up by a paediatric gastroenterologist. Preliminary results already indicate that most of these children will develop CD later in life. It is important to take into account that the positive predictive value of blood testing is very high, so that there is only a small chance for "false positive" cases; in particular as this study is working with a high risk cohort.

- The dietary intervention study of PREVENTCD deals with families where at least one member has CD. They are conscious that their infant has a risk of 10% to develop CD. These families have extensive knowledge of the disease, its problems, complications and management. These families, by experience, will be able to judge if the burden from obtaining blood samples for the purposes indicated in PREVENTCD is in balance to the burden derived from the work-up that their infant would undergo whenever he/she has a possible diarrhoeal bout.

In addition, and to diminish the burden of PREVENTCD as much as possible we make clear the following aspects:

- Because to our knowledge there are not guidelines about the frequency of blood sampling in young children ethically acceptable in research settings, we have reduced the number of blood sampling (7) and the amount of blood to be obtained (5 ml) to the minimum acceptable to obtain scientific valid results. The amount of blood is sufficient to allow for the screening of CD-specific antibodies, phenotypic characterization of 20 markers indicating lymphocyte activation and regulatory T cell induction, and monitoring for the occurrence of gluten-specific T cell responses typical for CD. At the age of 3 years 10ml blood will be obtained to allow for determination of additional (auto) immune phenomena related to CD.
- The pain caused by venapunctures will be diminished as much as possible by previous administration of a local anaesthetic crème.
- The “frequent controls” consist of fairly routine infant health care evaluations

(weight, height, well-being, development, etc).

- The anthropometric data will be, as much as possible, obtained from the files of the Well-baby Clinics attended frequently by most of the babies from the participating countries.
- The data necessary to fill in the standardized forms on Clinical History and Food Questionnaire will be obtained as much as possible by telephonic interview with the parents or guardians.
- If the organisation of the postal system in the participating country allows it, the mothers will be helped to send their samples of breast milk at months 1,2,3, and 6, by post, following written instruction and using "ready to use" material provided by the project.

For the above named reasons the proposed dietary intervention may be considered in the category of "potential to produce health benefits" and "minimal burden".

The Swedish population study imposes biopsies on an estimated subgroup of 160 children without direct benefit to them.

The population based study invites children who are 12-years old, in the sixth grade of basic school, to participate in a screening for CD. These children are in an age approaching adolescence. They have the right to be informed about the study, and their wish to participate or not should be taken into account. However, their parents or other legitimate guardian should of course make the final decision after having informed and discussed it with the child. Informed consent FROM BOTH the child *and* their parents or other legitimate guardian is required before participation is accepted.

Based on the first screening round for CD in *2005-2006* (children born 1993), performed *outside* the PREVENTCD project, we now have valuable information that can be of use in the ethical review of the second screening round planned in *2009-2010* (born 1997) *within* PREVENTCD. The repeated screening round is justified by approaching two birth cohorts with considerable differences both in infant feeding exposure and incidence rate of symptomatic CD.

In our experience from the invitation of 10,000 children during the school year 2005-2006, outside the PREVENTCD project, the following three scenarios were of relevance:

1) In about 9800 (98%) of the families child and guardian agreed on whether or not to participate, and then it is evident how to proceed.
2) For about 150 children (1.5%) an informed consent to participate was received, however, when it was time for blood sampling the child hesitated, the process was halted and the guardian informed about the non-participation.
3) About 50 children (0.5%) expressed an interest to participate but the guardian hadn't signed the consent form, and when the guardian was approached with this information they mostly consented (47/50).

The first CD screening round in 2005-2006 has also provided important revelations about how the children experience participating in such a mass screening. A number of weeks after the screening, children in 15 classes were asked to reflect on and describe their experience with the screening in short narratives during class. The feedback clearly reveals that most children experience a sense of accomplishment, pride, and excitement, and although many felt some worry before the blood sampling afterwards they stated that they experienced less pain then expected.
It is important to emphasize here again that the small bowel biopsies in PREVENTCD will be performed only when medically indicated, that is: only in children with CD antibodies in their serum indicating gluten sensitivity and highly suspected for active disease, and NOT just for purpose of the study. Such children would have a biopsy also in non-study circumstances.
The participating children are expected to fall into the following three different groups with respect to risk-benefit evaluation:
A) 98% Children who will incur minimal risk/burden and gain the knowledge that CD can be excluded for them.
B) 1% Children who will benefit considerably from the study as an early diagnosis of CD should allow improved future health.
C) 1% Children who will undergo a small bowel biopsy because elevated serological markers strongly indicating CD, but without evident small bowel lessions.
Preliminary findings from the first screening round for CD in *2005-2006*, performed *outside* the PREVENTCD project, have shown the following: So far out of the 10,000

children invited, participation has been accepted by 7,400 families (74%), instead of the expected 8,000. Previously diagnosed CD was reported for 79 children, i.e. a prevalence of 11/1000. Additionally, 192 children fulfilled serological criteria for suspected CD, corresponding to 26/1000, which is a higher proportion than expected. Most of these children have already been seen by a paediatric gastroenterologist and small intestinal biopsies are underway according to well-established and accepted clinical routines. Interestingly, out of the 55 biopsies so far performed, as many as 47 (85%) reveal villous atrophy, thus confirming the suspicion of active CD. Thus, this first screening round (2005-2006) has revealed that undiagnosed, and thereby also untreated CD, is more common than expected and as frequent as about 2% (group B). Notably, the group of children with elevated serological markers but without evident CD (group C) was less than half of expected and as infrequent as 0,4% (29/7400). In addition, most of them report considerable health problems commonly seen in CD .
A second screening round might therefore be considered as a benefit to all children involved since: 1) 2% of children will benefit considerably from the study as an early diagnosis of CD should allow improved future health (group A); 2) 97.6% of them will incur minimal risk/burden and gain the knowledge that CD can be excluded for them (group B) and 3) the small group (aprox. 0.4%, 58 children) with signs of gluten sensitivity, as evidenced by the presence of CD antibodies in serum, but without evident small intestinal lesions (group C), will benefit from the planned follow-up by a paediatric gastroenterologist. Preliminary results already indicate that most of these children will develop CD later in life.

For the above mentioned reasons PREVENTCD complies with article 17 of the Council of Europe Convention on Human Rights and Biomedicine Article 17 – Protection of persons not able to consent to research.

Addendum

During the start-up period of Family Intervention Study, due to delay in obtaining the gluten preparation for the intervention on time, after informed consent of their parents-guardians, the recruited children received placebo in a non-randomised, open way.

References

- Akobeng AK, Ramanan AV, Buchan I, Heller RF. Effect of breast feeding on risk of coeliac disease: a systematic review and meta-analysis of observational studies. Arch Dis Child 2006; 91: 39-43.
- Ascher H, Krantz I, Rydberg L, P Nordin, B Kristiansson. Influence of infant feeding and gluten intake on coeliac disease. *Arch Dis Child* 1997;76:113–17
- Auricchio S, Follo D, de Ritis G, Giunta A, Marzorati D, Prampolini L, Ansaldi N, Levi P, Dall'Olio D, Bossi A *et al.* Does breast feeding protect against the development of clinical symptoms of celiac disease in children? *J Pediatr Gastroenterol Nutr* 1983;2:428–33.
- Carlsson A, Agardh D, Borulf S, Grodzinsky E, Axelsson I, Ivarsson SA. Prevalence of celiac disease: before and after a national change in feeding recommendations. Scand J Gastroenterol 2006;41:553-8.
- Cataldo F, Maltese I, Paternostro D, Traverso G, Albeggiani A. Celiac disease and dietary habits in the 1st year of life. Minerva Pediatr 1991;43:7–10.
- Catassi C, Fasano A. Current approaches to diagnosis and treatment of celiac disease: an evolving spectrum.Gastroenterology 2001;120:636-51.
- Catassi C, Rossini M, Ratsch IM, Bearzi I, Santinelli A, Castagnani R, Pisani E, Coppa GV, Giorgi PL. Dose dependent effects of protracted ingestion of small amounts of gliadin in coeliac disease children: a clinical and jejunal morphometric study. Gut. 1993 Nov;34(11):1515-9.
- Common Food Intolerances 1: Epidemiology of Coeliac Disease. Auricchio S, Visakorpi JK (eds). Dyn Nutr Res. Basel, Kager 1992.
- ESPGAN Committee on Nutrition. Guidelines on infant nutrition III. Recommendations for Infant feeding. Acta Paediatr Scand Suppl 1982;302:16-20.
- Falth-Magnusson K, Franzen L, Jansson G, Laurin P, Stenhammar L. Infant feeding history shows distinct differences between Swedish celiac and reference children. Pediatr Allergy Immunol 1996;7:1–5.
- Greco L, Auricchio S, Mayer M, Grimaldi M. Case control study on nutritional risk factors in celiac disease. *J Pediatr Gastroenterol Nutr* 1988;7:395-9.
- Hopman EG, Kiefte- de Jong JC, le Cessie S, Witteman JC, Moll HA, Bleeker SE, Mearin ML. An instrument for assessment of infant gluten consumption. *Clinical Nutrtion 2007; 26: 264-71.*
- Ivarsson A, Persson LÅ, Nyström L, Ascher H, Cavell B, Danielsson L, Dannaeus A, Lindberg T, Lindquist B, Stenhammar L, Hernell O. Epidemic of coeliac disease in Swedish children. *Acta Paediatr* 2000;89:165-71.
- Ivarsson A, Hernell O, Stenlund H, Åke Persson L**.** Breast-feeding protects against celiac disease. Am J Clin Nutr 2002;75:914–21.
- Ivarsson A. The Swedish epidemic of coeliac disease explored using an epidemiological approach – some lessons to be learnt. *In: Mulder C, Cellier C. Coeliac disease. Best practice & research - Clinical Gastroenterology*, 2005. pp. 425-440.
- Norris JM, Barriga K, Hoffenberg EJ, Taki I, Miao D, Haas JE, Emery LM, Sokol RJ, Erlich HA, Eisenbarth GS, Rewers M. Risk of celiac disease autoimmunity and timing

of gluten introduction in the diet of infants at increased risk of disease. JAMA 2005; 293: 2343-51.
- Peters U, Schneeweiss S, Trautwein EA, Erbersdobler HF. A case-control study of the effect of infant feeding on celiac disease. Ann Nutr Metab 2001;45:135–42.
- Steens RFR, Csizmadia CGDS, George EK, Ninaber MK, Hira Sing RA, Mearin ML. Better recognition of childhood celiac disease in the Netherlands and its changing clinical picture: a national prospective study 1993-2000. J Pediatr 2005; 147: 239-43.
- WHO Expert Consultation. Expert consultation on the optimal duration of exclusive breast-feeding. Conclusions and recommendations. Geneva, 28-30 March 2001. http://www.who.int/
- Ziegler A, Schmid S, Huber D, Hummel M, Bonifacio E. Early infant feeding and risk of developing type 1 diabetes-associated autoantibodies. JAMA 2003; 290: 1721-8.

9.4. Handling of human biological samples and personal data

9.4.a. Human biological samples

In the family study biological material will be obtained from both the baby's and their first degree family members (parents and siblings):

- Baby's: cord blood, whole venous blood and small bowel biopsies (table 1).
- Mothers: Breast milk (table 1) and 1 whole venous blood sample (10 ml)
- Fathers: 1 whole venous blood sample (10 ml)
- Siblings: 1 whole venous blood sample (5 ml)

In the population based study only the children are involved and the sampling will be limited to whole venous blood, saliva and a small intestinal biopsy. The samples will be used to analyse biological characteristics relevant to the project, such as presence and quantity of gluten peptides, immunological mechanisms triggered by exposure to gluten (e.g. appearance of gluten peptides-specific lymphocytes in the blood or in small bowel mucosa), and to identify differences in distribution of the genetic risk factors for CD. The amount of material obtained will be limited to the minimum required for these investigations. The risk caused by the procedure will be kept to a minimum.

Collected biological material will either be used immediately for *in vitro* tests, or will be kept frozen until used in further tests. In order to allow later scientific analyses with newer techniques that are not yet available, frozen samples may be kept stored for 10 years after completion of the project, after which unused material will be destroyed. The biological samples will only be used in the scientific experiments described in PREVENTCD. No commercial use of this material will be made. The samples

pertaining to a clinical trial will be owned by the coordinating investigator responsible for that trial. Biological material will only be collected and stored after specific informed consent by the parents, guardians or other legal representatives of the children has been obtained. The informed consent information relating to the collection and storage of biological material will include a description of the procedures, amounts and risks related to the collection of samples. It will mention that these samples will only be used for the genetic and immunological tests associated with the present research project, and that no commercial use will be made thereof. This informed consent will be an integral part of the informed consent document that the parents, guardians or other legal representatives of each child will sign before inclusion in the corresponding trial.

9.4.b. Protection of personal data

Each centre participating in PREVENTCD will be responsible for protecting the confidentiality of the children and the families that it enrols. This protection will comply with the local, national and European standards for protection of privacy and confidentiality. In all cases, this will include the anonymisation of each child and his/her first degree family members (parents and siblings), by replacing his/her name with a code that will make any direct identification impossible. This code will be used in all exchange of information related to this subject. Sensitive hard copy records such as medical and trial-related records will be kept in a key locked, access-restricted place and computer access to sensitive electronic data will be password protected. Access to this information and to the identity of the study participants will be strictly restricted to the physicians in charge of the patient, data managers in charge of the study, study monitors and authorized regulatory bodies. The name or any other identifying information of participating children will not be used in publications resulting from the study.

PREVENTCD is being performed by experienced groups of clinical European paediatric gastroenterologists and researchers who already have a proven track record in performing clinical trials to the highest standards. Their ability to comply with applicable ethical rules and to undertake such studies across national boundaries has been demonstrated by the successful participation of these groups in many international multicenter trials, including clinical studies on CD. These experienced groups will continue to support and

promote the adoption of the Good Clinical Practice (GCP) standards amongst groups that are less familiar with the implications and practical application of these regulations. Part of the role of PREVENTCD in expanding the clinical network of centres undertaking clinical trials in Europe will be the explicit promotion and support of GCP-level clinical trials.
PREVENTCD will study the effect of the currently known genetic risk factors on CD and the early dietary intervention, in two cohorts: a Swedish population-based cohort and a European-based family cohort. Each individual will be genotyped for the four genes known to be associated to CD: HLA-DQ, CTLA4, CELIAC2 and XX. DNA will be isolated from peripheral blood from all participants according to standard protocols. Genotyping will be performed using 384 single nucleotide polymorphisms (SNP) covering these four regions. A specific ICF for genetic studies will be obtained, with due explanations about DNA and what the genetic study involves.

9.5. Conflicts of interest

Danone Research BV (Partner 8 of PREVENTCD) is the corporate research facility of Danone, producer of baby food and of clinical and disease specific nutrition products. Phadia GmbH (Partner 10 of PREVENTCD) is a world leader in the development and production of diagnostics kits in autoimmunity disorders, including CD. Eurospital SpA (Partner 11 of PREVENTCD) is a SME specialised in *in-vitro* diagnostic systems for the diagnosis of CD and the detection of allergies and food intolerance. The other participating investigators of PREVENT CD have not any links that might reflect a conflict of interest with the design, performance, analysis or publication of this research.

9.6. Ethical management

The partners of PREVENTCD are a group of senior health care professionals with large experience and proven competence in the management of ethical research issues. Because of the potential ethical aspects of the PREVENTCD project, the management group includes an ethical advisor, Moshe Berant, who will give advise, requested and unrequested, to the management team with respect to ethical questions, issues and decisions (figure 1).

Curriculum Vitae of the ethical advisor

Moshe Berant, MD

Born in The Hague, Netherlands, in April 11th, 1934. Emigrated to Chile in 1939 and settled in Israel in 1952.

Married (Nina Berant, MD, MPH) in 1956. Father of 5 children.

1959 – Degree of Doctor in Medicine, Hebrew University-Hadassah Medical School, Jerusalem.

1960 – 1965 Training in Pediatrics, Hadassah Univ. Hosp., Jerusalem.

1965 – 1972 Senior Pediatrician, Government Hospital, Hadera

1972 – 1979 Director of Pediatrics, Government Hospital, Hadera

1979 – 1995 Director and Head of Pediatrics, Rambam Med Ctr/Technion Faculty of Medicine, Haifa

1995 – 1999 Chairman, Division of Pediatrics, Rambam Med Ctr/Technion-Faculty of Medicine, Haifa (until retirement, May 1999)

1997 – 1999 Physician-in-Charge, Unit of Pediatric Gastroenterology and Nutrition (id, id)

Diplomas: Specialist in Pediatrics (1965)

Specialist in Pediatric Gastroenterology and Nutrition (1990)

Academic Appointments:

1984 Professor of Pediatrics (Technion-Faculty of Medicine, Haifa)

1997 Professor of Pediatric Gastroenterology and Nutrition (id.)

1992-1994 Associate Dean, Technion-Faculty of Medicine

1994-1999 Chairman of Pediatrics, Technion-Faculty of Medicine

1999 Professor (Emeritus), Technion-Faculty of Medicine

Since 1995 – Chairman of the Helsinki Committee at Rambam Medical Center

Member of the Steering Committee of the Israeli Ministry of Health for Regulatory Issues and Monitoring of Clinical Trials in Human Subjects.

In addition, PREVENTCD will create an Ethical Sub-Committee (ESC), chaired by the ethical advisor, to carry the ethical management of the research project. The ethical management will cover all the ethical aspects of the project, including research on

children and on human material, including DNA, and protection of personal data. It will remain active during the entire period of the research, and will work in close collaboration with the advisory board.

The tasks of the ethical management will be to:

- Ensure that all research activities within PREVENT CD are carried out in full respect with the ethical and safety rules described above.
- Ensure that each partner has the required knowledge on the ethical and safety issues of the project, and that this knowledge is constantly updated.
- Create an independent Data Safety Monitoring Board. This Board will ensure the safety of the patients included in PREVENTCD, by analysing adverse events and by performing interim analyses of the clinical outcome data. The leader of the ESC, Prof. M. Berant professor of paediatrics (retired), Rambam Medical Center and Technion-Faculty of Medicine, Haifa, Israel.

The members of the ESC will be independent from the research teams, chosen among the members of the ethics committee/institutional review board (EC/IRB) of each centre participating in the project. Each EC/IRB will be asked to propose one of its members, or select another faculty member to join the ESC. However, two or more EC/IRB from the same country may designate one single representative member. Thus, the ESC will be composed of at least one representative from each participating country. This will ensure a good coordination between the ESC and local EC/IRB, and will promote ethical cooperation and exchanges between EU countries.

10. Other issues (optional)

10.1 Gender dimension

The proposed project agrees with the European policy to integrate the gender dimension in FP6 projects, because:

1. The topic to be studied
2. The participants in the project

The topic of the project.

The proposed project involves coeliac disease (CD), a disorder that, as most others with autoimmune character, affects women at least twice as men. Also many of the long-term

complications of CD, such as retarded menarche age, infertility, premature births, early menopause and osteoporosis are disorders concerning directly health women's needs. Identification of early nutrition strategies for primary prevention of CD, which is the aim of the proposed project, may thus contribute to improve the health and quality of life of the European women.

The cohorts of PREVENTCD, i.e. the newborns from high-risk families (WP3) and the population based Swedish birth cohorts with twelve year of follow-up (WP4) should preferably reflect the general population with respect to gender distribution, i.e. about the same numbers of girls and boys. However, the gender difference in CD occurrence with two times as many CD cases among girls compared to boys should be taken into account for in the statistical analyses, by always considering stratifying the analyses and the reporting on results by gender. In addition, the project concerns the role of early dietary history in the development of CD, including the role of breast-feeding, a subject that concerns directly the females as mothers, as it does the subject of early nutrition in general.

The composition of the participants in the project encourages women's participation in research, since:

- The leaders of five of the seven work packages (WP) of the project, including the coordinator, are females.
- A substantial number of the participants in the proposed project are women, since they are usually dedicated to some of the key disciplines, such as Paediatrics, Clinical Nutrition, Dietetics, Genetics, Epidemiology and Public Health, necessary to carry out the project.

10.2 List of some members of the CDEUSSA European platform of experts on CD (for complete list see www.cdeusa.com)

**Name**	**Organisation**	**City**	**Country**	**Kind of organisation**
M. Berant	Rambam Health Care Campus	Haifa	Israel	University hospital
J. Bindels	Danone Research	Wageningen	The Netherlands	
E. Bravi	Eurospital	Trieste	Italy	

N. Cerf-Bensussan	Faculté Necker-enfants Malades	Paris	France	University hospital
P. Ciclitira	King's College London	London	UK	University hospital
D. Duta	Institute of food bioresources	Bucharest	Romania	
J. Ellis	King's College London	London	UK	University hospital
C. Gianfrani	Instituto di Scienze dell'Alimentazione	Roma	Italy	University hospital
L. Gilissen	Plant Research International	Wageningen	The Netherlands	
M. Gobetti	University of Bari	Bari	Italy	University
M-L. Hammarström,	Umeå University	Umeå	Sweden	University hospital
A. Ivarsson	Umeå University	Umeå	Sweden	University hospital
B. Jarry	Institut Curie	Paris	France	
K. Kaukinen	University of Tampere	Tampere	Finland	University hospital
L. Lindholm	Umeå University	Umeå	Sweden	University hospital
G. Mazzarella	Instituto di Scienze dell'Alimentazione	Roma	Italy	University hospital
E.N.C. Mills	Institute for Food Research	Norwich	UK	
Z. Misak	Children's hospital Zagreb	Zagreb	Croatia	University hospital
A. Mowat	University of Glasgow	Glasgow	Scotland, UK	University hospital
C. Mulder	Free University Medical Center	Amsterdam	The Netherlands	University hospital
E. Mummert	Phadia	Freiburg	Germany	Industry
I. Polanco	Hospital University Infantil "La Paz"	Madrid	Spain	University hospital
C. Ribes Koninckx	Hospital Infantil Universitario "La Fe"	Valencia	Spain	University hospital
M. Rossi	Instituto di Scienze dell'Alimentazione	Roma	Italy	University
C. Scerri	OAECS		Malta	Patient Association
R. Shamir	Meyer Children's Hospital of Haifa	Haifa	Israel	University hospital
L. Sollid	University of Oslo	Oslo	Norway	University hospital
R. Štěpánková	Institute of	Prague	Czech	University

	Microbiology		Republic	hospital
M..Stern	Universitäts-Kinderklinik	Tübingen	Germany	University hospital
C. O'Sullivan	Universitat Rovira i Virgili	Tarragona	Spain	
H. Tlaskalová-Hogenová	Institute of Microbiology	Prague	Czech Republic	University hospital
L. Tučková	Institute of Microbiology	Prague	Czech Republic	University hospital
J. West	University of Nottingham	Nottingham	UK	University hospital
H. Wieser	Deutsche Forschungsanstalt für Lebensmittelchemie	Garching	Germany	

10.23 List of abbreviations

ACECOVA	Valencia Coeliac Association (Asociación de Celíacos de la Comunidad Valenciana)
ACM	Madrid Coeliac Association (Asociación de Celíacos de Madrid)
AGA	IgA anti-gliadin antibodies
AOECS	Association of European Coeliac Disease Societies
CAI	Celiac Association of Israel
CDC	Dutch Coeliac Disease Consortium
CHZ	Children's Hospital Zagreb
ESPGHAN	European Society of Paediatric Gastroenterology, Hepatology and Nutrition
EC	European Commission
ELISA	Antibody based detection system
FFQ	Food frequency questionnaires
IPR	Intellectual property rights
IFN	Interferon
HP	Heim Pal Children's Hospital, Budapest, Hungary
HULF	La Fe University Hospital
HULP	La Paz University Hospital
URV	Universitat Rovira i Virgili, Hospital Universitari de Sant Joan de Reus, Tarragona, Spain
LMU	Dr. v. Haunersches Kinderspital, Ludwig Maximilians University, Munich, Germany
LUMC	Leiden University Medical Center
NCV	Dutch Coeliac Society (Nederlandse Coeliakie Vereniging)
Danone	Danone Research BV
PCS	Polish Coeliac Society
RuG	Groningen University
SNP	Single nucleotide polymorphisms
TECHNION	Technion – Israel Institute of Technology (faculty of medicine)
TTGA	IgA antibodies against tissue trans- glutaminase
UMCG	University Medical Center Groningen

UMU	Umeå University
UNINA	University of Naples, Federico II
UO	University of Oslo
VUmc	Free University Medical Center Amsterdam
WMU	Akademia Medyczna w Warszawie
WP	Work Package

11. Appendix A - Consortium description

A.1 Participants and consortium

In order to design and carry out the scientifically most robust studies and achieve the best results, internationally renowned researchers, representing the leaders of their different scientific areas of excellence are needed. The PREVENTCD group consists of leading scientists with a vast and complementary experience in the field of clinical and experimental research, clinical epidemiology, paediatric gastroenterology and nutrition, genetics, immunology, coeliac disease (CD) in collaboration with CD patient associations and industry. The PREVENTCD consortium partners include some of the best experts within key areas of CD research in Europe. They have all published extensively in prestigious journals and have performed frontline research in their respective areas. Many of the partners have leading and advisory roles in national and international committees such as the Committee on Nutrition of the European Society of Paediatric Gastroenterology, Hepatology and Nutrition (ESPGHAN) (*R. Shamir, H. Szajewska*), the Committee of Gastroenterology of ESPGHAN *(S. Koletzko)* and the ESPGHAN Council (*R. Shamir*). Furthermore, many members and advisors of the consortium have extensive experience with EU projects as coordinators, line leaders or leaders of work packages (WP), such as the Biomed European Multicentre study on CD and non-Hodgkin lymphoma BMH4-CT98-3091 (DG 12-SSMI) (*L. Mearin, H. Szajewska*), the EU-Cluster on CD (*R. Troncone, L Greco*), and the Marie Curie RTN-CT-2004-512385 "Drugs for Therapy" (Coordinator *F. Koning*).

The consortium presented in Table 1 consists of 13 research centres, 3 industrial partners and the Association of European CD Societies (AOECS). One of the participants is based outside the EU, in Israel.

Complementarity

The participants complement to each other concerning both the expertises necessary to carry out the different WP's (Child Health, Paediatric Gastroenterology, Immunology, Genetics, Nutrition) and the representation of different European social settings (Research Institutions, Industry, Patients Associations). In addition, the collaboration of the different Institutions and Patient Organisations is necessary to achieve the needed

number of families with high risk for CD expecting a newborn during the time of the project. The collaboration of the Swedish partner is necessary to compare the effect of the early nutrition history in the general population to the one in the selected population of families with increased risk, in the development of CD.

InCo Countries

There is no participation of the so called International Collaboration (InCo) countries.

SME

One of the partners involved, namely partner 11, Eurospital, is SME dedicated, among others, to the design and marketing of rapid, affordable and patient oriented diagnostic tests of CD

Table 3. Short description of the consortium stating who the participants are, what their roles and functions in the consortium are, and how they complement each other.

No.	Participant organisation's name	Country	Chief Scientist	Role in project	Area of excellence
1.	Academisch Ziekenhuis Leiden *acting under the name* Leids Universitair Medisch Centrum	Netherlands	Dr. Maria Luisa Mearin, Dr. Frits Koning	Coordinator, Partner WP3 Leader WP 6	Immunology of CD; HLA; Paediatric Gastroenterology; Assessment gluten consumption; Assessment quality of life CD patients; Co-ordination SSA on CD FP6 Food – CT- 2005-517787 CDEUSSA; Leader and member executive board (CSO) Dutch CD Consortium;

					Co-ordinator MC-RTN-CT-2004-512385 "Drugs for Therapy".
2.	University of Naples	Italy	Prof. Riccardo Troncone, Prof. Luigi Greco	Leader WP 3<br>Partner WP5<br>Partner WP6	Immunology;<br>Genetics of CD;<br>Paediatric Gastroenterology;<br>Epidemiology of CD;<br>Partner SSA Food – CT- 2005-517787 CDEUSSA
3.	Umeå University	Sweden	Dr. Anneli Ivarsson, Prof. Dan Holmberg	Leader WP 4<br><br>Partner WP 5	WHO collaborating centre on epidemiological surveillance;<br>Epidemiology of CD;<br>Child Public Health.<br>Infant nutrition;<br>Genetics of complex diseases;<br>Partner SSA Food – CT- 2005-517787 CDEUSSA
4.	Akademia Medyczna Warszawie	Poland	Prof. Hania Szajewska	Leader WP 7<br>Partner WP3	Nutrition;<br>Paediatric Gastroenterology;<br>Clinical research<br>Partner SSA Food – CT- 2005-517787 CDEUSSA;<br>Partner No ERB IC20-CT98-0203 (BMH4-CT98-3091) Proposal No PL 97 3091
5.	University	Netherland	Prof. Cisca	Leader WP 5	Human Genetics;

	Medical Centre Groningen		Wijmenga		Biomedical Genetics; Genetics of CD; Member executive board (CSE) Dutch CD Consortium
6.	La Paz University Hospital, Madrid	Spain	Prof. Isabel Polanco	Partner WP 3	Paediatric Gastroenterology; Care of children and families with CD; Genetics of CD; Nutrition
7.	La Fe University Hospital, Valencia	Spain	Prof. Carmen Ribes-Koninckx	Partner WP 3	Paediatric Gastroenterology; Gluten detection in food products; Immunology of CD; Genetics, care of children and families with CD
13.	University of Oslo	Norway	Prof. Ludvig Sollid	Partner WP6	Immunology; HLA; Immunology of CD
14.	Children's Hospital Zagreb	Croatia	Prof. Sanja Kolacek	Partner WP3	Nutrition; Paediatric Gastroenterology; Care of children and families with CD
15.	Heim Pal Children's Hospital	Hungary	Prof. Ilma Korponay-Szabo	Partner WP3	Immunology, Transglutaminase research, Paediatric Gastroenterology; Care of children and families with CD
16.	Universitat Rovira i Virgilli	Spain	Dr. Gemma Castillejo De Villasante Prof. J Escribano	Partner WP3	Paediatric Gastroenterology; Care of children and families with CD

17.	Dr. v. Haunersches Kinderspital Ludwig Maximilians University	Germany	Prof. Sibylle Koletzko	Partner WP3	Paediatric Gastroenterology; Care of children and families with CD
**Patients' organization**					
9.	Association of European Coeliac Disease Societies	Belgium	Dr. Christian A. Scerri	Partner WP7	Coordination international activities, Exchange of information among the member Societies
**Industry**					
8.	Danone Research BV	Netherlands	Prof. Jacques Bindels	Partner WP3	Nutrition, Food Technology, Clinical trials
10.	Phadia GmbH	Germany	Dr. Eckart Mummert, Dr. Nicola Cronin	Partner WP3 Partner WP4	Design, development production and marketing of diagnostic systems for testing autoimmune diseases
11.	Eurospital SpA	Italy	Dr. Enzo Bravi	Partner WP3 Partner WP4	Design, development production and marketing of diagnostic systems for Gastroenterology and Paediatrics
**Participants based outside of the EU and Associated states**					
12.	TECHNION – Israel Institute of Technology (faculty of medicine)	Israel	Prof. Raanan Shamir	Partner WP 3	Nutrition; Paediatric Gastroenterology; Care of children and families with

					CD

The partners

Leiden University Medical Centre (LUMC) (Partner 1) ranks among the top immunology institutes worldwide. The Department of Immunohaematology and Blood Transfusion (Dr. F Koning) is well known for its research on HLA and HLA-associated diseases, including CD. And the collaboration with the Department of Paediatric Gastroenterology (Dr. ML Mearin) has contributed significantly to the recent progress in this field.

Dr. Maria Luisa Mearin (MD, PhD) will contribute to the project with her more than 20 years of experience in the diagnosis and treatment of children with CD. She will bring in her experience, coming from her involvement in a number of international collaborations with scientists both from within Europe and from other countries. Moreover, she has gained experience in the coordination of the EU funded project on CD and cancer, which has performed in 10 European countries (Biomed 2 Programme 1996; BMH4-CT98-3091; DG 12-SSMI) and in the coordination of the EU funded project CDEUSSA (Food – CT- 2005- 517787), which aim it is to close the gap between basic science on CD and the applications of its results in prevention, clinical treatment and the food industry.

Relevant publications; a selection

1. Hopman GD, Romijn JA, le Cessie S, von Blomberg BME, **Mearin ML**. Nutritional management of the gluten-free diet in young people with celiac disease in the Netherlands. Accepted J Pediatr Gastroenterol Nutr.
2. Monsuur AJ¸ Stepniak D, Diosdado B, Wapenaar , **Mearin ML,** Koning F, Wijmenga C. Genetic and functional analysis of pyroglutamyl-peptidase I in celiac disease. Accepted. E J Gastroenterol Hepatol.
3. **Mearin ML,** Catassi C, Brousse N, Brand R, Collin P, Fabiani E, Schweizer JJ, Abuzakouk M, Szajewska H, Hallert C, Farré Masip C, Holmes GKT on behalf of the Biomed Study Group on Coeliac Disease and non-Hodgkin lymphoma. European multicenter study on coeliac disease and Non-Hodgkin lymphoma. E J Gastroenterol Hepatol 2006;18:187-4.
4. Steens RFR, Csizmadia CGDS, George EK, Ninaber MK, Hira Sing RA, **Mearin ML.** Better recognition of childhood celiac disease in the Netherlands and its changing clinical picture: a national prospective study 1993-2000. J Pediatr 2005; 147: 239-243.
5. **Mearin ML,** Ivarsson A, Dickey W. Coeliac disease: is it time for mass screening? Best Pract Research Clin Gastroenterol 2005;19 :441-52
6. Monsuur AJ, de Bakker PIW, Alizadeh BZ, Bevova MR Franke L, van t' Slot R,

van Belzen MJ, Diosdado B, Zhernakova A, Daly MJ, Mulder CJJ, **Mearin ML,** Meijer JWR, Meijer GA, van Oort E, Wapenaar M, Koeleman BPC, Wijmenga C. Myosin IXB variant increases the risk of celiac disease and points towards a primary intestinal barrier defect. Nature Genetics 2005; 37:1341-4

7. Schweizer JJ, von Blomberg ME, Bueno-de Mesquita HB, **Mearin ML**. Coeliac disease in the Netherlands. Scand J Gastroenterol 2004;39:359-64.
8. Vader W, Stepniak DT, Kooy Y, **Mearin ML,** Thompson A, Spaenij EHA, Koning F. The HLA-DQ2 gene dose effect in celiac disease is directly related to the magnitude and breadth of gluten-specific T cell responses. Proc Natl Acad Sci USA 2003; 14:12390-5.
9. Vader W, Kooy Y, Van Veelen P, De Ru A, Harris D, Benckhuijsen W, Peña S, **Mearin L**, Drijfhout JW and Koning F. The gluten response in children with celiac disease is directed toward multiple gliadin and glutenin peptides. Gastroenterology 2002;122:1729–37.
10. Kolsteren MMP, Koopman HM, Schalekamp G, **Mearin ML**. Health-related quality of life of children with celiac disease. J Pediat 2001; 138:593-5.

Dr. Frits Koning (PhD) is professor of Immunology in the Department of Immunohaematology and Blood Transfusion (IHB) of the Leiden University Medical Centre. Dr. Koning will bring his more than 20 years of experience in immunology and biochemistry. He is section leader in the Department of IHB and head of the mass spectrometry unit. Dr. Koning is an internationally recognized expert in the field of CD as evidenced by invitations to speak during many international meetings on CD. He is the Chief Executive Officer of the Dutch-based CDC and the coordinator of the MC-RTN-CT-512385 "Drugs for Therapy". He participates in national and international (EU) projects on CD. His group was the first to identify a gluten peptide that was recognised by gluten-specific T cells isolated from the small intestine of a CD patient. More recently he has elucidated the specificity of tissue transglutaminase, an enzyme that is involved in gluten toxicity, work that has contributed significantly to our understanding of the molecular basis for the association between HLA-DQ2/8 and CD. He has developed an antibody-based test to screen food and foodstuffs for the presence of toxic gluten peptides. He has filed four patent applications, including three in the field of CD.

Relevant publications; a selection

1. Wal van de Y, Kooy Y, Veelen van P, Pena S, Mearin L, Papadopulos G, and **Koning F.** Cutting Edge: Selective deamidation by tissue transglutaminase strongly enhances gliadin-specific T cell reactivity. J. Immunol. 161, 1585-1588 (1998)

2. Wal van de Y, Kooy Y, Veelen van P, Pena S, Mearin L, Molberg Q, Lundin L, Mutis T, Benckhuijsen W, Drijfhout J.W, and **Koning F.** Small intestinal cells of celiac disease patients recognize a natural pepsin fragment of gliadin. Proc. Natl. Acad. Sci. USA. 95, 10050-10054 (1998)
3. Wal van de, Y., Kooy, Y.M.C., Veelen, van P., August, S.A., Drijfhout, J.W. and **Koning, F.** Glutenin is involved in the gluten-driven mucosal T cell response. Eur. J. Immunol. 29, 3133-3139 (1999).
4. Vader, W., Kooy, Y., van Veelen, P., de Ru, A., Harris, D., Benckhuijsen, W., Pena, S., Mearin, L., Drijfhout, J.W., **Koning, F.** The gluten response in children with recent onset celiac disease. A highly diverse response towards multiple gliadin and glutenin derived peptides. Gastroenterology, 122: 1729-1737 (2002).
5. Vader W, de Ru A, van der Wal Y, Kooy Y, Benckhuijsen W, Mearin L, Drijfhout JW, van Veelen P, and **Koning F.** Specificity of tissue transglutaminase explains cereal toxicity in celiac disease. J. Exp. Med. 195: 643-649 (2002).
6. W. Vader, D. Stepniak, E. M. Bunnik, Y. Kooy, W. de Haan, J.W. Drijfhout, P. A. van Veelen, **F. Koning** Characterization of cereal toxicity for celiac disease patients based on protein homology in grains. Gastroenterology, 125: 1105-13 (2003)
7. W. Vader, D. Stepniak, Y. Kooy, M. L. Mearin, A. Thompson, L.Spaenij, and **F. Koning.** The HLA-DQ2 gene dose effect in Celiac Disease is directly related to the magnitude and breadth of gluten-specific T-cell responses. Proc. Natl. Acad. Sci. USA. 100: 12390-5 (2003)
8. D Stepniak, LW Vader, Y Kooy, PA van Veelen, A Moustakas, NA Papandreou, E Eliopoulos, JW Drijfhout, GK Papadopoulos, and **F Koning**. T cell recognition of HLA-DQ2-bound gluten peptides can be influenced by an N-terminal proline at p-1. Immunogenetics, 57: 8-15 (2005)
9. L Spaenij-Dekking, Y Kooy-Winkelaar, P van Veelen, JW Drijfhout, H Jonker, L van Soest, MJM Smulders, D Bosch, LJWJ Gilissen, and **F Koning**. Natural variation in toxicity of wheat accessions for celiac disease patients. Potential for selection and breeding of non-toxic wheat varieties. Gastroenterology, 129: 797-806 (2005)
10. Spaenij-Dekking EHA, Kooy Y, **Koning F.** The Ethiopian Cereal Tef in Celiac Disease. NEJM, 353: 1748-1749 (2005)

The University Federico II of Naples (Partner 2)

The University Frederico II of Naples currently follows more than 1000 coeliac patients. The same Centre coordinates all clinical activities related to CD in the Regione Campania of Italy.

Prof. Riccardo Troncone (MD, PhD) will contribute to the project with his knowledge of Paediatric Gastroenterology and of mucosal immunology, with special reference to CD.

Relevant publications; a selection

1. Gianfrani C, **Troncone R**, Mugione P, Cosentini E, De Pascale M, Faruolo C, Senger S, Terrazzano G, Southwood S, Auricchio S, Sette A. Celiac disease

association with CD8+ T cell responses: identification of a novel gliadin-derived HLA-A2 restricted epitope. J Immunol 2003; 170: 2719-26.

2. Mazzarella G, Maglio M, Paparo F, Nardone G, Stefanile R, Greco L, van de wal Y, Kooy Y, Koning F, Auricchio S, **Troncone R**. An immunodominant DQ8-restricted gliadin peptide activates small intestinal immune response in in vitro cultured mucosa from HLA-DQ8, but not HLA-DQ2 positive coeliac patients. Gut 2003; 52: 57-62.
3. **Troncone R**, Franzese R, Mazzarella G, Paparo F, Auricchio R, Coto I, Mayer M, Greco L. Gluten sensitivity in a subset of children with insulin-dependent diabetes mellitus. Am J Gastroenterol 2003; 98: 590-595.
4. Mazzarella G, MacDonald T T, Salvati V M, Mulligan P, Pasquale L, Stefanile R, Lionetti P, Auricchio S, Pallone F, **Troncone R.** Constitutive activation of the signal transducer and activator of transcription pathway in coeliac disease lesions. Am. J Pathol. 2003; 162: 1845-1855.
5. Salvati V M, MacDonald T T, Del Vecchio Blanco G, Mazzarella G, Monteleone I, Vavassori P, Auricchio S, Pallone F, **Troncone R.** Enhanced expression of interferon regulatory factor-1 in the mucosa children with coeliac disease. Pediatr Res. 2003; 54: 312-8.
6. Esposito C, Paparo F, Caputo I, Porta R, Salvati VM, Mazzarella G, Auricchio S, **Troncone R**. Expression and enzymatic activity of small intestinal tissue transglutaminase in celiac disease. Am J Gastroenterol 2003; 98: 1813-20.
7. Auricchio R, Paparo F, Maglio M, Franzese A, Lombardi F, Valerio G, Nardone G, Percopo S, Greco L, **Troncone R.** In vitro deranged intestinal immune response to gliadin in type 1 diabetes. Diabetes 2004: 53: 1680-3.
8. Salvati VM, Mazzarella G, Gianfrani C, Levings M, Stefanile R, De Giulio B, Iaquinto G, Giardullo N, Auricchio S, Roncarolo M, **Troncone R**. Recombinant human IL-10 suppresses gliadin-dependent T-cell activation in ex vivo cultured celiac intestinal mucosa. Gut 2005; 54: 46-53.
9. Paparo F, Petrone E, Tosco A, Maglio M, Borrelli M, Salvati V.M, Miele E, Greco L, Auricchio S, **Troncone R**. Clinical, HLA, and small bowel immunohistochemical features of children with positive serum antiendomysium antibodies and architecturally normal small intestinal mucosa. Am J Gastroenterol 2005; 100: 2294-8.
10. Senger S, Maurano F, Mazzeo M.F, Gaita M, Fierro O, David C.S, **Troncone R**, Auricchio S, Siciliano R.A, Rossi M. Identification of immunodominant epitopes of {alpha}- gliadin in HLA-DQ8 transgenic mice following oral immunization. J Immunol 2005; 175: 8087-8095.

Prof. Luigi Greco (MD, PhD) has a leading role in coordinating the research on genetics of CD disease in Europe. Both will bring in their experience and the contacts gained in the coordination of the “EU cluster on celiac disease”, funded by the EU in the context of the 5th framework.

Relevant publications; a selection

1. Lorenza Nisticò, Corrado Fagnani, Iolanda Coto, Selvaggia Percopo, Rodolfo Cotichini, Maria Giovanna Limongelli, Franco Paparo, Sandra D'Alfonso, Mara Giordano, Concettina Sferlazzas, Giuseppe Magazzù, Patricia Momigliano-Richiardi, **Luigi Greco**, Maria Antonietta Stazi. Concordance, disease progression, and heritability of coeliac disease in Italian twins. Gut. 2005 Dec 14; [Epub ahead of print]
2. Paparo F, Petrone E, Tosco A, Maglio M, Borrelli M, Salvati VM, Miele E, **Greco L**, Auricchio S, Troncone R.
   Clinical, HLA, and small bowel immunohistochemical features of children with positive serum antiendomysium antibodies and architecturally normal small intestinal mucosa.
   Am J Gastroenterol. 2005 Oct;100(10):2294-8.
3. Margaritte-Jeannin P, Babron MC, Bourgey M, Louka AS, Clot F, Percopo S, Coto I, Hugot JP, Ascher H, Sollid LM, **Greco L**, Clerget-Darpoux F.
   HLA-DQ relative risks for coeliac disease in European populations: a study of the European Genetics Cluster on Coeliac Disease.
   Tissue Antigens. 2004 Jun;63(6):562-7.
4. Holopainen P, Naluai AT, Moodie S, Percopo S, Coto I, Clot F, Ascher H, Sollid L, Ciclitira P, **Greco L**, Clerget-Darpoux F, Partanen J; Members of the European Genetics Cluster on Coeliac Disease.
   Candidate gene region 2q33 in European families with coeliac disease.
   Tissue Antigens. 2004 Mar;63(3):212-22.
5. Mazzarella G, Maglio M, Paparo F, Nardone G, Stefanile R, **Greco L**, van de Wal Y, Kooy Y, Koning F, Auricchio S, Troncone R.
   An immunodominant DQ8 restricted gliadin peptide activates small intestinal immune response in in vitro cultured mucosa from HLA-DQ8 positive but not HLA-DQ8 negative coeliac patients. Gut. 2003 Jan;52(1):57-62.
6. Louka AS, Moodie SJ, Karell K, Bolognesi E, Ascher H, **Greco L**, Momigliano-Richiardi P, Partanen J, Ciclitira PJ, Sollid LM.
   A collaborative European search for non-DQA1*05-DQB1*02 celiac disease loci on HLA-DR3 haplotypes: analysis of transmission from homozygous parents.
   Hum Immunol. 2003 Mar;64(3):350-8.
7. Troncone R, Franzese A, Mazzarella G, Paparo F, Auricchio R, Coto I, Mayer M, **Greco L**.
   Gluten sensitivity in a subset of children with insulin dependent diabetes mellitus.
   Am J Gastroenterol. 2003 Mar;98(3):590-5.
8. Karell K, Louka AS, Moodie SJ, Ascher H, Clot F, **Greco L**, Ciclitira PJ, Sollid LM, Partanen J.
   Hla types in celiac disease patients not carrying the DQA1*05-DQB1*02 (DQ2) heterodimer: results from the european genetics cluster on celiac disease.
   Hum Immunol. 2003 Apr;64(4):469-77.
9. Maiuri MC, De Stefano D, Mele G, Fecarotta S, **Greco L**, Troncone R, Carnuccio R. Nuclear factor kappa B is activated in small intestinal mucosa of celiac patients. J Mol Med. 2003 Jun;81(6):373-9. Epub 2003 May 13.
10. Percopo S, Babron MC, Whalen M, De Virgiliis S, Coto I, Clerget-Darpoux F,

Landolfo F, **Greco L**. Saturation of the 5q31-q33 candidate region for coeliac disease. Ann Hum Genet. 2003 May;67(Pt 3):265-8.

Umeå University (UMU), Sweden, (Partner 3) The Umeå University with its unit for *Epidemiology and Public Health Sciences* within the Department of Public Health and Clinical Medicine, Faculty of Medicine, is nationally and internationally acclaimed for its high quality research within Epidemiology and Public Health Sciences. The unit was recently designated as a World Health Organisation (WHO) collaborating centre with a responsibility to develop and implement epidemiological surveillance, specifically of non-communicable disease, and to promote surveillance enhancing the effectiveness of disease prevention. The unit of *Medical and Clinical Genetics* integrates basic research and service laboratories, and is affiliated with the clinical genetics laboratory at the University Hospital of Northern Sweden. As a part of the Umeå Centre for Molecular Medicine (UCMM) it is localized in an environment of diverse biomedical laboratories, which forms a creative and interactive unit for cutting edge biomedical research (supported by the EU regional funding).

Dr. Anneli Ivarsson (MD, PhD) is a specialist in Paediatrics, a Senior lecturer in epidemiology, and is also attached to the Research and Development Unit of the Västerbotten County Council. She will contribute her experience resulting from her integral and varying roles in multicentre population based epidemiological studies, her vast knowledge in public health sciences, and her general knowledge as a specialist in Paediatrics with a longstanding clinical and research interest in CD. Being responsible for the Swedish National incidence register of CD in children, and attached multicentre studies, she is well acquainted with handling large multicentre research databases. Exploring the unique Swedish epidemic of CD in a genetically stable population, and attributing part of the epidemic to changed infant feeding practices, has awakened the international research community to explore options for primary prevention of this lifelong and common disease. Her research findings have already led to revised national Swedish infant feeding recommendations.

Relevant publications; a selection:

1. **Ivarsson A**, Persson LÅ, Nyström L, Ascher H, Cavell B, Danielsson L, Dannaeus A, Lindberg T, Lindquist B, Stenhammar L, Hernell O. Epidemic of coeliac disease in Swedish children. Acta Paediatr 2000;**89**:165-71.
2. Largerqvist C, **Ivarsson A**, Juto P, Persson LÅ, Hernell O. Screening of adult celiac disease - which serological marker(s) to use? J Intern Med 2001;**250**:241-8.
3. **Ivarsson A**, Hernell O, Stenlund H, Persson LÅ. Breast-feeding protects against coeliac disease. Am J Clin Nutr 2002;**75**:914-21
4. **Ivarsson A**, Hernell O, Nyström L, Persson LÅ. Children born in the summer have an increased risk for coeliac disease. J Epidemiol Community Health 2003;**57**:36-39.
5. **Ivarsson A**, Persson LÅ, Nyström L, Hernell O. The Swedish coeliac disease epidemic with a prevailing two-fold higher risk in girls compared to boys may reflect gender specific risk factors. Eur J Epidemiol 2003;18:677-684.
6. Hernell O, Forsberg G, Hammarström S, **Ivarsson A**, Hammarström M-L. Coeliac disease: a model to study oral tolerance. In: Hart AL, Stagg AJ, Graffner H, Glise H, Falk P, Kamm MA. Gut Ecology. London: Martin Dunitz, 2002. pp.73-79.
7. **Ivarsson A**, Persson LÅ, Hernell O. Primary prevention of coeliac disease by favourable infant feeding practices. In Catassi C, Fasano A, Corazza GR, eds. Primary prevention of celiac disease – the utopia of the next millennium? Pisa: AIC Press, 2003. pp 43-60
8. Collin P, Kaukinen K, Vogelsang H, Korponay-Szabò I, Sommer R, Schreier E, Volta U, Granito A, Veronesi L, Mascart F, Ocmant A, **Ivarsson A**, et al. Antiendomysial and antihuman recombinant tissue transglutaminase antibodies in the diagnosis of coeliac disease: a biopsy-proven European multicenter study. Eur J Gastroenterol Hepatol 2005;**17**:85-91
9. Mearin LM, **Ivarsson A**, Dickey W. Celiac disease: Is it time for mass screening? In: Mulder C, Cellier C. Coeliac disease. Best practice & research - Clinical Gastroenterology, 2005. pp.441-452.
10. **Ivarsson A**. The Swedish epidemic of coeliac disease explored using an epidemiological approach – some lessons to be learnt. In: Mulder C, Cellier C. Coeliac disease. Best practice & research - Clinical Gastroenterology, 2005. pp. 425-440.

Prof. Dan Holmberg (PhD), professor in Genetics, will contribute experience from his extensive research on diseases with a complex aetiology, interfacing genetics and lifestyle factors. Responsible for the Division of Medical and Clinical Genetics and the Genotyping core facility associated with this unit, he will also contribute the infrastructure for high throughput genotyping and statistical analyses. He leads a group of about 10 people who focus on understanding the genetics of complex diseases with an emphasis on studies in the comparatively homogeneous Northern Swedish population.

Relevant publications; a selection

1. Colucci, F., Bergman, M.-L., Penha-Gonçalves, C., Cilio, C. and **Holmberg, D. A** locus (Nod5) controlling apoptosis resistance in lymphocytes of diabetic mice maps to the Idd5 diabetes susceptibility region. Proc. Natl. Acad. Sci. USA 94:8670, 1997
2. Cilio, C.M., M.R. Daws, A. Maleshnikova, C.L. Sentman and **D. Holmberg** CTLA-4 is induced in the thymus upon in vivo activation and its blockade prevents anti-CD3 mediated depletion of thymocytes. J. Exp. Med. 188:1239-1246, 1998
3. Bagot, S., Campino, S., Penha-Goncalves, C., Pied, S., Cazenave, P-A. and **Holmberg, D**. Identification of two novel cerebral malaria resistance loci using an inbred wild derived mouse strain Proc Natl Acad.Sci USA 99:9919-9923, 2002
4. Einarsdottir E, Soderstrom I, Lofgren-Burstrom A, Haraldsson S, Nilsson-Ardnor S, Penha-Goncalves C, Lind L, Holmgren G, Holmberg M, Asplund K, **Holmberg D**. The CTLA4 region as a general autoimmunity factor: An extended pedigree provides evidence for synergy with the HLA locus in the etiology of type 1 diabetes mellitus, Hashimoto's thyroiditis and Graves' disease. Eur J Hum Genet. 11:81-84, 2003
5. Bergman, M-L., Duarte, N.,Campino, S.,Lundholm, M., Lejon, K., Penha Gonçalves, C. and **Holmberg, D**. Diabetes protection and restoration of apoptosis resistance in NOD Idd6 congenic strains Diabetes 52: 1677-1682, 2003
6. Einarsdottir E, Carlsson A, Minde J, Toolanen G, Svensson O, Solders G, Holmgren, G, **Holmberg D**, Holmberg M. A mutation in the nerve growth factor beta gene (NGFB) causes loss of pain perception. Hum Mol Genet.13:799-805, 2004
7. Rolf, J., Motta, V., Duarte, N., Lundholm, M., Berntman, E., Bergman, M-L., Sorokin, L., Cardell, S. and **Holmberg, D**. The enlarged population of marginal zone/CD1d^{high} B lymphocytes in non-obese diabetic mice appears at the time of disease initiation and maps to diabetes susceptibility region Idd11 J. Immunol. 174:4821-4827, 2005
8. Nilsson-Ardnor, S., Wiklund, P.-G., Lindgren, P., Nilsson, A. K., Janunger, T., Escher, S., Hallbeck, B., Stegmayr, B., Asplund, K. and **Holmberg, D**. Replication of the Icelandic linkage to the PDE4D region on 5q and suggested alternative genotypes associated with ischemic stroke in a Swedish population Stroke. 36:1666-1671, 2005
9. Lundholm, M., Motta, V., Löfgren-Burström, A., Duarte, N., Bergman, M-L., Eklund, S. and **Holmberg, D.** The NOD allele of IL-2 interacts with CTLA-4 and a novel locus telomeric on chromosome 1 in conferring deficient CTLA expression and lymphocyte apoptosis resistance in NOD mice. Diabetes. 55:538-44, 2006
10. Einarsdottir, E., Mayans, S., Ruikka, K., Escher S. A. , Lindgren, P., Ågren, Å., Eliasson M. and **Holmberg, D**. Linkage but not Association of Calpain-10 to Type 2 Diabetes Replicated in Northern Sweden. Diabetes (in press)

The Medical University of Warsaw (Partner 4) is one of Poland's top institutions in medical education and research. The Department of Dermatology is internationally acknowledged for contribution to the discovery of anti-endomysial antibodies used in the screening of CD. The Department of Paediatric Gastroenterology and Nutrition is the country's leading centre for diagnosis and treatment of CD patients in Poland.

Prof. Hania Szajewska (MD, PhD) is a professor of Paediatrics at this institute. As she is currently holding the position of Secretary of the Committee on Nutrition of the ESPGHAN, she will also bring in her contacts with the Committee. This Committee is actively involved in generating European guidelines for early nutrition. Since the actual guidelines for early nutrition may play a detrimental role in CD development, it is therefore a great importance that this Committee is involved in the proposed project.

Relevant publications; a selection

1. **Szajewska H**, Horvath A, Koletzko B, Kalisz M.
   Effects of brief exposure to water, breast-milk substitutes, or other liquids on the success and duration of breastfeeding: a systematic review.
   Acta Paediatr. 2006 Feb;95(2):145-52.
2. Mearin ML, Catassi C, Brousse N, Brand R, Collin P, Fabiani E, Schweizer JJ, Abuzakouk M, **Szajewska H**, Hallert C, Farre Masip C, Holmes GK; Biomed Study Group on Coeliac Disease and Non-Hodgkin Lymphoma.
   European multi-centre study on coeliac disease and non-Hodgkin lymphoma.
   Eur J Gastroenterol Hepatol. 2006 Feb;18(2):187-94.
3. **Szajewska H**, Dziechciarz P, Mrukowicz J.
   Meta-analysis: Smectite in the treatment of acute infectious diarrhoea in children.Aliment Pharmacol Ther. 2006 Jan 15;23(2):217-27.
4. Decsi T, Fidler Mis N, Kolacek S, Kon I, Kopecky J, Penas-Jimenez I, Socha P, **Szajewska H**. Challenges and opportunities in Pan-European collaboration for researchers from Central and Eastern Europe. Adv Exp Med Biol. 2005;569:54-9. Review.
5. **Szajewska H**, Mrukowicz J. Meta-analysis: non-pathogenic yeast Saccharomyces boulardii in the prevention of antibiotic-associated diarrhoea. Aliment Pharmacol Ther. 2005 Sep 1;22(5):365-72. Review.
6. Agostoni C, Axelson I, Colomb V, Goulet O, Koletzko B, Michaelsen KF, Puntis JW, Rigo J, Shamir R, **Szajewska H**, Turck D; ESPGHAN Committee on Nutrition; European Society for Paediatric Gastroenterology. The need for nutrition support teams in pediatric units: a commentary by the ESPGHAN committee on nutrition. J Pediatr Gastroenterol Nutr. 2005 Jul;41(1):8-11. Erratum in: J Pediatr Gastroenterol Nutr. 2005 Aug;41(2):267-71.
7. Horvath A, Koletzko B, Kalisz M, **Szajewska H**. The effect of supplemental fluids or feedings during the first days of life on the success and duration of

breastfeeding: a systematic review of randomized controlled trials. Arch Pediatr Adolesc Med. 2005 Jun;159(6):597-8. Review. No abstract available.
8. **Szajewska H**, Mrukowicz JZ. Use of probiotics in children with acute diarrhea. Paediatr Drugs. 2005;7(2):111-22. Review.
9. Banaszkiewicz A, **Szajewska H**. Ineffectiveness of Lactobacillus GG as an adjunct to lactulose for the treatment of constipation in children: a double-blind, placebo-controlled randomized trial. J Pediatr. 2005 Mar;146(3):364-9.
10. Kotowska M, Albrecht P, **Szajewska H**. Saccharomyces boulardii in the prevention of antibiotic-associated diarrhoea in children: a randomized double-blind placebo-controlled trial. Aliment Pharmacol Ther. 2005 Mar 1;21(5):583-90.

The University Medical Centre Groningen (UMCG) (Partner 5) has a leading position in many medical disciplines, education and research and is closely related to Groningen University (RuG). The RuG integrates biomedical research and education at the faculties of chemistry, biology and pharmaceutical sciences. The UMCG and RuG together create a unique combination of biomedical sciences to study fundamental biological issues as well as clinical issues in both humans and animals.

Prof. Cisca Wijmenga (PhD) will bring knowledge on the genetics of CD and the expertise and infrastructure required to perform large-scale genetic studies. In addition, her lab will perform a large part of the studies and will be involved in the data analysis. Cisca Wijmenga is a full professor in Human Genetics, and head of the Genetics Department of the UMCG. She heads a moderate size research group (of 1 PI, 4 postdocs and 12 PhD students) which focuses on the genetics of complex diseases, using genetic linkage/association studies and genomics technology (microarrays) and which has contributed significantly to the genetics of CD. She was able to identify new genetic loci on 6q, 9p and 19p. More recently, she identified the Myo9B gene (on 19p13.1), the first non-HLA CD gene identified through positional cloning. Recently she completed a genome-wide association study which led to the identification of a novel CD susceptibility gene. She also runs an Illumina facility for genetica (SNP) and gene expression studies. Moreover, she developed appropriate databases for genetic studies (GIDS) and new software tools (TEAM, SHOPS, PRIORITIZER) to aid in data-mining and constructing gene networks. Cisca Wijmenga has much national and international collaboration, with both clinical and basic researchers. She is chief scientific officer (human genomics) of the Dutch Coeliac Disease Consortium (CDC).

Relevant publications; a selection

1. Diosdado B, van Bakel H, Strengman E, Franke L, van Oort E, Mulder CJJ, **Wijmenga C,** Wapenaar MC (2007) A genomics view on celiac disease points to enhanced neutrophil recruimtment and barrier impairment. Clin Gastroenterol Hepat (in press).
2. Monsuur AJ, **Wijmenga C** (2006) Understanding the molecular basis of celiac disease: what genetic studies reveal. Ann Med 38(8):578-591.
3. van Bodegraven AA, Curley CR, Hunt KA, Monsuur AJ, Linskens RK, Onnie

CM, Crusius BA, Annese V, Latiano A, Silverberg MS, Bitton A, Fisher SA, Steinhart AH, Forbes A, Sanderson J, Prescott NJ, Strachan DP, Playford RJ, Mathew CG, Daly MJ*, Rioux JD*, van Heel DA*, **Wijmenga C***(2006) Genetic variation in myosin IXB is associated with ulcerative colitis. Gastroenterology 131(6):1768-1774. (*shared last authors)

4. de Bakker PIW, McVean G, Sabeti PC, Miretti MM, Green T, Marchini J, Ke X, Monsuur AJ, Whittaker P, Delgado M, Morrison J, Richardson A, Walsh EC, Gao X, Galver L, Hart J, Hafler DA, Pericak-Vance M, Todd JA, Daly MJ, Trowsdale J, **Wijmenga C, Vyse** TJ, Beck S, Shaw Murray S, Carrington M, Gregory S, Deloukas P, Rioux JD (2006) A high resolution HLA and SNP haplotype map for disease association studies in the extended human MHC. Nat Genet 38(10):1166-72.
5. Franke F, van Bakel H, Fokkens L, de Jong E, Petersen M-E, **Wijmenga C** (2006) Reconstruction of a functional human gene network, with an application for prioritizing positional candidate genes. Am J Hum Genet 78(6):1011-25.
6. Hunt KA, Monsuur A, Kumar PJ, Travis SPL, Walters JRF, Jewell DP, Playford RJ, **Wijmenga C**, van Heel DA (2006) Association studies of MYO9B genetic variants in a British coeliac disease cohort. Gut 55(7):969-972.
7. Monsuur AJ, de Bakker PIW, Alizadeh BZ, Zhernakova A, Bevova MR, Strengman E, Franke L, van het Slot R, van Belzen MJ, Lavrijsen ICM, Diosdado B, Daly MJ, Mulder CJJ, Mearin ML, Meijer JWR, Meijer GA, van Oort E, Wapenaar MC, Koeleman BPC, **Wijmenga C** (2005) Myosin IXB variant increases the risk of celiac disease and points towards a primary intestinal barrier defect. Nature Genet 37: 1341-1344
8. Zhernakova A, Eerlig P, Barrera P, Weseloy JZ, Huizinga TWJ, Roep BO, **Wijmenga C**, Koeleman BPC (2005) CTLA4 is differentially associated with autoimmune diseases in the Dutch population. Hum Genet 118: 58-66.
9. Koning F, Gilissen L, **Wijmenga C** (2005) Gluten: a two-edged sword. The molecular basis of Celiac Disease. In: Springer seminars in immunopathology. Springer Semin Immunopathol 27:217-232.

La Paz University Hospital. (Partner 6) The Department of Paediatric Gastroenterology and Nutrition of La Paz Hospital is a well recognised centre for CD nationally and internationally.

Prof. Isabel Polanco (MD, PhD) is a Full Professor of Paediatrics at the Autonoma University of Madrid (Spain) and head of the Department of Paediatric Gastroenterology and Nutrition of the Hospital Universitario La Paz of Madrid. She will contribute to the project with her knowledgeof Paediatric Gastroenterology and Nutrition and of the recruitment of families with at least one member with CD from the Comunidad Autónoma de Madrid. CD represents her main interest of research and most of her

scientific production is focused on this area, especially on genetics and environmental factors. She is a member of ESPGHAN (General Secretary from 1990 – 1993) and presently she is General Secretary of the Spanish Paediatric Association. She has a big experience in CD. During the last 30 years, she has diagnosed and treated more than 1000 coeliac patients. She is a scientific advisor of the Federacion de Asociaciones de Celiacos de España (F.A.C.E) and represents a link with patients' associations. She has conducted and collaborated in several national and international studies on CD.

Relevant publications; a selection

1. Ghisolfi J, Roberfroid M, Rigo J, Moro G, **Polanco I**. Infant formula supplemented with probiotics or prebiotics: never, now, or someday? J Pediatr Gastroentrol Nutrition 2002; 4: 467-468.
2. Beltri P, Prieto G, Molina M, Sarria J, **Polanco I**. Are dental enamel defects a characteristic of patients with coeliac disease indeed? J Pediatr Gastroentrol Nutrition 2002; 4: 470-471.\
3. **Polanco I**, Martinez Bermejo A. The prevalence of headache in a population of patients with Coeliac Disease. Rev Neurol 2004; 32: 301 – 309.
4. Polanco I. Clinical relevance of villous atrophy. Pediatr Allergy Inmunol. 2003; 12: 47 – 50.
5. **Polanco, I**. Celiac Disease. En: Lifschitz CH, ed. Pediatric gastroenterology and nutrition in clinical practice. Marcel Dekker, Inc. New York: 2002: 517-535.
6. Polanco I. Diagnostic criteria – zero or how many biopsies. In: Makki M, Collin P, Visakorpi JK, eds. Coeliac Disease. Tampere: Celiac Disease Study Group, 1997: 171 – 176.
7. Spurkland A, Sollid LM, **Polanco I**, Vartdal F, Thorsby E. The CD associated HLA-DQαß heterodimer may be encoded by unusual haplotypes. Human Immunology 1993;5:
   162-7.
8. **Polanco I**, Mearin ML, Larrauri J, Biemond I, Wipkink-Bakker A, Peña AS. The effect of gluten suplementation in healthy siblings of children with celiac disease. Gastroenterology 1987; 92: 678 – 681.
9. **Polanco I**, Fernández M, Gómez de la Concha E. "Interleukin-10 haplotypes in Celiac Disease in the Spanish population". Am J Gastroenterology 2005. In press: AJG – 06 – 0088.
10. López-Santamaría M, Gámez M, Murcia M, …, Molina M, **Polanco I**, Jara P, Tovar J. Pediatric intestinal transplantation. Transplant Proc 2003; 35(5): 1927-8.
    Comas A, Bixquert M, Polanco I. Constipation characteristics in childhood and adult life. Gut 2004; 54: 21–25.

La Fe University Hospital (Partner 7) is one of Spain's top institutions in medical education and research, and is the reference hospital of the Comunidad Valenciana (≈ 4.750.000 inhabitants).

Prof. Carmen Ribes (MD, PhD) will bring her more than 20 years in Paediatric Gastroenterology, with special reference to diagnosis and treatment of CD and clinical research in this field. She leads Paediatric Gastroenterology Unit of La Fe Children's Hospital, and coordinating all clinical and investigation activities on CD in the Comunidad Valenciana. The latest project, concerns the characterization of the genetics of CD in the Comunidad Valenciana, through family studies. She is also involved in studies measuring gluten contents in foods and will contribute to establish in the gluten intake by infants enrolled in the study. Carmen Ribes will also bring her experience gained from the coordination of clinical studies on CD, both at the national level and at the Comunidad Valenciana, and from international studies on CD. She is main investigator and co investigator in several projects on CD supported by the Instituto de Salud Carlos III and FIS.

Relevant publications; a selection

1. J.A. Garrote, L Sorell, P. Alfonso, B. Acevedo, L. Ortigosa, **C. Ribes**, J. Gavilondo, E. Mendez. A novel visual immunoassay for coeliac disease screening".European Jorunal of Clinical Investigation . 1999 Vol, 20 : 697-699.
2. **C. Ribes-Koninckx**, et al A (-turn rich oats peptide as an antigen in an ELISA method for the screening of coeliac disease in a paediatric population",. European Journal of Clinical Investigation 2000; Vol 30: 702-708.
3. **C. Ribes-Koninckx**, et al Dynamics of antitissue transglutaminase antibodies (tTG)in coeliac children after gluten withdrawal and during gluten challenge.GUT 2001; Vol 49 (Supp. III): A2128
4. E. Llorente, E. Reyes, ML Hernaez, E. Garcia, E. Donat, B. Polo, **C. Ribes-Koninckx**, L. Ortigosa, E. Mendezal Are rice and maize based foods safe for coeliac patients? . GUT 2001; Vol 49 (Supp. III): A2303
5. **C Ribes-Koninckx**, S Ferre-López, , C Genzor, S Gamen, L Peña, L Ortigosa, E Méndez. A reliable generation of immunochromatographic sticks for both t-TG and AGA screening in coeliac disease. Clinical Gastroenterology & Hepatology 2004; 2:
6. Picarelli A, Sabbatella L, AnaniaMC, Calabro A, **Ribes-Koninckx C**, Donat Aliaga E, Gasparini M, Bravi E; Multicentre Organ Culture System Study Group.Usefulness of the organ cukture system in the in vitro diagnosis of coeliac disease: a multicentre study. Scand. J. Gastroenterology (2006); 41 (2): 186-190

7. Donat-Aliaga, M. Planelles-Silvestre, A. Capilla-Villanueva, J. Montoro-Alberola, **C. Ribes-Koninckx**. HLA class II genotype in celiac disease patients and first degree relatives: Impact on clinical disease expression (2006. SUBMITTED)
8. Capilla A, Donat E, Espinos C, Palau F, **Ribes- Koninckx C**. Genotipic study of non HLA regions associated with coeliac disease. (2006. SUBMITTED).
9. L. Blesa, Donat-Aliaga, **C. Ribes-Koninckx**. Coeliac Disease diagnosis: Is it time for rapid visual serological testing? (2006, Submitted)

Danone Research BV (Partner 8) is the corporate research facility of Danone with expertise in baby food, clinical and disease specific nutrition and food technology.

Prof. J.G. Bindels *(PhD)* will contribute by channelling the expertise and facilities of Danone Research BV into the project. As a nutritionist with a biochemical background, and as a former professor of Nutrition during growth and development (Wageningen University, The Netherlands), combined with his more than 15 years of experience in designing and executing nutrition based clinical studies, his participation will be of significant value to the project. He will specifically contribute by designing and manufacturing the intervention products (versus placebo) for WP3 and arrange for the blinding and randomisation process in WP3. He will be the independent guardian of the randomisation code, using appropriate techniques and procedures to ensure maximal blindness of study participants and investigators to the assigned study products. Yet, he will have a system in place to quickly reveal the identity of the study product / group assignment in cases of adverse events. Danone will provide the placebo (lactose, Pharmatose) and the intervention product (Glutival obtained from Cargill). Danone has outsourced the production of the sachets with the gluten intervention / placebo products to NIZO Food Research, Ede, the Netherlands. NIZO will prepare and package the gluten intervention product as well as the placebo according to quality criteria adequate for infants aged 4-6 months.. Every intervention sachet will be filled 1.8 gr Pharmatose and 0.2 gr Glutival (gluten intervention product); the placebo sachets will be filled with 2 gr of Pharmatose.

NIZO will pay special attention to the microbiological assessment of the products and will make sure that the results will be in accordance with the standards of Danone. Also it

will be analytically verified whether the blending of the materials has been adequate and resulted into a homogeneous mixture.

Relevant publications; a selection

1. Kennedy K, Fewtrell MS, Moreley R, Abbott R, Quinlan PT, Wells JCK, **Bindels JG**, Lucas A (1999) Double-blind, randomized trial of a synthetic triacylglycerol in formula-fed term infants: effects on stool biochemistry, stool characteristics, and bone mineralisation. AJCN 70: 920-927.
2. Harmsen HJM, Wildeboer-Veloo ACM, Raangs GC, Wagendorp AA, Klijn N, **Bindels JG**, Welling GW (2000). Analysis of intestinal flora development in breast-fed and formula-fed infants using molecular identification and detection methods. JPGN 30:61-67.
3. Wouters-Wesseling W, Rozendaal M, Snijder M, Graus Y, Rimmelzwaan G, de Groot L, **Bindels JG** (2002) Effect of a complete nutritional supplement on antibody response to influenza vaccine in elderly people. J Gerontol Med Sci.57: 563-566
4. Beulens JWJ, **Bindels JG**, de Graaf C, Alles MS, Wouters-Wesseling W (2004). Alpha-lactalbumin combined with a regular diet increases plasma Trp-LNAA ratio. Physiol Behav 81: 585-593.
5. Kieft H, Roos AN, van Drunen JDE, Bindels AJGH, **Bindels JG**, Hofman Z (2005). Clinical outcome of immunonutrition in a heterogeneous intensive care population. Intensive Care Med 31: 524-532
6. Bakker-Zierikzee A, Alles M, Knol J, Kok F, Tolboom J, **Bindels J** (2005) Effects of infant formula containing a mixture of galacto- and fructo oligosaccharides or viable Bifidobacterium animalis on the intestinal flora during the first 4 months of life. Br J Nutr, 94: 783-790.
7. Verwimp JJ, **Bindels JG**, Barents M, Heymans HS. Symptomatology and growth in infants with cow's milk protein intolerance using two different whey-protein hydrolysate based formulas in a Primary Health Care setting.Eur J Clin Nutr. 1995 Sep;49 Suppl 1:S39-48.
8. Wouters-Wesseling W, Wagenaar LW, Rozendaal M, Deijen JB, de Groot LC, **Bindels JG**, van Staveren WA. Effect of an enriched drink on cognitive function in frail elderly persons. J Gerontol A Biol Sci Med Sci. 2005 Feb;60(2):265-70.
9. Koletzko B, Aggett PJ, **Bindels JG**, Bung P, Ferre P, Gil A, Lentze MJ, Roberfroid M, Strobel S. Growth, development and differentiation: a functional food science approach. Br J Nutr. 1998 Aug;80 Suppl 1:S5-45. Review.
10. Jacobs NJ, van Zoeren-Grobben D, Drejer GF, **Bindels JG**, Berger HM. Influence of long chain unsaturated fatty acids in formula feeds on lipid peroxidation and antioxidants in preterm infants. Pediatr Res. 1996 Nov;40(5):680-6.

<u>The Association of European Coeliac Societies (AOECS) (Partner 9)</u> is the umbrella organisation of European Coeliac Societies and is an independent non-profit association.

Its membership base consists of 30 regional or national societies across 25 European countries, representing over 200,000 coeliac families. It is thus ideally situated to recruit coeliac subjects from its membership base, as well as to desseminate the results and recommendations.

Dr Christian A. Scerri, (PhD) is a member of the board of directors of AOECS and presently its chair. He has a vast experience in subject recruitment for epidemiological and genetic studies, having been responsible for the Maltese WP in the Eurobiobank project as well as in the recently terminated Geoparkinson project.

Phadia GmbH (Partner 10), is a world leader in the development and production of diagnostics kits in autoimmunity. The Phadia Biotechnikum II in Freiburg was established as a Centre of Excellence for autoimmune test research and production. All processes are performed using GMP guidelines and are ISO approved. Using human recombinant tissue transglutaminase (tTG), produced in a eukaryotic cell system, Phadia has established the Celikey range of products for the diagnosis and monitoring of CD. The Celikey products provide exceptional sensitivity and specificity in this area and their performance is well recognised in many publications. Phadia will contribute to the study by supplying Celikey test kits for detection of IgA and IgG anti tTG antibodies as well as IgA and IgG anti gliadin kits. The company will also provide their expertise, manpower and the facilities of their Application laboratory to perform all the testing.

Relevant publications; a selection

1. **Dahlbom I**, Olsson M, Forooz NK, Sjöholm AG, Truedsson L, **Hansson T** (2005)
   Immunoglobulin G (IgG) anti-tissue transglutaminase antibodies used as markers for IgA-deficient celiac disease patients
   Clin Diagn Lab Immunol 12 (2), 254-258
2. Collin P, Kaukinen K, Vogelsang H, Korponay-Szabó I, Sommer R, Schreier E, Volta U, Granito A, Veronesi L, Mascart F, Ocmant A, Ivarsson A, Lagerqvist C, Bürgin-Wolff A, Hadziselimovic F, Furlano RI, Sidler MA, Mulder CJJ, Goerres MS, Mearin ML, Ninaber MK, Gudmand-Høyer E, Fabiani E, Catassi C, **Tidlund H**, Alainentalo L, Mäki M (2005)
   Antiendomysial and antihuman recombinant tissue transglutaminase antibodies in the diagnosis of coeliac disease: a biopsy-proven European multicentre study
   Eur J Gastroenterol Hepatol, 17: 85–91

3. Luyckx A, Westhovens R, Oris E, **Papisch W**, Bossuyt X (2005)
Clinical relevance of Measurement of Antibodies to Individual snU1-RNP Proteins
Clin Chem 51, 1888-1890
4. Midhagen G, Åberg A-K, Olcén P, Järnerot G, Valdimarsson T, **Dahlbom I, Hansson T**, Ström M (2004)
Antibody levels in adult patients with coeliac disease during gluten-free diet: a rapid initial decrease of clinical importance
J Int Med 256, 519–524
5. Villalta D, Tonutti E, Tampoia M, Bizzaro N, **Papisch W**, Tozzoli R, Stella S (2004)
Analytical and diagnostic accuracy of the EliA™ automated enzyme fluoroimmunoassay for antineutrophil cytoplasmic autoantibody detection
Clin Chem Lab Med 42 (10):1161–1167

<u>Eurospital SpA (Partner 11)</u>

Eurospital represents as SME consolidated and innovative company specialised in in-vitro diagnostic systems, prescription and over-the-counter products, clinical-surgical instruments, and medical equipment for the hospital market. The diagnostics division R&D laboratory develops innovative diagnostic systems in the field of Gastroenterology and immunology. Eurospital develops, industrializes, produces and markets diagnostic kits and automated instrumentation and software, including a complete range of ELISA and IFI kits for the diagnosis of CD and the detection of allergies and food intolerance.

Relevant publications; a selection

1. Picarelli A, Di Tola M, Sabbatella L, Anania MC, Calabro A, Renzi D, Bai JC, Sugai E, Carroccio A, Di Prima L, Bardella MT, Barisani D, Ribes-Koninckx C, Aliaga ED, Gasparin M, **Bravi E**; Multicentre Organ Culture System Study Group.Usefulness of the organ culture system in the in vitro diagnosis of coeliac disease: a multicentre study. Scand J Gastroenterol. 2006 Feb;41(2):186-90.
2. Fabiani E, Peruzzi E, Mandolesi A, Garbuglia G, Fanciulli G, D'Appello AR, Gasparin M, **Bravi E**, Bearzi I, Galeazzi R, Catassi C. Anti-human versus anti-guinea pig tissue transglutaminase antibodies as the first-level serological screening test for coeliac disease in the general population.
Dig Liver Dis. 2004 Oct;36(10):671-6.
3. S. A. McMillan and **E. Bravi** Anti-tissue transglutaminase antibodies in IgA deficient sera might a proper cut-off value be of help in avoiding total serum IgA testing? Clinical and Applied Immunology Reviews Volume 3, Issues 1-2 , November 2002, Page 13
4. Sandro Drago, **Enzo Bravi**, Mariarosaria Di Pierro, Giuseppe Iacono, Michelle Pietzak, Karoly Horvath and Alessio Fasano Comparative study between American and Italian families of Celiac disease patients based on HLA profiles

Clinical and Applied Immunology Reviews, Volume 3, Issues 1-2, November 2002, Page 12

5. M. A. Pelli, M. Maldini, C. Contini, E. Tonutti, A. Carroccio, M. Cottone, A Montanelli, R. Gusmaroli, S. Contenti, S. Catani and **E. Bravi**
   Use of Calprest® in the diagnosis of Inflammatory Bowel Disease (IBD) Clinical and Applied Immunology Reviews, Volume 3, Issues 1-2, November 2002, Pages 13-14
6. G. Tolazzi, M. Gasparin, F. Rapagna, A. Savoini and **E. Bravi**
   Eu-tTG®IgG umana: internal validation of a powerful diagnostic assay for the identification of IgA deficient celiac patients
   Clinical and Applied Immunology Reviews, Volume 3, Issues 1-2, November 2002, Pages 15-16

Technion – Israel Institute of Technology (Partner 12) In operation since 1924, the Technion is the oldest university in Israel. The university offers degrees in science and engineering, and related fields such as architecture, medicine, industrial management and education in an intellectually invigorating environment. At the institute, scientific instruction is interwoven with professional ethics, producing leaders sensitive to social and environmental issues. The Division of Paediatric Gastroenterology and Nutrition of the Meyer Children's Hospital of Haifa, associated to the Institute, serves as the National Centre for HLA typing for CD patients.

Prof. Raanan Shamir (MD, PhD, associated professor Technion – Israel Institute of Technology) will bring his experience in conducting large studies aimed at identifying subjects with CD, such as the blood donor study to identify the prevalence of CD in Israel. Through his commitment to the CD community in Israel, serving as a medical advisor to the Israel Coeliac Association, the Coeliac Society in Israel is already committed to this study and Raanan Shamir will enrol families on a National basis. Raanan Shamir will also contribute to preparing and conducting nutrition surveys and will assist in the development of preventive education and treatment strategies for CD. As a member of the ESPGHAN Committee on Nutrition (CoN), and as the CoN representative at the ESPGHAN Council, he will also take care of the dissemination of the results at different levels: i.e. the scientific community, industry, regulation bodies and patients' associations.

Relevant publications; a selection

1. **R. Shamir**, A. Levine, M. Yalon-Hacohen, R. Shapiro, I. Zahavi, Y. Rosenbach, A. Lerner, G. Dinari. Fecal occult blood in children with coeliac disease. Eur J Pediatr 2000;159:832-4
2. **R. Shamir**, I. Koren, Y. Rosenbach, R. Shapiro, I. Zahavi and G. Dinari. Celiac, fatty liver and pancreatic insufficiency. J Pediatr Gastroenterol Nutr 2001; 32:490-2
3. R. Shamir, R. Eliakim, N. Lahat, E. Sobel, A. Lerner. ELISA of anti endomysial antibodies in the diagnosis of celiac disease: comparison with immunofluorescence assay of anti endomysial antibodies and tissue transglutaminase antibodies. Isr Med Assoc J 2002;4:594-6.
4. Lerner, **R. Shamir.** Influence of environment and knowledge on celiac disease-new perspectives. Harefuah 2002;141:46-7
5. **R. Shamir,** Y. Shoenfeld, M. Blank, R. Eliakim, N. Lahat, E. Sobel, E. Shinar, A. Lerner. The prevalence of coeliac disease antibodies in patients with the antiphospholipid syndrome. Lupus 2003;12:394-399
6. **R. Shamir**. Advances in celiac disease. Gastroenterol Clin North Am 2003;32:931-47.
7. **R. Shamir,** A. Lerner, E. Shinar, N. Lahat, H. Kerner, R. Eliakim. The use of a Single Serological Marker Underestimates the Prevalence of Celiac disease in Israel: a Prevalence Study of Blood Donors. Am J Gastroenterol 2002;97:2589-94.
8. H. Shamaly, A. Mahameed, A. Sharony, **R. Shamir.** Infertility and Celiac Disease. Do we need more than one serological marker? Acta Obst Gyn Scan 2004;83:1184-8.
9. C. Hartman, B. Hino, A. Lerner, O. Eshach-Adiv, D. Berkowitz, R. Shaoul, A. Pacht, E. Rozenthal, A. Tamir, H. Shamali, **R. Shamir.** Bone Quantitative Ultrasound and Bone Mineral Density Assessment in Children with Celiac Disease. J Pediatr Gastroenterol Nutr 2004;39:504-10
10. **R. Shamir,** O. Hernell, M. Leshno. Cost-Effectiveness Analysis of Screening for Celiac Disease in the Adult Population. Med Decis Making 2006; In press

University of Oslo (Partner 13) is the largest university of Norway, and the Institute of Immunology at Rikshospitalet University Hospital is by far the country's largest immunology centre. It performs research and national service in transplantation and medical immunology. It has about 120 employees more than half of which are involved in research.

Prof. Ludvig M. Sollid (MD, PhD) is professor of medicine (immunology) at this institute, and he is the head of a research group of about 15 people. Prof. Sollid has participated in four EU projects, and he has been the co-ordinator of two EU-RTD

projects on CD (BMH4983087 and QLK1-CT-2000-00657). He and his group have made many key findings in relation to the genetics and immunology of CD. He identified DQ2 and DQ8 to be responsible for the HLA effect in CD. His group made the seminal findings that gluten reactive T cells can be cultured from intestinal biopsies of CD patients (but not controls), and that these T cells recognize gluten peptides in the context of the disease associated DQ2 or DQ8 molecules. These findings have paved the road for many subsequent findings of CD pathogenesis. His group played a central role in identifying the first sequence of a DQ2 restricted gluten T cell epitope. Importantly, Ludvig Sollid and his co-workers reported that this epitope is deamidated. They later showed that the deamidation of this and other gluten T cell epitopes is mediated by the enzyme tissue transglutaminase. His group was able to produce soluble DQ2 molecules. These molecules have been used to stain and visualize gluten specific T cells with HLA tetramers, and to solve the crystal structure of DQ2 in complex with a deamidated gluten peptide. Sollid is the member of advisory boards of several foundations, companies and research centres dealing with CD both in the US and in Europe.

Relevant publications; a selection

1. **Sollid LM**, Jabri B. Is celiac disease an autoimmune disorder? Curr Op Immunol. 17:595-600, 2005. Epub 2005 Oct 7.
2. Qiao SW, Bergseng E, Molberg Ø, Jung G, Fleckenstein B, **Sollid LM**. Refining the rules of gliadin T cell epitope binding to the disease-associated DQ2 molecule in celiac disease: importance of proline spacing and glutamine deamidation. J Immunol. 175:254-261, 2005.
3. Bergseng E, Xia J, Kim CY, Khosla C, **Sollid LM**. Main chain hydrogen bond interactions in the binding of proline-rich gluten peptides to the celiac disease associated HLA-DQ2 molecule. J Biol Chem. 280:21791-21796, 2005.
4. Molberg Ø, Uhlen AK, Jensen T, Flaete NS, Fleckenstein B, Arentz-Hansen H, Lundin KEA, **Sollid LM**. Mapping of gluten T cell epitopes in the ancestors of the modern bread wheat; implications for celiac disease. Gastroenterology. 128:393-401. 2005.
5. Arentz-Hansen H, Fleckenstein B, Molberg Ø, Scott H, Koning F, Jung G, Roepstorff P, Lundin KEA, **Sollid LM**. The molecular basis for oat intolerance in celiac disease patients. PLoS Medicine. 1: 84-92, 2004.
6. Kim CY, Quarsten H, Bergseng E, Khosla C, **Sollid LM**. Structural basis for HLA-DQ mediated presentation of gluten epitopes in celiac disease. Proc Natl Acad Sci USA. 101: 4175-4179, 2004
7. Fleckenstein B, Qiao SW, Larsen MR, Jung G, Roepstorff P, **Sollid LM**. Molecular characterization of covalent complexes between tissue transglutaminase and gliadin peptides. J Biol Chem. 279:17607-17616, 2004.

8. Shan L, Molberg Ø, Parrot I, Hausch F, Gray GM, **Sollid LM** Khosla C. Structural basis for gluten intolerance in celiac sprue. Science 297:2275-2279, 2002.
9. **Sollid LM**. Celiac disease - dissecting a complex inflammatory disorder (Review). Nat Rev Immunol 2:647-655, 2002.
10. Fleckenstein B, Molberg Ø, Qiao, SW, Schmid DG, von der Mulbe F, Elgstøen K, Jung G, **Sollid LM**. Gliadin T cell epitope selection by tissue transglutaminase in celiac disease: Role of enzyme specificity and pH influence on the transamidation versus deamidation process. J Biol Chem 277, 34109-34116, 2002.

Children's Hospital Zagreb (Partner 14) is the teaching unit for paediatric medicine of the Zagreb Medical School. It is a nationally and internationally recognized centre for education and research, prevailing in genetics, oncology, and paediatric gastroenterology & nutrition. Due to its excellence, the Department for Gastroenterology has been the State Referral Centere for Paediatric Gastroenterology & Nutrition of the Croatian Ministry of Health since 1997. Different national and international projects on the subject of CD have continuously been organized during the past 15 years, with those in the fields of epidemiology and cytogenetics being the most important.

Prof. Sanja Kolaček (MD, PhD), as a paediatric gastroenterologist and as the founder of the Croatian Coeliac Society, will bring clinical knowledge and expertise on CD. Moreover, as the co-author of several European guidelines and recommendations (i.e. Feeding in acute diarrhoea, H. pylori infection in children, Diagnosis & treatment of IBD, ESPGHAN / ESPEN evidence based guidelines on parenteral nutrition in children) she will assist in the development of European preventive and therapeutic strategies based on the results of this project.

Relevant publications; a selection

1. **Kolaček S**, Kapetanović T, Lužar V. Early determinants of cardiovascular risk factors in adults: A. Plasma lipids. Acta Paediatr 1993;82:699-82
2. **Kolaček S**, Kapetanović T, Zimolo A, Lužar V. Early determinants of cardiovascular risk factors in adults: B. Blood pressure. Acta Paediatr 1993;82:377-82
3. **Kolaček S**, Grgurić J, Percl M, et al. Home-made modular diet versus semi-elemental formula in the treatment of chronic diarrhea of infancy: A prospective randomized trial. Europ J Paediatr 1996;155:997-1001.
4. **Kolaček S**, Petković I, Booth IW. Chromosome aberrations in celiac and non-coeliac enteropathies. Arch Dis Child 1998;78:466-8

5. Grgurić J, **Kolaček s**, Lulić-Jurjević R. Multi-Indicator survey on children's nutrition in Croatia. Coll Antropol 1998;22:85-95.
6. Matek Z, Jungvirth-Hegeduš M, **Kolaček S**. Epidemiology of Coeliac disease in children in one Croatian County: Factors that could influence the incidence. Coll Antropol 2000;24:397-404.
7. Matek Z, Jungvirth-Hegeduš M, **Kolaček S**. Epidemiology of Coeliac disease in children in one Croatian County – the cumulative incidence over ten-year period. Coll Antropol 1999;23:621-8
8. **Kolaček S**. Coeliac disease: atypical presentation, complications and associated disorders. Slov Pediatr 2000;7 (Suppl 1):68-71
9. **Kolaček S**, Jadrešin O, Petković I, et al. Gluten-free diet has a beneficial effect on chromosome instability in lymphocytes of children with Coeliac disease. J Pediatr Gastroenterol Nutr 2004;38:177-180.
10. **Kolaček S**. GUT Immunity. Paediatr Croat 2005;Supl1):89-93 IBD Working Group of the European Society for Paediatric Gastroenterology and Nutrition. Medical Position Paper. Inflammatory bowel disease in children and adolescents: Recommendations for diagnosis – The Porto Criteria. J pediatr Gastroenterol Nutr 2005;41:1-7.

Heim Pal Children's Hospital (partner 15)
Heim Pal Children's Hospital is the largest child health institution in Hungary. The Coeliac Disease Centre is coordinating CD diagnostics and research in Budapest and in the Western parts of Hungary. The center itself follows 1000 CD patients and collaborates in the care of additional 2200 patients.

Dr. Ilma Korponay-Szabo (MD, PhD) will contribute to the project with her knowledge of Paediatric Gastroenterology, mucosal immunology and antibody studies. She has a research experience in designing and developing serology diagnostic tools for CD, and in the adaptation of such tools for clinical care, screening and epidemiology studies. Her interest is focused on detecting early signs of developing CD. She has contributed to several international and multi-centre studies on CD. Ilma Korponay-Szabo is associated professor in paediatrics also at the University of Debrecen, Hungary, and resposible for the pediatric gastroenterology care in the Nord-Eastern Region of Hungary. She is involved in the transglutaminase research team at Debrecen University, which is Centre of Excellence in biochemistry. Since the enzyme transglutaminase is a major player modifying gliadin and a prominent autoantigen in CD, her knowledge in basic research on transglutaminase will help to identify possible links between molecular biology, prevention and future treatment of CD.

Relevant publications; a selection

1. Sulkanen S, Halttunen T, Laurila K, Kolho K-L, **Korponay-Szabo IR**, Sarnesto A, Savilahti E, Collin P, Mäki M. Tissue transglutaminase autoantibody enzyme-linked immunosorbent assay in detecting celiac disease. Gastroenterology 1998;115:1322-8

2. **Korponay-Szabo IR**, Laurila K, Szondy Zs, Halttunen T, Szalai Zs, Rantala I, B.Kovács J, Fésüs L, Mäki M. Missing endomysial and reticulin binding of coeliac antibodies in transglutaminase 2 knockout tissues. Gut 2003;52:199-204
3. **Korponay-Szabo IR**, Dahlbom I, Laurila K, Koskinen S, Woolley N, Partanen J, Kovacs JB, Mäki M, Hansson T. Elevation of IgG antibodies against tissue transglutaminase as a diagnostic tool for coeliac disease in selective IgA deficiency. Gut 2003;52:1567-71
4. **Korponay-Szabo IR**, Halttunen T, Szalai Z, Laurila K, Király R, B.Kovács J, Fésüs M, Mäki M. In vivo targeting of intestinal and extraintestinal transglutaminase 2 by coeliac autoantibodies Gut 2004; 53:641-8
5. **Korponay-Szabo IR**, Raivio T, Laurila K, Opre J, Kiraly R, Kovacs JB, Kaukinen K, Fesus L, Maki M. Coeliac disease case finding and diet monitoring by point-of-care testing. Aliment Pharmacol Ther 2005;22:729-37
6. Kaukinen K, Peraaho M, Collin P, Partanen J, Woolley N, Kaartinen T,Nuutinen T, Halttunen T, Maki M, **Korponay-Szabo I**. Small-bowel mucosal transglutaminase 2-specific IgA deposits in coeliac disease without villous atrophy: a prospective and randomized clinical study. Scand J Gastroenterol 2005,40:564-72.
7. Salmi TT, Collin P, **Korponay-Szabo IR**, Laurila K, Partanen J, Huhtala H, Kiraly R, Lorand L, Reunala T, Maki M, Kaukinen K. Endomysial antibody-negative coeliac disease: clinical characteristics and intestinal autoantibody deposits. Gut 2006;55:1746-53
8. Király R, Vecsei Z, Deményi T, **Korponay-Szabo IR**, Fésüs L. Coeliac autoantibodies can enhance transamidating and inhibit GTPase activity of tissue transglutaminase: dependence on reaction environment and enzyme fitness. J Autoimmun 2006;26:278-87.
9. Hadjivassiliou M, Maki M, Sanders DS, Williamson CA, Grunewald RA, Woodroofe NM, **Korponay-Szabo IR**. Autoantibody targeting of brain and intestinal transglutaminase in gluten ataxia. Neurology 2006;66:373-7
10. Garcia-Horsman JA, Venäläinen J, Lohi O, Auriola OS, **Korponay-Szabo IR**, Kaukinen K, Mäki M, Männistö PT. Deficient activity of mammalian prolyl oligopeptidase on the immunoactive peptide digestion in coeliac disease. Scand J Gastroenterol. In press

<u>Universitat Rovira i Virgili- Hospital Sant Joan de Reus (Partner 16)</u>

The Hospital Sant Joan de Reus a teaching hospital associated to the University Rovira i Virgili created in 1991 by the Parliament of Catalonia to restore the Tarragona University of the 16th century. The aim of the University is to place knowledge at the service of society. The Hospital Sant Joan de Reus forms part of the Pere Virgili Institute for Health Research from the University Rovira i Virgili, founded in September 2004. The main aim of the Institute is to promote research effort, and develop and integrate basic University research with the clinical research that is being carried out in the hospitals on public health in the Tarragona region and the Terres de l'Ebre and to promote research into preventing and treating the most important health problems. The Hospital Sant Joan de Reus will leader the project in Catalonia. (7.000.000 inhabitants).

Dr. Gemma Castillejo (MD) is a young doctor with plenty experience in diagnosis and treatment of CD. She is responsible of the Paediatric Gastroenterology Unit of Hospital Sant Joan de Reus and Associated Professor of the Universitat Rovira i Virgili. She is working in her PhD about fibre and constipation in children and collaborating in the CHOP and ERNEST European projects with the same subject. Gemma Castillejo is involved in a CD study supported by the Instituto de Salud Carlos III and FIS. She is a medical advisor of the Catalonian Celiac Association (SMAP- Celíacs de Catalunya)

Relevant publications; a selection

1. **Castillejo G**, Bullo M, Anguera A, Escribano J, Salas-Salvado J. A controlled, randomized, double-blind trial to evaluate the effect of a supplement of cocoa husk that is rich in dietary fiber on colonic transit in constipated pediatric patients. Pediatrics 2006 ;118: 641-8.
2. Alonso-Villaverde C, **Castillejo G**, Gonzalez J, Olive JM. Idiopathic polyneuritis and IgA monoclonal gammapathy. Rev Clin Esp 1993; 192: 54-5.

Dr. v. Haunersches Kinderspital, Ludwig Maximilians University (partner 17)

Ludwig Maximilians University Munich is one of the three Universities which has been awarded as one centers of excellence in Germany in 2007. The Department of Gastroenterology and Hepatology at the Dr. v. Haunersche Kinderspital is the largest one in Germany for children with different gastrointestinal disorders, particularly inflammatory bowel disorders. The care of children with CD has a long tradition in the hospital.

Prof. Sibylle Koletzko (MD, PhD) is the head of the Department of Paediatric Gastroenterology and nutrition at the Dr. von Haunersche Kinderspital at the Ludwig Maximilians University of Munich. Her current research involves projects in inflammatory bowel disease, *Helicobacter pylori* infection in children, CD and food allergy. She is principal investigator of the German infant nutrition intervention (GINI) study, in which a cohort of 2252 children with a hereditary risk for allergy were enrolled at birth and is now followed for 10 years. This is the largest randomised trial conducted in children at risk for allergy to reduce the risk of allergic disease through nutritional intervention. With this experience, she will contribute to the nutritional intervention trial in the PREVENTCD project. Her first study in CD was performed in the early 80ies in >1000 children with type 1 diabetes mellitus - about 50% of them with newly diagnosed disease - to search for the prevalence CD in this population. Currently, she is secretary of GI-committee of the European society of Paediatrc Gastroenterology and Nutrition (ESPGHAN), and vice president of the German speaking society of Pediatric Gastroenterology and Nutrition (GPGE).

Relevant publications; a selection

1. **S. Koletzko**, A. Bürgin-Wolff, B. Koletzko, M. Knapp, W. Burger, D. Grüneklee, W. Ruch, A. Thon, U. Wendel, K. Zuppinger: Prevalence of celiac disease in children and adolescents with insulin dependent diabetes mellitus. Eur J Pediatr 1988; 148:113-117
2. **S. Koletzko**, M. Haisch, I. Seeboth, B. Braden, K. Hengels, B. Koletzko, P. Hering. Evaluation of a low-cost isotope selective nondispersive infrared spectrometer for detecting Helicobacter pylori infection with the ^{13}C-urea breath test. Lancet 1995; 345:961-62
3. v. Berg, **S. Koletzko**, A. Gruebl, B. Filipiak-Pttroff, H. E. Wichmann, C.P. Bauer, D. Reinhardt, D. Berdel for the GINI Study Group. The effect of cow’s milk hydrolysates for allergy prevention in the first year of life: The German Infant Nutritional Intervention Study (GINI), a randomised double-blind trial. J Allerg Clin Immunology 2003;111:533-40
4. **S. Koletzko**, N. Konstantopoulos, D. Bosman, A. Feydt-Schmidt, A. v.d. Ende, N. Kalach, J. Raymond, H. Rüssmann. Evaluation of a novel monoclonal enzyme immunoassay for detection of H. pylori antigen in stool in children. Gut 2003; 52:804-806
5. B. Laubereau, I. Brockow, A. Zirngibl, **S. Koletzko,** A. Gruebl, A. v. Berg, B. Filipiak- Pittroff, D. Berdel, CP Bauer, D. Reinhardt, J. Heinrich, H. E. Wichmann, for the GINI Study Group. Effect of breastfeeding on the development of atopic dermatitis during the first 3 years of life – results from the GINI-birth cohort study. J Pediatr 2004:144:602-7
6. S. Krauss-Etschmann, R. Gruber, H. Demmelmair, D. Reinhardt, **S. Koletzko**. Increase of antigen presenting cells in gastric mucosa of Helicobacter pylori infected children. Helicobacter 2005;10:214-22
7. B. Laubereau, B. Filipiak-Pittroff, A. v. Berg, A. Gruebl, D. Reinhardt, H. E. Wichmann, **S. Koletzko** for the GINI Study Group. Caesarean section and gastrointestinal symptoms, atopic dermatitis, and sensitization during the first year of life. Arch Dis Childh 2004;89:993-7
8. Kapple M, Krauss-Etschmann S, Diehl V, Zeilhofer H, **Koletzko S**. Detection of scretory IgA antibodies against gliadin and human tissue transglutaminase in stool has no value to screen for celiac disease in children. BMJ 2006, 332:213-4
9. **S. Koletzko**, F. Richy, P. Bontems, J. Crone, N. Kalach, M. Lurdes Monteiro, F. Gottrand, D. Celinska-Cedro, E. Roma-Giannikou, G. Orderda, S. Kolacek, P. Urruzuno, M.J. Martínez-Gómez, T. Casswall, M. Ashorn, H. Bodanszky, F. Mégraud on behalf of the European Paediatric Task Force on Helicobacter pylori . Prospective multicenter study on antibiotic resistance of Helicobacter pylori strains obtained from children living in Europe. Prospective multicenter study on antibiotic resistance of Helicobacter pylori strains. Gut 2006;55-1711-16
10. S. Sausenthaler, **S. Koletzko**, B. Schaaf, M. Borte, O. Herbarth, A. v. Berg, HE. Wichmann, J. Heinrich. Maternal diet during pregnancy in relation to exzema and allergic sensitization in the offspring at 2 years of age. Am J Clin Nutr 2007;85:530-7.

11. v. Berg, S. **Koletzko**, B. Filipiak-Pttroff, B. Laubereau, A. Gruebl, H. E. Wichmann, C.P. Bauer, D. Reinhardt, D. Berdel for the GINI Study Group. Certain cow's milk hydrolysates reduce the risk for atopic eczema, but not for asthma in the first three years of life: results of the German Infant Nutritional Intervention Study GINI, a randomized trial J Allerg Clin Immunol 2007 (in press)

A.2 Sub-contracting

Danone will provide the placebo (lactose, Pharmatose) and the intervention product (Glutival obtained from Cargill). Danone has outsourced the production of the sachets with the gluten intervention / placebo products to NIZO Food Research, Ede, the Netherlands.

NIZO will prepare and package the gluten intervention product as well as the placebo according to quality criteria adequate for infants aged 4-6 months. Every intervention sachet will be filled 1.8 gr Pharmatose and 0.2 gr Glutival (gluten intervention product); the placebo sachets will be filled with 2 gr of Pharmatose.

NIZO will pay special attention to the microbiological assessment of the products and will make sure that the results will be in accordance with the standards of Danone. Also it will be analytically verified whether the blending of the materials has been adequate and resulted into a homogeneous mixture. The costs made by NIZO will be paid by the budget of Danone, the estimated costs are € 60.000,-.

A.3 Third parties

Professor Moshe Berant, chairman of the Helsinki Committee at Rambam Medical Center and member of the Steering Committee of the Israeli Ministry of Health for Regulatory Issues and Monitoring of Clinical Trials in Human Subjects (annex 10.3 CV) will participate as ethical advisor. The ethical advisor will give advise, asked and unasked, to the Management team of the project with respect to the ethical questions and decisions.

The legal status of the University Hospital of the Ludwig Maximilians University (partner 17, LMU) has changed. Part of the PreventCD work is forseen to be carried out by Ludwig-Maximilians-University (LMU Muenchen) through resources provided by Klinikum der Universitaet Muenchen.

Beneficiary LMU Muenchen reimburses the costs of third party Klinikum der Universitaet Muenchen, Marchioninistraße 15, 81377 Muenchen, Germany.

The financial contribution involved is not changed, for detailes see the table below:

WP N°	Workpackage title	Research activity	Indirect costs	EU requested contribution
WP3	European early feeding intervention study in infants from high risk families for coeliac disease (CD)	138.00	27.760	
Total				166.560

A.4 Funding of third country participants
Not relevant – there are no third country participants
